# Diversity of Light Sensing Molecules and Their Expression During the Embryogenesis of the Cuttlefish (*Sepia officinalis*)

**DOI:** 10.3389/fphys.2020.521989

**Published:** 2020-09-29

**Authors:** Morgane Bonadè, Atsushi Ogura, Erwan Corre, Yann Bassaglia, Laure Bonnaud-Ponticelli

**Affiliations:** ^1^Laboratoire Biologie des Organismes et Ecosystèmes Aquatiques, Muséum National d’Histoire Naturelle, Sorbonne Université, Centre National de la Recherche Française (FRE2030), Université de Caen Normandie, Institut de Recherche pour le Développement (IRD 207), Université des Antilles, Paris, France; ^2^Department of Computer Bioscience, Nagahama Institute of Bio-Science and Technology, Nagahama, Japan; ^3^Station biologique de Roscoff, plateforme ABiMS, FR2424 CNRS-Sorbonne Université (UPMC), Roscoff, France; ^4^Université Paris Est Créteil-Val de Marne (UPEC), Créteil, France

**Keywords:** opsin, cryptochrome, arrestin, eye, development, *Sepia officinalis*

## Abstract

Eyes morphologies may differ but those differences are not reflected at the molecular level. Indeed, the ability to perceive light is thought to come from the same conserved gene families: opsins and cryptochromes. Even though cuttlefish (Cephalopoda) are known for their visually guided behaviors, there is a lack of data about the different opsins and cryptochromes orthologs represented in the genome and their expressions. Here we studied the evolutionary history of opsins, cryptochromes but also visual arrestins in molluscs with an emphasis on cephalopods. We identified 6 opsins, 2 cryptochromes and 1 visual arrestin in *Sepia officinalis* and we showed these families undergo several duplication events in Mollusca: one duplication in the arrestin family and two in the opsin family. In cuttlefish, we studied the temporal expression of these genes in the eyes of embryos from stage 23 to hatching and their expression in two extraocular tissues, skin and central nervous system (CNS = brain + optic lobes). We showed in embryos that some of these genes (Sof_CRY_6_, Sof_reti-1, Sof_reti-2, Sof_r-opsin1 and Sof_v-arr) are expressed in the eyes and not in the skin or CNS. By looking at a juvenile and an adult *S. officinalis*, it seems that some of these genes (Sof_r-opsin1 and Sof_reti1) are used for light detection in these extraocular tissues but that they set-up later in development than in the eyes. We also showed that their expression (except for Sof_CRY_6_) undergoes an increase in the eyes from stage 25 to 28 thus confirming their role in the ability of the cuttlefish embryos to perceive light through the egg capsule. This study raises the question of the role of Sof_CRY_6_ in the developing eyes in cuttlefish embryos and the role and localization of xenopsins and r-opsin2. Consequently, the diversity of molecular actors involved in light detection both in the eyes and extraocular tissues is higher than previously known. These results open the way for studying new molecules such as those of the signal transduction cascade.

## Introduction

**FIGURE 1:**
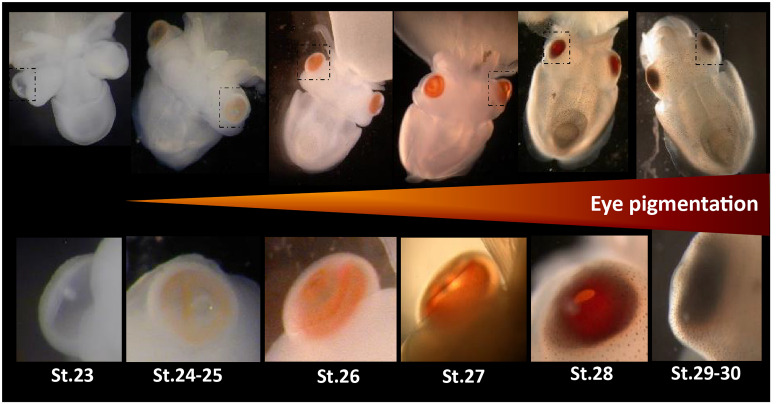
Eye development in *Sepia* embryos. 30 developmental stages of *Sepia officinalis* are described (Boletzky et al., 2016). Top: full embryos, bottom: magnification of the eyes. Eye pigmentation starts at stage 24–25 (light orange) then darkens until reaching a dark brown color at the time of hatching.

Eyes are specialized light-sensitive sensory structures, most of time involved in image forming vision. They can take a wide variety of shapes and the molluscan clade displays an amazing diversity of eye morphologies: pallial eyes in bivalves, cephalic eyes in gastropods, ocellus in chitons and camerular eyes in cephalopods. Cephalopods in general, and cuttlefish in particular, have been extensively studied for their visually guided behaviors. The visual system of coleoid cephalopods is mainly composed of two large spherical eyes with a lens, a vitreous cavity and an iris, known as camera (or camerular) eyes. They are linked to optic lobes through optic nerves. Optic lobes are located on each side, between the eye and the brain. They are involved in visual processing and visuomotor control and are essential for the transmission of light information to the brain (Boycott, 1961; Young, 1962, 1974). In *Sepia officinalis*, the two optic lobes represent about twice the size of the brain (Nixon and Young, 2003) and the eyes harbor rhabdomeric photoreceptor cells as in many protostomians. This visual system sets up during embryogenesis. This was described in *Sepiella japonica*: first the eyes vesicles are formed, then a light orange pigmentation starts to appear on the retina and darkens until reaching a dark brown color at the time of hatching. The photoreceptor cells appear from a differentiation of the retinal epithelium and are mature a little before hatching (Yamamoto, 1985). Furthermore, electrophysiological studies have shown that eyes of *S. japonica* embryos were already reacting to light before the final differentiation of the retina (Yamamoto et al., 1985). In *S. officinalis*, the macroscopic setting-up of the visual system is similar (Figure 1; Boletzky et al., 2016). Behavioural studies have shown that the embryo is able to answer a light stimulation as soon as the pigmentation starts to appear in the eyes (stage 25: Romagny et al., 2012). Indeed, this pigmentation is due to the presence of retinal in the rhabdomes: retinal is a chromophore that switches conformation when absorbing light, thus activating a light sensing molecule. Actually, at the molecular level, photoreceptor cells all contain light sensing molecules responsible of light detection. These light sensing molecules interact with a variety of other molecular actors, which either regulate their function or act as down-stream effectors to ensure the transduction of signals [depending on light sensing molecules reviewed in Yau and Hardie (2009) and Chaves et al. (2011)]. The transcription pathways of these molecules have just begun to be studied in cephalopods.

Most studies focusing on light sensing molecules in cephalopods have been done in adults whereas the embryos are able to perceive light suggesting the visual system is already functional in embryos (Romagny et al., 2012). Our work aims at identifying the light sensing molecules expressed in *S. officinalis* embryos in order to characterize the timeline and the putative correlation of the respective appearance of molecules and visual/photosensitive function. We focused on opsins and cryptochromes and we also study the “squid visual arrestin” (Yoshida et al., 2015) thought to be implicated in phototransduction in visual cells.

The opsin family is a multigenic family of G protein coupled receptors (GPCR) which can be found in most eumetazoans (Porter et al., 2011; Feuda et al., 2012; Ramirez et al., 2016). They bind retinal enabling light detection and have been studied in many taxa for their involvement in vision. Phylogenetic relationships of opsins are complex and a recent publication with large sampling across eumetazoans suggested at least 9 opsins paralogs in the ancestor of bilaterians (Ramirez et al., 2016). From these 9 groups of opsins, only 6 have been identified in molluscs (canonical r-opsins, non-canonical r-opsins, xenopsins, retinochromes, Go-opsins and neuropsins), 4 in cephalopods (canonical r-opsins, non-canonical r-opsins, xenopsins, retinochromes) but only 2 in *S. officinalis* (Ramirez et al., 2016). Most opsins are expressed in the eyes or in neural tissues, even if they may also be found in other tissues (Porter et al., 2011). In the pigmy squid (*Idiosepius paradoxus*), 5 sequences of opsins have been identified; all are expressed in the eyes but only r-opsin1 and retinochrome 1 (reti-1) seem eye-specific (Yoshida et al., 2015). The authors also documented a squid specific duplication of the retinochrome gene and identified for the first time a xenopsin in a cephalopod (firstly described as a c-opsin _ Yoshida et al., 2015; Ramirez et al., 2016). In adult *S. officinalis*, the spatial expression of the two known opsins has been studied: r-opsin1 (rhodopsin in the literature, Bellingham et al., 1998) and a retinochrome are expressed in the eyes and the skin (Mäthger et al., 2010; Kingston et al., 2015a). Indeed, r-opsins are known for their involvement in vision in many protostomians and retinochromes are thought to work together with them. Retinochromes have the ability to switch retinal back to its original conformation after its linkage with r-opsins thus allowing r-opsins to bind it and signal again. *In situ* hybridization also showed that r-opsin1 is expressed during late embryogenesis from stage 23 to hatching in the eye of *S. officinalis* embryos (Imarazene et al., 2017).

Cryptochromes belong to a family of molecules able to sense light in the blue and UV range. This family gathers photolyases and both animal and plant cryptochromes (which are not homologous). These flavoproteins are usually studied in the central nervous system (Benito et al., 2008). They have been detected in photoreceptor cells in the eyes of species far apart across the bilaterian phylogenetic tree (in amphibians: Zhu and Green, 2001; in insects: Yoshii et al., 2008; in mammals: Tosini et al., 2007; Nießner et al., 2016; in birds: Wiltschko and Wiltschko, 2014). They have been mainly studied for their involvement in non-visual light sensing roles such as control of circadian rhythm, compass navigation and maybe even magnetoreception [reviewed in Chaves et al. (2011)]. Recent studies in *Drosophila melanogaster* suggested that a cryptochrome could be able to interact with the phototransduction complex and that it would have an indirect role in vision by regulating the circadian plasticity of visual system sensitivity (Mazzotta et al., 2013; Mazzotta and Costa, 2016). Usually three families of animal cryptochromes are described: the Cry123 and the Cry45-Photolyase families which are found in all bilaterians and the Cry6 family which has only been described in protostomians (Oliveri et al., 2014; Haug et al., 2015). In molluscs, representatives of these three families have been described (Oliveri et al., 2014). Regarding cephalopods, the only published data focus on *Euprymna scolopes* (Heath-Heckman et al., 2013). In this species, two different cryptochromes (*escry1* and *escry2*) were identified with a daily cycling expression in the head for both of them.

The arrestin family is known for its ability to regulate signal transduction by interacting with GPCR. Several paralogs are known to be specifically expressed in the photoreceptor cells and to interact with visual opsins: S-Arrestin (or SAG) and Arrestin-C (or Arrestin-X) (Craft and Whitmore, 1995) in vertebrates and visual arrestins (also called phosrestines) in arthropods (Montell, 1999; Merrill et al., 2003). Recently a “squid visual arrestin”, specifically expressed in the eyes, was identified in three Decabrachia cephalopods (Mayeenuddin and Mitchell, 2003; Yoshida et al., 2015).

In this study, we identified and phylogenetically characterized in *S. officinalis* the light-sensitive molecules, opsins and cryptochromes, and one associated molecule, visual arrestin. We localized transcripts of these molecules through different technics in embryos, a juvenile and adults. Expressions were found in the eyes but also in other tissues with photosensitive properties (skin and CNS). The dynamics were established in the developing eyes by looking at the temporal expression of these genes in several late embryonic stages. We found diverse photosensitive molecules and have a better understanding of their evolutionary history in molluscs and in cephalopods. Our results allowed us to try to link their expression to the acquisition of visual function and photosensitivity before and after hatching.

## Materials and Methods

### Biological Samples, Dissection and Fixation

#### Embryos

*S. officinalis* eggs were all obtained from Roscoff marine station (CRB-Sorbonne Université-EMBRC, France) except the stage 30 embryo used for *in situ* hybridization (ISH), that comes from Caen (CREC station-Université Caen Normandie). The embryos used for RNA-seq were cultured in an open circulatory system with filtered sea water at 17°C and under natural light. The embryos used for RT-qPCR were kept in these same conditions except that the photoperiod was controlled with an alternating 12 h of light and 12 h darkness with a LED mimicking daylight. The embryo from Caen (for ISH) was kept for several weeks in a closed circulatory system, artificial sea water at 19°C and under natural light. Embryos were extracted from the chorion, in filtered seawater on ice in order to anesthetize the animals, the yolk was removed and they were staged (Boletzky et al., 2016). The fixation always took place from June to early August during day time (from 10 a.m. to 4 p.m.) under natural light.

For RT-qPCR experiment and RNA sequencing, the samples were immersed in RNA later and kept in RNA Later (SIGMA) at −20°C before being studied. Stages 23, 25, 28 and 30 were used for the RT-qPCR experiment and stages 24, 25, and 30 for the RNA sequencing. Prior to extraction, eyes were dissected and lens were removed, brain and optic lobes were dissected and samples of dorsal and ventral skin were taken.

For *in situ* hybridization, a late stage 30 embryo was fixed in 4% Paraformaldehyde (PAF-Formaldehyde- EMS, Hatfield) in PBS 1X at 4°C, 3 times 24 h. After being rinse in PBS 1X (3 times 10 min), it was dehydrated in 50% methanol/50% PBS 1X for 20 min, and in methanol 100% for 48 h at 4°C. The embryo was kept in methanol 100% at −20°C.

#### Juveniles

Eggs were obtained from Caen (CREC marine station-Université de Caen Normandie). They were maintained in a tank in a closed circulatory system of artificial seawater in controlled condition of temperature (19°C) until hatching. Hatchlings began to feed after 1 week, then juveniles were fed with alive or frozen preys until 1 month old in a tank equipped with structures adapted for animal welfare. They were placed on cold seawater with MgCl2 as an anesthetic. After several minutes, when no more reaction was observed, animals were immediately immersed in RNA later. Brain, eyes; skin, and other tissues were immediately taken and kept in RNA later.

#### Sub-Adults

Freshly fished specimens of *S. officinalis*, from Atlantic Ocean (Ile D’Yeu, France), that just died, were used. They were always kept on ice and were dissected on the boat: eyes, brains, optic lobes and skin were removed and placed in RNA Later. They were kept for 6 days at room temperature and maintained at −80°C until RNA extraction.

### RNA Sequencing

#### RNA Extraction

##### Embryos

Eyes, skin and central nervous system (CNS = brain + optic lobes) of embryos from stage 24, stage 25 (only eyes and CNS) and stage 30 were used. For each organ, two embryos were used per stage (=2 biological duplicates). EZNA Mollusc RNA extraction kit (Omega bio-tek) was used with an on-membrane DNAse I (Qiagen) treatment and tissues were disrupted using lysis buffer from the kit and vortex alone. RNAs were eluted in RNAse-Free water at 65–70°C. Quantity and quality were assessed with Qubit 3 fluorometer (Invitrogen), Nanodrop^TM^ (ThermoScientific) and Bioanalyzer 2100 (Agilent). Finally, RNAs were stored at −80°C before use.

##### Juvenile

Brain, skin, eye for one specimen and overall body and shell sacs for three specimens were used. Tissue pieces were homogenized using needle in TriZol reagent (Life Technologies, Carlsbad, CA, United States) and the suspension was applied on Qiashredder column (Qiagen), and deproteinized with chloroform. Supernatant was applied on a gDNA eliminator column (Qiagen) to eliminate DNA, and RNAs were purified using Rneasy plus mini, midi or micro kit (Qiagen) depending on the weight of the tissue. RNAs were kept in water, the quality evaluated by Nanodrop^TM^ (ThermoScientific) and sent for sequencing.

#### Sequencing and Assembling

##### Embryos

RNAs were sequenced by BGI Inc., using Illumina HiSeq 2000 technology according to usual protocol. The 457.6 million clean short reads sequences obtained (ranging from 21.2 to 39.9 million/sample; Average Q20 = 96.4%) were pulled to one dataset and assembled *de novo* using Trinity (v2.8.4) (Grabherr et al., 2011) with quality trimming by Trimmomatic package (Bolger et al., 2014) forming 673645 contigs (N50 = 921). Expression frequencies were calculated after read remapping using bowtie2 (Langmead and Salzberg, 2012) on RSEM (v1.3.1) (Li and Dewey, 2011). Therefore normalized data representing the intensity of gene expression across samples (FPKM = fragments per kilobase per million reads) were obtained. The whole assembled sequences were also blasted using Diamond against NR and Uniprot in order to identify putative genes of interests. As we have duplicates, statistical analysis were done using Edger package in R with a FC threshold of 0.5 as recommended in literature for *n* ≤ 3 (Schurch et al., 2016). We only considered results with a *p*-value < 0.01.

##### Juveniles

Synthesis of cDNA, library construction, Illumina sequencing and generation of FASTQ raw files were achieved by the sequencing platform of EUROFINS Genomics. Briefly, libraries were prepared using a HiSeq RNA sample preparation Kit (Illumina Inc., San Diego, CA, United States) according to the manufacturer’s instruction. One lane was multiplexed for 12 samples and was sequenced as 125-bp paired-end reads using Illumina/Solexa technology (HiSeq 2500). For each library FASTQ file generation was performed by RTA v1.18.64.0 and CASAVA v1.8.2 software (Illumina). After quality assessment, trimming of adaptors, and filtering for low-quality reads (average QC < 30) with Trimmomatic v0.35, 230.2 million clean short reads sequences obtained (ranging from 14.85 to 25.92 million/sample; Q30 = 83.8 to 89.2%) were assembled with Trinity (v.2.2.1) leading to 586294 contigs (N50 = 594). After filtering transcripts weakly expressed (overall expression < 1 FPKM), a transcriptome with 93632 contigs was obtained (N50 = 748). Expression frequencies were calculated on RSEM (v1.3.1) (Li and Dewey, 2011) on the filtered transcriptome and were used for looking at the expression of our target genes in the skin, brain and eyes of the juvenile. A specific search on the unfiltered assembly for lowly expressed transcripts in brain, skin and eye libraries was done afterward. Reads mapping and expression analysis were conducted as previously described.

### Transcriptome Blasts and Phylogenetic Analysis

In our transcriptomes of embryos, we found 10 sequences of interest: 6 blasting with opsins sequences, 2 with cryptochromes and 2 with arrestins (NCBI accession numbers MN788446-50, MN788452, MN788454-56 and MN788460 _ for some sequences, alternative isoforms were found with few to no differences in the amino acids sequences and were not included in phylogenies). They were also retrieved from the juvenile transcriptome with 100% identity of the ORF nucleotide sequence (except 99.66% identity for r-opsin1 and only partial sequences for r-opsin2 (402nucl _ 100% id) and xeno2 (323 nucl. _ 99.66% id)). Two of the putative opsin sequences are partial (Sof_r-opsin2 and Sof_xeno2) but the corresponding full sequences could be retrieved from an already published transcriptome (Liscovitch-Brauer et al., 2017) and were used for the phylogenetic analysis. Using a p-blast algorithm in NCBI Blast putative homologous amino acids sequences in molluscs were retrieved. We used amino acids for the analysis as they are conserved because more constrained by their function. In order to confirm the orthology of these genes, sequences from taxa outside the molluscan clades (Annelida, Ecdysozoa, Deuterostomia) were added. We also blasted several genomes of cephalopods in NCBI (*Architeuthis dux* PRJNA534469, *Euprymna scolopes* PRJNA470951, *Octopus sinensis* PRJNA541812) and added data from transcriptomes of cephalopods (Liscovitch-Brauer et al., 2017). After a first alignment with MAFT (default parameters) implemented in JABAWS (Troshin et al., 2011) in the Jalview 2.11.0 software (Waterhouse et al., 2009) and manual trimming of sequences that were redundant or poorly aligned, datasets of 139 sequences (opsins), 50 sequences (cryptochromes) and 40 sequences (arrestins) were obtained (Supplementary Figure S1). For the opsin this was done by sub families and the results were then aligned all together using MAFT (L-INS-i and G-INS-i preset models). Finally the less conserved parts of the alignment were manually removed for all datasets and if necessary a last alignment with MAFT was done. It resulted on three alignments (Supplementary Figure S2) of, respectively, 311 aa (opsins), 402 aa (cryptochromes) and 325 aa (arrestins). ProtTest 3 (Guindon and Gascuel, 2003; Darriba et al., 2011) was used to define the better protein based evolution model to use for the phylogenetic analysis: respectively, LG + G + F (opsins), LG + G + I (cryptochromes) and LG + G + I (arrestins). A Bayesian inference tree was inferred using Mr. Bayes v3.2.7a (Ronquist et al., 2012) embedded in the CIPRES V 3.3 platform (2000000 generations, tree sampling frequency = 100, 4 Markov chains, 2 runs and burnin 25%). A maximum likelihood tree was also inferred (not shown) using RAxML-HPC v8.2.12 (Stamatakis, 2014) embedded in the CIPRES V 3.3 platform with similar results (Bootstrap values for conserved nodes are shown on the Bayesian phylogenetic trees _ 1000 bootstraps were performed).

### Cryo-Sections and *in situ* Hybridization

Embryo in methanol was impregnated in 0.12M phosphate buffer pH 7.2 with 15% saccharose at 4°C for twice 24 h. Then, it was included in Neg-50^TM^ embedding medium (Richard-Allan Scientific^TM^Thermo Scientific^TM^) and blocks were frozen in 60 s at −80°C with PrestoCHILL (Milestone). Sections of 20 μm were performed using cryostat (HM560MV-Thermoscientific, France).

After 30 min at room temperature, the sections were rehydrated twice 15 min in PBS 1X followed by 15 min in SSC 5X. In a humid chamber, slides were prehybridated in hybridization solution (HS: 50% deionized formamide, 5X standard saline citrate, 40 μg/ml salmon sperm DNA, 5X Denhardt’s, 10% dextran sulfate) for 2 h at 65°C before being incubated overnight with antisens probes (100 ng/mL) labeled with digoxigenin. Sense probes were also tested as negative controls. Sections were rinsed at 65°C: twice in SSC 2X for 30 min and 1 h and in SSC 0.1X. At room temperature, sections were treated with MABT (100 mM maleic acid, 150 mM NaCl, 1% tween20, pH 7.5) twice 15 min. Saturation was performed for 1 h in blocking solution (MABT, 4% blocking powder (Roche), 15% fetal bovine serum), followed by incubation for 1 h at 4°C with anti-digoxigenin antibodies (Roche) coupled to alkaline phosphatase (AP) and diluted at 1:500 in blocking solution (MABT, 1% blocking powder, 5% fetal bovine serum). Excess antibody was eliminated by 4 rinses in MABT (30 s, twice 45 min and overnight). Sections were impregnated for 20 min in AP solution (100 mM tris–HCL, 50 mM MgCl_2_, 0.1% tween20) with 1 mM levamisole hydrochloride (Sigma). The revelation was conducted in the same solution containing 165 μg/ml BCIP (5-bromo-4-chloro-3’-indolyphosphate p-toluidine salt) and 330 μg/ml NBT (nitro-blue tetrazolium chloride) (Roche). The reaction was stopped by washing 2 times 20 min in PBS 1X. The slides were treated with DAPI (4’,6-diamidino-2- phenylindole; 100 μg/L). Sections were mounted in Mowiol.

The labeled cryo-sections were observed under a Leica DMRB microscope. Several pictures per slices were taken with a camera color Canon EOS 60D. Images were assembled and treated for contrast and brightness using Adobe Photoshop CS6 (Adobe, CA, United States).

### RT-qPCR

#### Extraction, DNase Treatment and Reverse Transcription

One embryo was used per biological sample. RNA was extracted using Nucleospin RNA mini kit (Macherey Nagel) with Type D Beads (Macherey Nagel) to disrupt tissues and on-membrane rDNAse treatment. RNA was eluted in RNAse-Free water at 65–70°C. Remaining gDNA was removed using Turbo Dnase (Ambion _ 2 UI/μL) at 37°C for 30 min and the solution was purified using RNA CleanUp kit (Macherey Nagel). Quantity and quality were assessed with Qubit 3 fluorometer (Invitrogen) and Bioanalyzer 2100 (Agilent). Finally, RNA was stored at −80°C before use.

Reverse transcription was done using Superscript III (Invitrogen) following the manufacturer’s instructions with the same amount of RNA (215 ng) for each sample. cDNA was stored at −20°C.

#### RT-qPCR

##### Embryos

Primers were designed using Primer-BLAST on the NCBI website. Elongation factor 1 (Ef1) and β-actin were used as reference genes. Both genes have already been used as reference genes in cephalopods (Ef1 in *Octopus minor*: Xu and Zheng, 2018; [QSIImage]-actin in *Sepiella* sp.: Cao et al., 2016; Song et al., 2017). The specificity of all primers used in this study was checked through a sequencing of a purified PCR product. Selected primers for each gene are given in Table 1. The RT-qPCR experiment was performed on an AriaMx Real-time PCR system (Agilent technology). The RT-qPCR mix includes 0.25 mM of both primers (20 mM each), 2 μl of Rnase Free water, 5 μl Brilliant II SybR© Green qPCR Master Mix (Agilent) and 2,5 μL of cDNA diluted at 1/20th in RNAse Free water per well. The PCR cycling program consisted of 10 min at 95°C, then 40 cycles of 30 s at 95°C, 30 s at 58°C and 30 s at 72°C and finally 30 s at 95°C, 30 s at 65°C and 30 s at 95°C before decreasing the temperature to room temperature. We used three different embryos per stage in order to have three independent biological replicates. For each embryo used, the two eyes were pulled together. Technical triplicates were systematically performed on all samples. The specificity of RT-qPCR was verified by looking at the melting curves and double-checked with an electrophoresis migration of the RT-qPCR product of at least 2 wells per genes. PCR primers efficiencies were evaluated through serial dilutions and ranged from 88% to 103%. The level of expression of Sof_xeno1, xeno2 and Sof_r-opsin2 was too low to calculate their primers efficiencies. The fold change for stages 25 to 30 where calculated using the mean Ct at stage 23 as a control. In order to normalize the results the geometric mean of reference genes was used for calculation of the fold change with the following formula (FC = Fold Change; E = Efficiency of considered target or reference gene; Target = target gene; Ref = reference gene):

**TABLE 1:** Primers for RT-qPCR experiment. Sof_β-actin and Sof_Ef1 (bold) were used as reference genes.

Abbreviation	Forward primer	Reverse primer	Amplicon size	Efficiency
**Sof_β-actin**	**GGTACCACCATGTTCCCTGG**	**GGACCGGACTCGTCATATTCC**	**197 nucl.**	97%*
**Sof_Ef1**	**TGCCAGGTGACAATGTTGGT**	**CAATGTGTGCAGTGTGGCAA**	**198 nucl.**	97%*
Sof_Cry_123_	ATCTTGGGAGGATGGAATGAAGG	CAACAGGACAGTAGCAGTGGAA	134 nucl.	100%*
Sof_Cry_6_	AGCTGTACTGTTTCCACGGAC	TTTCATCTCGCTCCTGCCAT	92 nucl.	103%
Sof_reti1	CAGTCACTTGGCGGGTCATA	AGTGCGGGCAGTAGCAATAA	91 nucl.	96%
Sof_reti2	TAGGTTGCTGTGTCTATGTCAGT	CAGCGATACAGGCCAAAAGA	117 nucl.	97%
Sof_r-opsin1	GAGTCCCTATGCTGTCGTGG	ACGGAGTAGATGAGTGGATTGTG	129 nucl.	100%
Sof_r-opsin2	CGTGCTCTTCTGTGCTGGAT	GTGACACACTTCGCCGCTAT	123 nucl.	N.A.
Sof_xeno1	TAAACGGAGCAATCGTCATCTTC	GCAATCAGAAAGTCGCACACA	100 nucl.	N.A.
Sof_xeno2	TTGGGCCTGACTTCCATCAC	GCGTACAATACACAACCGCC	134 nucl.	N.A.
Sof_β-arr	TATTGGGCCTCACCTTTCGC	CCTTGGAGCCTGGTTAGTGG	97 nucl.	N.A.
Sof_v-arr	CGCTAGGATTTGGATCTGGTGA	TTCCTTGGCTTCGGGTTTGA	98 nucl.	88%

*Indicates that the given efficiency is a mean of several experiments that were not done on the same plate. N.A. = Not Available.

$$F\,C=\frac{E^{\Delta \,C\,t\,\left(T\,a\,r\,g\,e\,t\right)}}{M\,e\,a\,n\,\left(E^{\Delta \,C\,t\,\left(R\,e\,f\right)}\right)}$$

Statistical analyses were performed with GraphPad Prism 8.0.1 Software (San Diego, CA, United States^1^). The normality of the distribution of Fold Changes was assessed through Shapiro-Wilk test, then one way ANOVA was performed with a Tukey’s multiple comparisons test for each gene (significance: *p* < 0.05).

##### Adults

the tissues were obtained from different specimens, the skin came from a different sub-adult than the brain and optic lobes. Only one sample was tested for each organ. Technical triplicates were performed. We considered that there was a significant expression of the gene when the amplification took place before the 28th cycle of RT-qPCR (Cq < 28). Other results were regarded as non-significative and could indicate either a low expression or an absence of expression.

## Results

### Identification and Characterization of Genes

#### Opsin Family

Besides the two opsins sequences already known in adult *S. officinalis*, we identified 4 new putative opsin sequences in transcripts from embryos. These 6 sequences all have features of GPCR. The presence of seven transmembrane domains was predicted using TMHMM server v.2.0 (Krogh et al., 2001; Supplementary Figure S3) for all sequences except r-opsin1. In this last sequence, the 7th transmembrane domain was not retrieved by the software but could be assumed due to amino-acids similarities (Supplementary Figure S4). All sequences have the two cysteines forming a disulphide bridge essential for GPCR stability (Supplementary Figure S4). Some other features of the rhodopsin family of GPCR were observed such as the chromophore/opsin predictive binding site which is a well-conserved lysine (K296 in the bovine rhodopsin sequence) and the E/DRY motif and NPXXY site both allowing interaction with G proteins (the latter is not conserved in all sequences) (Supplementary Figure S4). Sof_xeno1 and Sof_xeno2 sequences are divergent on the N-term of the amino acid chain (5 first transmembrane domains) but the C-terminal part is identical (last 2 transmembrane domains).

**FIGURE 2:**
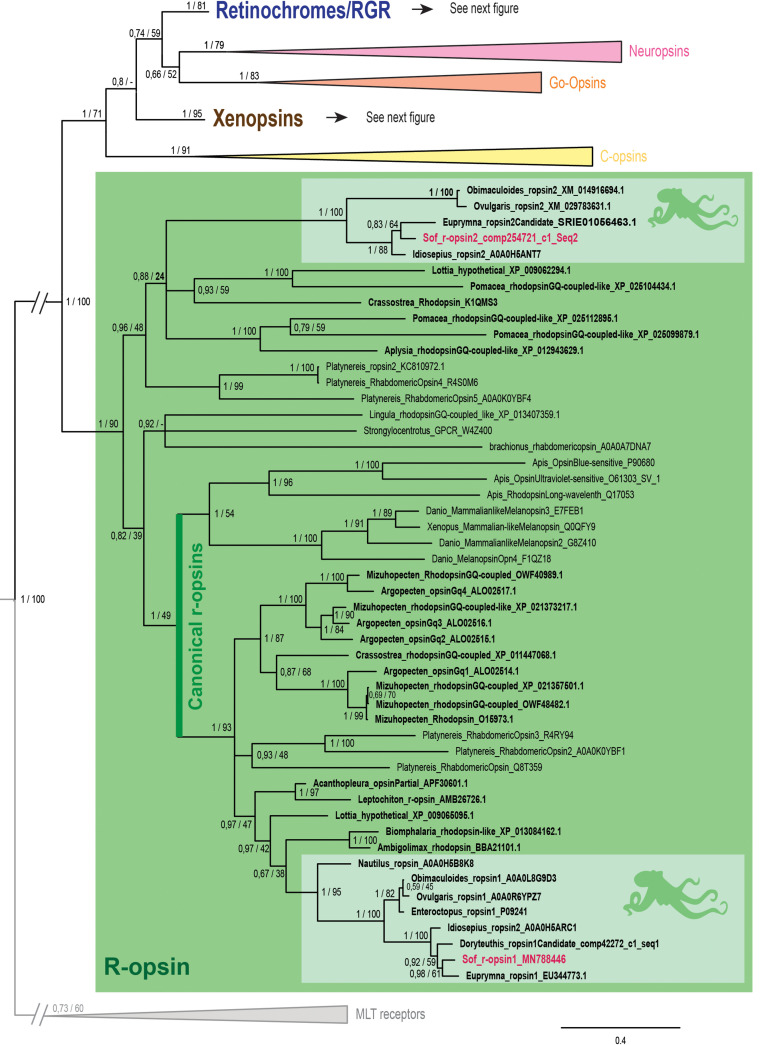
Opsin phylogeny with a focus on r-opsins. Phylogenetic analysis of 139 sequences of 311 amino acids generated in Bayesian Inference (MrBayes v3.2.7a). 6 main groups of opsins are retrieved: retinochromes (blue), neuropsins (red), Go-opsins (orange), xenopsins (brown), c-opsins (yellow) and r-opsins (green). Sequences from *S. officinalis* are in red, sequences from other molluscs are in bold and outgroups (Melatonine receptors (MLT)) are in gray. Dopamine receptors used for rooting the tree are not shown on the figure. Nodes labels are posterior probabilities and bootstrap values (PP/BS). Lighter boxes with cephalopod silhouette [modified from Stöger et al. (2013)] represent groups of cephalopod sequences. The branch from the root to the ingroups and outgroups were shortened for more lisibility (//).

**FIGURE 3:**
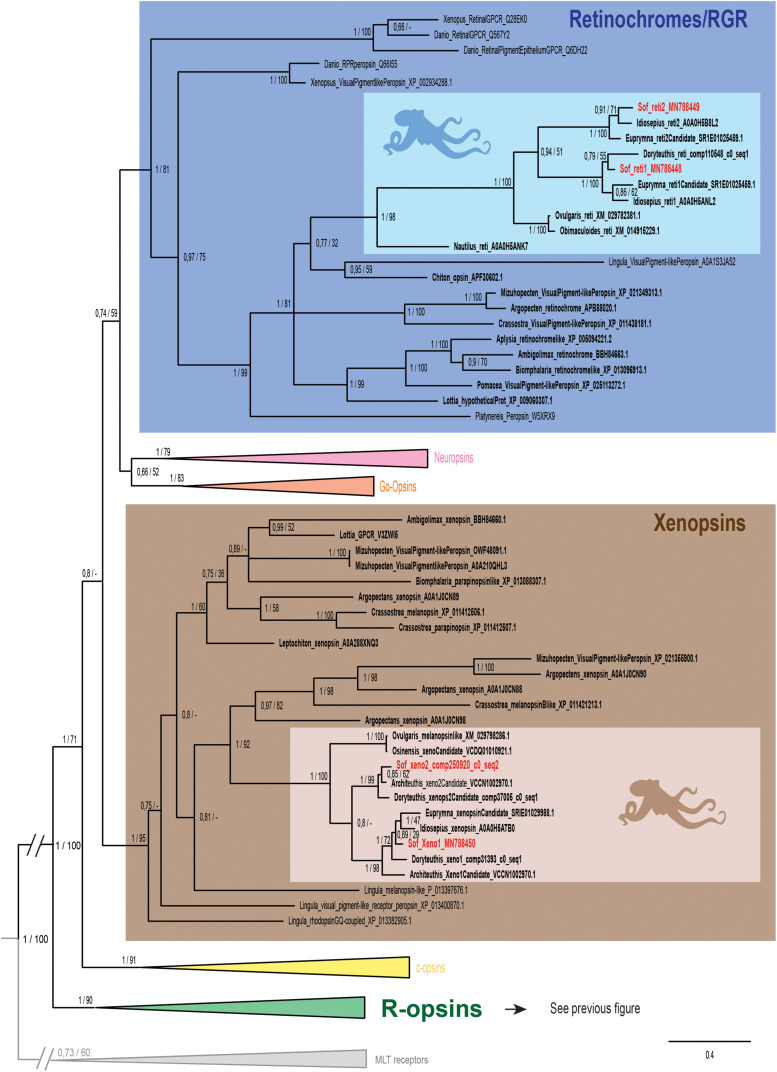
Opsin phylogeny with a focus on retinochromes and xenopsins. Phylogenetic analysis of 139 sequences of 311 amino acids generated in Bayesian Inference (MrBayes v3.2.7a). 6 main groups of opsins are retrieved: retinochromes (blue), neuropsins (red), Go-opsins (orange), xenopsins (brown), c-opsins (yellow) and r-opsins (green). Sequences from *S. officinalis* are in red, sequences from other molluscs are in bold and outgroups [Melatonine receptors (MLT)] are in gray. Dopamine receptors used for rooting the tree are not shown on the figure. Nodes labels are posterior probabilities and bootstrap values (PP/BS). Lighter boxes with cephalopod silhouette [modified from Stöger et al. (2013)] represent groups of cephalopod sequences. The branch from the root to the ingroups and outgroups were shortened for more lisibility (//).

In our phylogenetic analyses (Figures 2, 3) which included 7 groups of opsins (out of 9) and both dopamine receptors and melatonin receptors as outgroups we evidenced a monophyletic opsin family (Posterior Probabilities (PP) = 1/Bootstrap values (BS) = 100) thus confirming the identification of 6 opsins in *S. officinalis*. This opsin family was separated in 6 monophyletic groups (PP = 1 for all; BS between 79 and 95), three of which containing opsin sequences from *S. officinalis*. The general topology between the main families of opsins is not well-supported in this analysis.

The r-opsin clade (Figure 2 _PP = 1; BS = 90) is divided in two monophyletic clades each containing a sequence from *S. officinalis*. The biggest r-opsin group has a good support in the Bayesian inference tree (PP = 1; BS = 49). It is composed of a large lophotrochozoan r-opsin group (PP = 1; BS = 93) with both ecdysozoan r-opsins and deuterostomian melanopsins (PP = 1; BS = 54) as a sister-group thus characterizing it as a canonical r-opsin clade. Inside this group, the sequence from *S. officinalis* is included in a cephalopod r-opsins clade (PP = 1; BS = 95). The other cephalopod r-opsin group, which includes a second *S. officinalis* sequence, is well-supported in the Bayesian tree (PP = 1; BS = 100). It is included within a larger clade of lophotrochozoan sequences thus characterizing it as a second r-opsin (“non-canonical” r-opsin _ PP = 0.96; BS = 48). Therefore we chose to name these sequences Sof_r-opsin1 (former rhodopsin) and Sof_r-opsin2. This phylogenetic analysis allowed us to say that the division between these two paralogs was already there in the last common ancestor of all bilaterians. The bootstrap values are not as good as the posterior probabilities for the r-opsin1 and deuterostomian r-opsin clades, this might be due to some long branch attractions. In the literature, the “non-canonical r-opsin group” corresponding to our r-opsin2 group is sometimes paraphyletic (Ramirez et al., 2016).

Two other *S. officinalis* sequences (Figure 3) are grouped together with other cephalopod sequences (PP = 1; BS = 98) within a larger lophotrochozoan monophyletic group (PP = 1; BS = 99) and an even larger bilaterian group including annelids and deuterostomian retinochromes sequences (PP = 1; BS = 81). Therefore we named these two sequences Sof_reti1 and Sof_reti2. Thanks to the several sequences of cephalopods from various taxa (Nautiloidea, Coleoidea: Decabrachia and Octobrachia) included in our study, we evidenced that only decabrachian cephalopods have two retinochromes. All the reti1 sequences of Decabrachia were grouped together in a monophyletic group (PP = 1; BS = 100) and so are all the reti2 sequences of Decabrachia (PP = 1; BS = 100). These two groups seem to be sister-groups even though this clade is not well-supported (PP = 0.94; BS = 51). Furthermore only one monophyletic group of Octobrachia retinochrome sequences was found in our analysis (PP = 1; BS = 100) and it is the sister group of the clade formed by the two retinochromes groups thus suggesting the duplication event might have taken place after the splitting between Decabrachia and Octobrachia lineages.

Finally, the last two opsins sequences identified in *Sepia* are part of a lophotrochozoan-only group composed of molluscan and brachiopods sequences (Figure 3 _ PP = 1; BS = 95). Most of them were recently identified as xenopsins (but some of them firstly described as c-opsins _ Yoshida et al., 2015). The sequences were therefore identified as Sof_xeno1 and Sof_xeno2. As for retinochromes, both the Decabrachia xenopsin 1 group and the Decabrachia xenopsin 2 group are monophyletic (PP = 1 for both; BS = 98 and 99). Furthermore there is a single clade gathering all the Octobrachia xenopsins sequences (PP = 1; BS = 100). This octobrachian clade is the sister-group of the decabrachian xenopsin clade. The support for this decabrachian clade, grouping both xenopsins 1 and xenopsins 2, is very low (PP = 0.8) and this topology is not found in the maximum likelihood tree. The duplication of the xenopsins took place in the Cephalopoda lineage but we cannot conclude when it precisely happened.

No sequence of *S. officinalis* or of any other cephalopod species was found in the three other clades of opsins (i.e., neuropsins, Go-opsins, and c-opsins).

#### Cryptochrome Family

We identified for the first time, two putative cryptochromes in the transcripts of *S. officinalis* embryos. They both carry the photolyase domain and the FAD binding domain which characterized animal cryptochromes (Chaves et al., 2011; Supplementary Figure S4) and were identified with the PFAM platform (El-Gebali et al., 2019).

**FIGURE 4:**
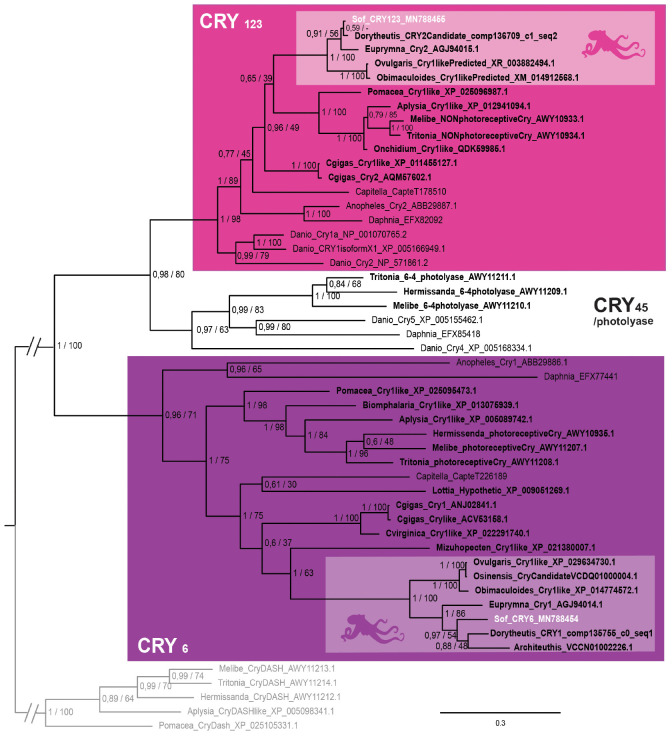
Cryptochrome phylogeny. Phylogenetic analysis of 50 sequences of 402 amino acids generated in Bayesian Inference (Mr. Bayes v3.2.7a). Three main groups of animal cryptochromes are retrieved: CRY_123_, CRY_45_/Photolyase and CRY_6_. Sequences from *S. officinalis* are in white, sequences from other molluscs are in bold font and outgroups used for rooting the tree (CRY-DASH) are in gray. Nodes labels are posterior probabilities and bootstrap values (PP/BS). Lighter boxes with cephalopod silhouette [modified from Stöger et al. (2013)] represent groups of cephalopod sequences. The branch from the root to the ingroups and outgroups were shortened for more lisibility (//).

In our phylogenetic analysis (Figure 4), we found three main monophyletic groups. One of the cryptochrome sequence of *S. officinalis* is grouped with other molluscan sequences, together with *Euprymna scolopes escry2* (Heath-Heckman et al., 2013) and the nudibranch *Melibe leonina* non-photoreceptive cryptochrome (Duback et al., 2018). This molluscan clade is part of a larger protostomian monophyletic group (PP = 1; BS = 89) and an even larger bilaterian monophyletic group (PP = 1; BS = 98) gathering all the CRY_1_, CRY_2_ and CRY_3_ sequences of our analysis. Therefore the sequence from *S. officinalis* was identified as Sof_CRY_123_. The second sequence from *S. officinalis* is found inside a well-supported lophotrochozoan monophyletic group (PP = 1; BS = 75). This group is part of a larger protostomian-only group including two arthropods CRY_6_ sequences (PP = 0,96; BS = 71) therefore we identified our second sequence as Sof_Cry6. Finally, a monophyletic group gathering a few molluscan photolyase sequences with deuterostomian Cry4 and Cry5 sequences can be identified as the CRY_45_/photolyase clade. No CRY_45_/Photolyase sequence was retrieved from *S. officinalis* transcriptomes nor from any other cephalopod included in our analysis.

#### Arrestin Family

**FIGURE 5:**
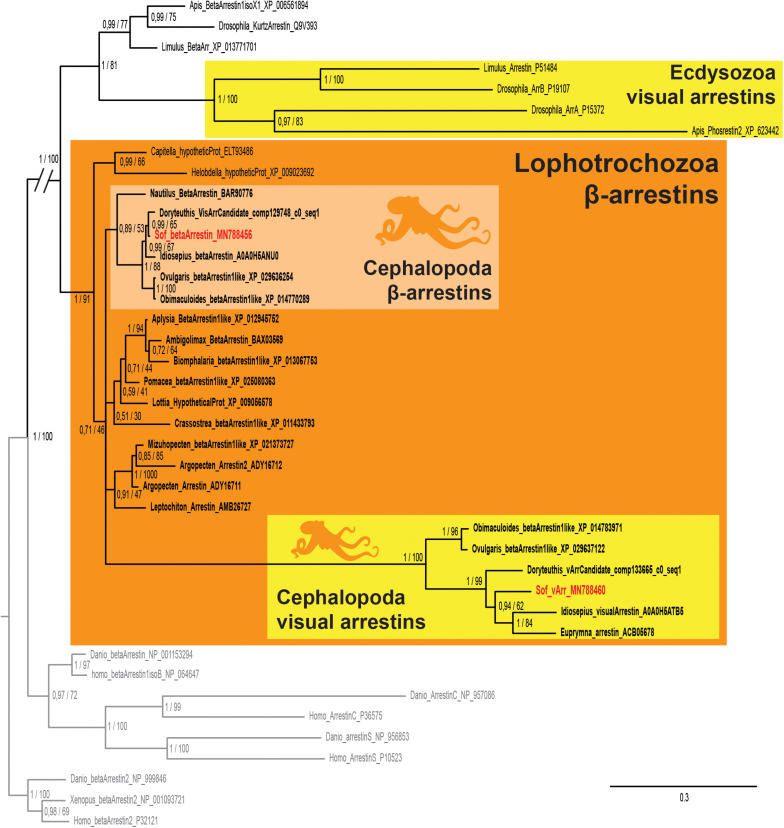
Arrestin phylogeny. Phylogenetic analysis of 37 sequences of 325 amino acids generated in Bayesian Inference (Mr. Bayes v3.2.7a). Two groups of coleoid cephalopods arrestins are retrieved: visual arrestins (v-arr) in yellow and β-arrestins (β-arr) in light orange. Ecdysozoan visual arrestins (or phosrestines) sequences are highlited (yellow), sequences from *S. officinalis* are in red, sequences from other molluscs are in bold font and outgroups (vertebrates β-arrestins 2 for rooting and other deuterostomians arrestins) are in gray. Nodes labels are posterior probabilities and bootstrap values (PP/BS). Lighter boxes with cephalopod silhouette [modified from Stöger et al. (2013)] represent groups of cephalopod sequences. The branch from the root to the ingroups and outgroups were shortened for more lisibility (//).

We found two sequences of arrestins in *S. officinalis* embryos. They both presented the canonical N- and C-arrestin domains identified through the PFAM platform (El-Gebali et al., 2019; data not shown). In our phylogenetic analysis, each of these sequences (Figure 5) is part of a distinct monophyletic group of coleoid cephalopod sequences. One of them gather only visual arrestins (PP = 1; BS = 100) and the other one only beta-arrestins (PP = 1; BS = 88). Relationships between these two groups and many sequences from other molluscs are not resolved. This politomy is part of a larger protostomian clade (PP = 1; BS = 100) including both arthropods beta arrestins and visual arrestins. All these sequences have deuterostomian arrestins as outgroups. Therefore our sequences derived from an ancestral β-arrestin. Visual arrestins sequences are only found in coleoid cephalopods and they are not orthologous to the visual arrestins of arthropods. From these results and present available databases, we cannot date the appearance of this duplication in molluscan history.

### Expression of Target Genes

#### Qualitative Expression in a Stage 30 Embryo With Focus on the Eyes

**FIGURE 6:**
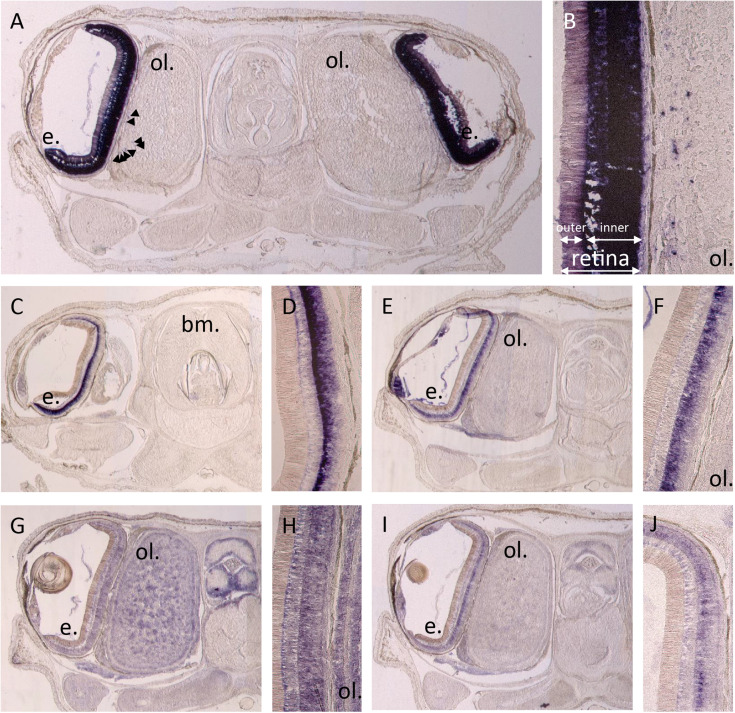
*In situ* hybridization of r-opsin1 **(A,B)**, retinochrome 1 **(C,D)**, retinochrome 2 **(E,F)** and cryptochromes: CRY_123_ **(G,H)** and CRY_6_ **(I,J)**. Pictures **(A,C,E,G,I)** show a 20 μm section of an embryo head with brain (not on section **A,C**), optic lobes (not on section **C**) and eyes including retina and sometimes the lens. Pictures **(B,D,F,H,J)** are a magnification focused on the retina ± the side of the optic lobes. All pictures were taken after 11 h of revelation except CRY_123_ pictures which were taken after 5h30. Arrows point some small stained spots. e. = eye, ol. = optic lobe, bm. = buccal mass.

By *in situ* hybridization, we evidenced expression of different photosensitive molecules (r-opsin1, both retinochromes, both cryptochromes _ Figure 6) in a late stage 30 embryo of *S. officinalis*: R-opsin1 mRNAs are found in all layers of the retina and in the periphery of the optic lobes (Figures 6A,B). No expression was evidenced in the brain as previously described (Imarazene et al., 2017). Both retinochromes mRNAs were found in the basal part of the inner layer of the retina where nucleus of the rhabdomeric photoreceptor cells is present (Figures 6C–F). CRY_123_ transcripts were widely present: mRNAs were found in the whole inner layer of the retina and in the whole central nervous system with a diffuse pattern in the optic lobes and in the brain (Figures 6I–J). CRY_6_ mRNAs were mostly present in the basal part of the inner layer of the retina (Figures 6G,H). They also seemed to co-localize with CRY_123_ mRNAs in the CNS: a diffuse labeling (lighter than the one of Sof_CRY_123_) was observed in some sections in the optic lobes and in the brain, long after the staining of the retina.

#### Semi-Quantitative Data in the Eyes (RNA-Seq)

**FIGURE 7:**
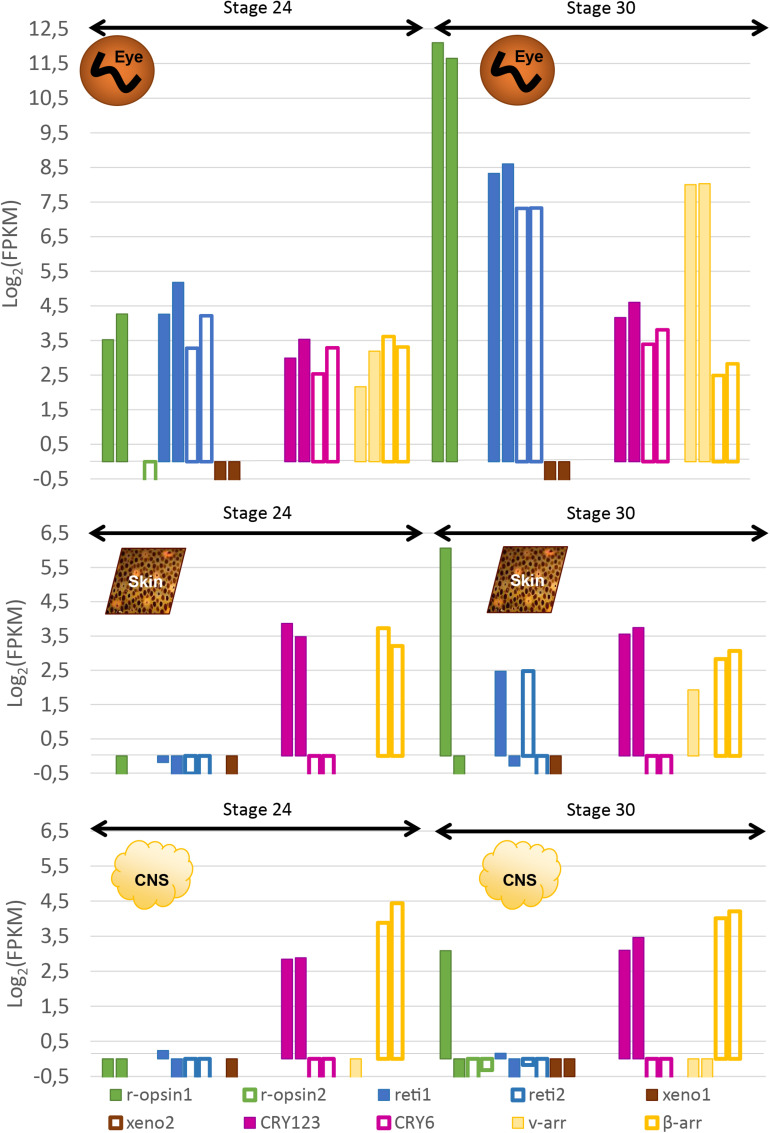
Expression of light sensitive molecules in the eyes, skin and CNS of *Sepia* embryos. Expression is given in Log_2_(FPKM) and are normalized with RSEM in order to compare between stage 24 and stage 30 in the eyes **(top)**, the skin **(middle)** and in the central nervous system (brain + OL _ **bottom**). For each gene, there are two bars each giving the expression in one of the two biological duplicates. The expression level of several isforms were summed up for some of the genes.

In order to confirm the qualitative data, we looked at the expression of these genes with RNA-sequencing: at stage 24, three opsins were expressed in the eyes of *S. officinalis* embryos according to our transcriptomic analysis (Figure 7): Sof_r-opsin1, Sof_reti1 and Sof_reti2. No significant expression of Sof_r-opsin2, Sof_xeno1 or Sof_xeno2 was observed (FPKM ≤ 1). Both Sof_CRY_123_ and Sof_CRY_6_ were expressed in this stage and so are Sof_v-arr and Sof_β-arr. For each of these genes the level of expression was similar in the two biological replicates. The expression of these genes ranged from FPKM_Sof_v–Arr_ = 6.8 to FPKM_Sof_reti__1_ = 28. In stage 30 embryos, the same genes were expressed. The highest level of expression was found for Sof_r-opsin1 (mean FPKM = 3827) followed by Sof_reti1, Sof_reti2 and Sof_v-arr (respectively, mean FPKM = 352, 160 and 258). Both cryptochromes and β-arrestin had a lower level of expression than opsin genes (mean FPKM Sof_CRY_123_ = 21; Sof_CRY_6_ = 12; Sof_β-arr = 12). Even though we have to be cautious when drawing conclusion due to small amount of replicates, a significant increase of Sof_r-opsin1, the two retinochromes and Sof_v-arr was found between stage 24 and 30.

#### Semi-Quantitative Expression in the Eyes, Through Developmental Stages (RT-qPCR)

**FIGURE 8:**
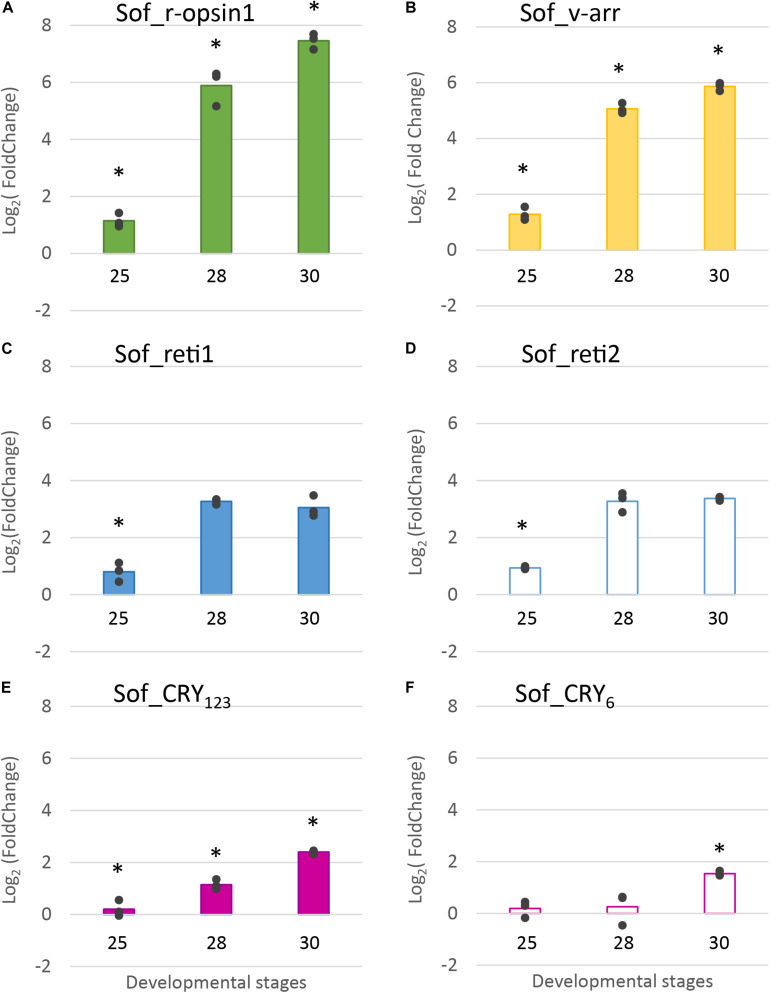
Differential expression of light sensitive molecules at several embryogenic stages. Genes considered are: **(A)**. R-opsin1, **(B)**. visual arrestin **(C)**. retinochrome 1 **(D)**. retinochrome 2 **(E)**. CRY_123_ and **(F)**. CRY_6_. The average log_2_ Fold Change for the three developmental stages (stage 25, stage 28 and stage 30) is given in comparison to stage 23. In addition, all data points are plotted to show actual variability.* = results significantly different from the other (*p* = 0.05).

RT-qPCR results (Figure 8) confirmed the increase of expression of Sof_r-opsin1, Sof_reti1, Sof_reti2 and Sof_v-arr and evidenced the increase of expression of the two cryptochromes. These expressions increased significantly between stage 25 and stage 30. Except for the cryptochromes, all the other genes had the biggest fold change between stages 25 and 28. Similar results were found between biological replicates for all the target genes. Sof_r-opsin1 (log_2_FC_23__–__30_ = 7,46) and Sof_v-arr (log_2_FC_23__–__30_ = 5,87) expressions underwent a drastic increase from stage 25 to stage 30. The increase was less important for both retinochromes (log_2_FC_23__–__30__Reti1 = 3,11; log_2_FC_23__–__30__Reti2 = 3,37) and both cryptochromes (log_2_FC_23__–__30__*Cry*_123_ = 2,4; log_2_FC_23__–__30__*Cry*_6_ = 1,54). R-opsin1 expression increased from stage to stage (Figure 8A) (log_2_FC_25__–__28_ = 4,74; log_2_FC_28__–__30_ = 1,57). Visual arrestin pattern of expression also increased from stage 25 to 30 (log_2_FC_25__–__28_ = 3,78; log_2_FC_28__–__30_ = 0,8). The expression of both retinochromes increased from stage 25 to 28 (log_2_FC_25__–__28__Reti1 = 2,47 and log_2_FC_25__–__28__Reti2 = 2,34), but not between stage 28 and 30. The expression of Sof_Cry_123_ increased gradually from stage to stage with the sharpest increase between stage 28 and 30 (log_2_FC_28__–__30_ = 1,26) whereas Sof_Cry_6_ expression seemed to be constant from stage 25 to 28 then increased between stage 28 and 30 (log_2_FC_28__–__30_ = 1,28).

In summary, we showed that five light sensing molecules (3 opsins and 2 cryptochromes) and one visual arrestin were expressed in the eyes of *S. officinalis* embryos. All the photosensitive molecules studied in RT-qPCR had a significant increase of their expression between stage 25 and 30.

#### Semi Quantitative Expression in Other Tissues With Photosensitive Properties (RNAseq)

Sof_CRY_123_ and Sof_β-arr seemed to have quite an ubiquitous expression as they are the only ones expressed at stage 24 in both the skin and the CNS (Figure 7). Their level of expression was not significantly different in stage 24 compared to stage 30 (CRY_123__:_ meanFPKM_Skin__24_ = 12,86, meanFPKM_Skin__30_ = 12,57, meanFPKM_CNS__24_ = 7,27, meanFPKM_CNS__30_ = 9,8; β-arr: meanFPKM_Skin__24_ = 11,25, meanFPKM_Skin__30_ = 7,74, meanFPKM_CNS__24_ = 18,25, meanFPKM_CNS__30_ = 17,31). Sof_CRY_6_ is not significantly expressed (FPKM ≥ 1) in these tissues at stage 24 and 30. R-opsin1, the two retinochromes and Sof_v-arr were expressed, only in one of the two stage 30 embryos in the skin. Moreover, in the central nervous system, r-opsin1 was also expressed in the same embryo and not in the other one. Because there are two samples, no conclusion can be drawn: either the differences are due to different steps in the stage 30, or to a cyclic expression during the day; or there is no biological significance and it corresponds to a random artifact. Neither Sof_xeno1 nor Sof_r-opsin2 were expressed at significant levels in any of the stages or tissues analyzed. Sof_xeno2 was not expressed in any stages except for one of the biological replicates at stage 25 (FPKM = 5.767).

#### Expression in the Juvenile (RNA-Seq)

In the eye of the juvenile (Table 2), three opsins (Sof_r-opsin1, Sof_reti1 and Sof_reti2), both cryptochromes and both arrestins are expressed, similarly to the observations in embryos from stage 23 to 30. In the skin, there is a small expression of Sof_CRY_123_ and Sof_β-arr as in the skin of the embryos. There is also an expression of Sof_r-opsin1 and a barely significant expression of Sof_reti1. In the brain, Sof_CRY123 and Sof_β-arr are the two genes mainly expressed in the brain of this juvenile as they were in the embryos, with the precision that there is no optic lobes studied in the juvenile (whereas CNS included both brain and optic lobes in embryos). There is a bare expression of Sof_r-opsin1, Sof_reti1, and Sof_CRY6 in the brain. As for sof_r-opsin2 and Sof_xeno1, these genes were absent from the filtered transcriptome but a weak expression was found when remapping the brain reads to the full assembly. These results need to be taken cautiously as they come from a single sample and cannot be compared to the other FPKM values of the filtered transcriptome.

**TABLE 2:** Expression of light sensitive molecules in a 1-month old juvenile.

	R-opsin1	R-opsin2	Reti1	Reti2	Xeno1	Xeno2	CRY_123_	CRY_6_	β-arr	V-arr
Eye	7572	Ø	362	59	Ø	Ø	10	6	6	344
Skin	10	Ø	1.3	0.2	Ø	Ø	7	0.5	9	0.1
Brain*	4	+	1.6	0.7	+	Ø	20	2	45	0.2

Data are expressed in FPKM. * = Brain without optic lobes (in embryos and adult the full CNS was investigated). + = transcripts absent from the filtered version of the juvenile transcriptome but detected with weak expression (1 < FPKM < 2) in the brain libraries in the unfiltered transcriptomes.

#### Expression in the Adult (RT-qPCR)

In the sub-adult (Table 3), Sof_ β-arr, Sof_ CRY_123_ and Sof_reti1 were also significantly expressed in the skin. Sof_r-opsin1, Sof-r-opsin2, Sof_reti1, Sof_β-arr and both cryptochromes were expressed in the CNS (brain and optic lobes) of the sub-adult. A significant expression of Sof_v-arr and the two xenopsins was also attested in the optic lobes only. The fact that some of these genes are found expressed in the sub-adult and not in the juvenile might be due to an increase of expression later in life.

**TABLE 3:** Expression of light sensitive molecules in sub-adults.

	R-opsin1	R-opsin2	Reti1	Reti2	Xeno1	Xeno2	CRY_123_	CRY_6_	β-arr	V-arr
Skin	N.S.	N.S.	+	N.S.	N.S.	N.S.	+	N.S.	+	N.S.
Brain	+	+	+	N.S.	N.S.	N.S.	+	+	+	N.S.
Optic lobes	+	+	+	N.S.	+	+	+	+	+	+

*+ = presence of the gene (amplification before Cq < 28), N.S. = Non-significative results indicating either a small expression or an absence of expression.*

## Discussion

### Localization of Expression

In our study we combined three different kind of data on the expression of light sensing molecules: qualitative *in situ* hybridization, semi-qualitative RT-qPCR and RNA-seq analyses. The results given by these different methods are mostly convergent. All methods found r-opsin1, two retinochromes, two cryptochromes and two arrestins expressed in the eyes of *S. officinalis* embryos and juvenile. This corroborates the *in situ* hybridization data on the expression of r-opsin1 in the retina of embryo (Imarazene et al., 2017) and the reported expression (RT-PCR) of both r-opsin1 and retinochrome1 in the retina of adult *S. officinalis* (Kingston et al., 2015b). Furthermore a personal communication from Maria Tosches confirmed the presence of Sof_r-opsin1, Sof_reti1 and Sof_reti2 in an unpublished transcriptome of the eyes of an adult *S. officinalis*. In the skin, only Sof_CRY_123_ and Sof_β-arrestins were expressed in the stage 24 embryo and one of the stage 30. These same genes were expressed together with Sof_r-opsin1 and Sof_reti1 in the other stage 30 embryo, the juvenile and the adult (only Sof_reti1 in the adult). In the CNS both RNA-seq and RT-qPCRs found Sof_CRY_123_ and Sof_β-arrestins as the two mainly expressed genes of our analysis in embryos and the juvenile. More genes were expressed in the brain of the adult and even more in the optic lobes of the adult. Our comparative data did not show important variability from a biological duplicate to another except in the two stage 30 embryos of the RNA sequencing.

One main difference is the fact that CRY_6_ is not expressed in the central nervous system of stage 30 embryos but was labeled in the *in situ* hybridization. As there is a small expression of Sof_CRY_6_ in the brain of the juvenile this might mean that the expression start in very advanced stage 30 embryos as the one we used for *in situ* hybridization was very close to hatching. We cannot rule out the fact that this difference of expression might be due to a pattern of daily cycling of the gene as the expression of cryptochromes is known to oscillate during the day (in nudibranchs: Duback et al., 2018; in Crustacea: Biscontin et al., 2019).

### Vision in Embryos and Adult

Two main opsin families have been well-studied for their involvement in image-forming vision: c-opsins and r-opsins. We did not find any c-opsins from our transcriptomes. This was expected as c-opsins are mostly restricted to deuterostomians and were not described in molluscs (Ramirez et al., 2016). We showed here for the first time that two r-opsins are expressed in *S. officinalis* but only Sof_r-opsin1 is expressed in the eyes. More precisely, r-opsin1 mRNAs could be found in all the retina but this is likely due to the fact that it is heavily translated in the nucleus of the rhabdomeric photoreceptor cells and then moved to the outer segment. Indeed in adult *D. pealeii*, the protein is localized only on the outer segment of the rhabomes (Kingston et al., 2015b). The expression of Sof_r-opsin1 increases significantly from stage 25 to 30. This strong increase of Sof_r-opsin1 mRNAs corroborates the qualitative *in situ* hybridization data already published about *S. officinalis* eye development (Imarazene et al., 2017) but also correlates with the appearance and darkening of pigmentation macroscopically visible in the eyes of the embryo (Figure 1). The biggest fold change value is found when comparing stages 25 and 28. This is convergent with the behavioral studies demonstrating an ability to perceive light as early as stage 25 in *S. officinalis* embryos (Romagny et al., 2012). Therefore, r-opsin1 is most likely the molecule responsible for this light detection. Moreover, r-opsin1 expression is still very high in juvenile and adult suggesting a “permanent” role in light detection/vision after hatching.

Our results support the hypothesis that cephalopods cannot see colors as only one opsin, i.e., r-opsin1, is expressed in their eyes. Nevertheless a color-based vision has been described in a butterfly expressing only one opsin with the involvement of filtering pigments (Zaccardi et al., 2006). This color vision may also exist in some cephalopods species as three different visual pigments with different λmax where identified in the retina of the deep-sea squid *Watasenia scintillans* (Matsui et al., 1988). To our knowledge, it is not the case in *S. officinalis* and in most cephalopod species and therefore the ability to see colors in cephalopods would be an exception rather than a rule. Recently some scientists have proposed that cephalopods (and maybe other marine animals) might rely on chromatic aberration and pupil shape in order to discriminate colors (Stubbs and Stubbs, 2016).

We have shown that the expression of Sof_CRY_6_ in the developing eye of *S. officinalis* is eye-specific. For a very long time cryptochromes were discarded from a role in visual function; however, this vision is challenged by recent publication on *D. melanogaster* showing that cryptochrome is able to interact with elements of the phototransduction cascade and has an indirect role in vision by regulating the light sensitivity of opsins during the day (Mazzotta et al., 2013). Here, we showed that Sof_CRY_6_ is expressed together with r-opsin1 only in the eye of *Sepia* embryos whereas in the juvenile and the adult it is also expressed in the CNS. This correlation is not enough to conclude but allows an interesting hypothesis regarding a role of CRY_6_ in the phototransduction cascade in cephalopods. In the literature, an oscillating expression of period protein was evidenced in the eye of two different marine gastropods species (*Bulla* and *Aplysia*) indicating the likely existence of a Clock system within the eyes of these species (Siwick et al., 1989). In this context, Sof_CRY_6_ expression could indicate that a clock system exists in the eyes of cephalopods and that it is already functioning in embryos.

### Clock System

Our analysis shows the presence of two cryptochromes expressed in different tissues. CRY_123_ is expressed in all the tissues investigated in embryos and in the juvenile and adult. CRY_6_ is expressed in the central nervous system only in the juvenile and the adult. The *in situ* hybridization shows a diffuse presence of CRY_123_ mRNAs in the retina and nervous tissues of the head of a stage 30 embryos and a lighter but similar pattern is found for CRY_6_ mRNAs. The fact that both a photosensitive cryptochrome (i.e., CRY_6_) and a non-photosensitive cryptochrome (CRY_123_) could be co-expressed and work together to maintain a circadian rhythm is a recent discovery (Zhu et al., 2005). The mechanism of this Clock system has just been unraveled recently in ecdysozoans (Zhu et al., 2008; Biscontin et al., 2017). It is important to note that organs were collected in daylight. In order to go further on the involvement of the cryptochrome in the control of the circadian rhythm it would be interesting to compare the expression of cryptochromes and other genes involved in daily cycling in organs collected at different time of the night and day.

### Retinochromes and Their Role

We identified two retinochromes expressed in the eyes of *S. officinalis* and we confirmed the hypothesis of a duplication limited to the Decabrachia clade (see results and Yoshida et al., 2015). For the first time we studied their expression during embryogenesis: the maximum fold change for retinochromes in *Sepia* embryos (between stage 25 and 28) correlates with the ones of Sof_r-opsin1 and Sof_v-arr. Furthermore both retinochromes mRNAs are found in inner layer of the retina, most likely in the cellular bodies of rhabdomeric photoreceptors cells as it was already reported for the protein of retinochrome1 in *D. pealeii* (Kingston et al., 2015b). Retinochrome is thought to be involved in the recycling of the retinal photopigment back to its original state before its interaction with r-opsins in the rhabdomes (Terakita et al., 1989). In embryos, this correlation of expression and their co-localization suggest that both retinochromes are good candidates for playing this role. By contrast in the adult, they seem to be differentially expressed. Therefore it would be interesting to know if there is a functional differentiation of these two genes and look at their expression in more diverse tissues.

### R-Opsin2 and Xenopsins

A partial sof_r-opsin2 sequence was found in our embryo transcriptomes but its expression was not detected at a significant level in any embryonic tissue studied. Thus we can hypothesized that it does not play an important role in light detection during the embryogenesis of *S. officinalis* (at least in studied tissues) even if we cannot exclude a high translation rate as the mRNA expression is not always correlated with protein synthesis. The presence of r-opsin2 was found in the brain and optic lobes of the adult and needs to be confirmed in the CNS of juveniles. This is convergent with previous knowledge as it was shown in *I. paradoxus* (Yoshida et al., 2015). A full sof_r-opsin2 sequences was retrieved from a transcriptome of adult *S. officinalis* including more nervous tissues (oesophageal ganglia and axial nerve cords): this might indicate that this gene is expressed in nervous tissues outside the CNS. It would be interesting to do a thorough investigation of the expression of r-opsin2 in more organs (including the peripheral ganglia and nerve cords) of adults *S. officinalis* in order to get some insight on its putative role.

To our knowledge, we described for the first time a duplication of a xenopsin gene in metazoans. Interestingly the C-terminal part of the protein is fully identical in both Sof_xeno1 and Sof_xeno2 suggesting a partial duplication likely coupled with an alternative splicing of the protein. We do not know if this duplication is linked to a functional differentiation of the two xenopsins. The xenopsin group is a recently described well-supported clade which includes a lot of gene formerly thought as c-opsins (Ramirez et al., 2016; Vöcking et al., 2017). But its phylogenetic position is currently under debate (Arendt, 2017) because it gathers only lophotrochozoan sequences and is found as a sister-group to cnidarian opsins. This suggests that xenopsin would have already been present in the ancestors of eumetazoans and would be lost in all major lineages except for lophotrochozoans and cnidarians. As for its role, not much is currently known. Xenopsins were found in the larval eyes of lophotrochozoans (brachiopod: Passamaneck et al., 2011 and flatworm: Rawlinson et al., 2019). They are co-expressed with a r-opsin in the photoreceptor of the eyes in two molluscs (*Leptochiton asellus*: Vöcking et al., 2017; *Limax valentianus:* Matsuo et al., 2019), leading to the conclusion that they most likely play a part in vision in these species. Our results show that no significant expression of xenopsin in early developing eyes is detected in *S. officinalis*. Thus it is unlikely that xenopsin plays a role in vision in *S. officinalis*. They most likely play a role in the optic lobes but their expression might be sporadic during development as the expression was not the same in the two stage 25 embryos. Xenopsins stay enigmatic for now and need to be further investigated.

### An Eye Specific Visual Arrestin

In lophotrochozoans, we found the co-existence of both a visual arrestin and a β-arrestin in all the coleoid cephalopods we studied and not only in the decabrachian cephalopods as previously thought (Yoshida et al., 2015). Based on our phylogenetic analyses, we cannot conclude on the origin of this duplication: before or after the cephalopod lineage appearance. This might be due to the fact that arrestins rely on their conformation in order to function correctly: the evolution rate of β-arrestins is therefore low compared to the evolution rate of visual arrestins. This explains the long branch of the molluscan visual arrestin clade and maybe also the difficulty to resolve the relationships. Our phylogenetic analysis showed that cephalopod visual arrestins are more closely related to β-arrestin than to vertebrates or arthropods visual arrestins. Therefore these three families arose independently as it was previously reported for the vertebrates and arthropods visual arrestins (Gurevich and Gurevich, 2006). Furthermore, it has been shown in terrestrial gastropod that β-arrestin was co-expressed with r-opsin in the rhabdomes and could translocate in response to light (Matsuo et al., 2017). Two β-arrestins were also identified in the retina of a bivalve (*Argopecten irradians*) and an electrophysiological study showed that they were able to deactivate the photoresponse at the rhodopsin level (Gomez et al., 2011). This suggests that this “Mollusca/Coleoidea visual arrestin” could have arisen from β-arrestin gene duplication followed by a functional specialization. Our results showed that Sof_ β-arr is expressed in different tissues whereas Sof_v-arr is eye specific in embryos and correlates with r-opsin1 expression in the developing eyes. As visual arrestins are known to interact specifically with opsin receptors in order to quench phototransduction in both vertebrates (Gurevich et al., 1995) and arthropods (Montell, 1999) we assume a similar role in cephalopods.

### Extraocular Light Detection

Our results suggest that extraocular photosensitive system in cephalopods (e.g., photosensitivity of CNS and the skin) most likely relies on r-opsin1 and sets-up latter than in the eyes. Indeed, widespread extraocular light detection using visual opsin and their putative phototransduction machinery was evidenced with immunostaining in the squid *D. pealeii* (Kingston et al., 2015b).

Previous work has shown that the skin of cephalopods is light sensitive suggesting a local role of r-opsin1 in the dynamic change in skin color. This takes place both in the chromatophores themselves and on sparse sensitive neurons in the epidermis. Moreover it has been linked to the expression of a r-opsin1 in several cephalopod species (*S. officinalis*: Mäthger et al., 2010; *O. bimaculoides*: Ramirez and Oakley, 2015) and also to the expression of the retinochrome in adult *D. pealeii* (Kingston et al., 2015a, b). Maybe due to the fact that we sampled the whole skin and not only chromatophores, we did not find r-opsin1 significantly expressed in the sub-adult and the expression of reti1 in the skin of the juvenile was barely significant. Despite that, our results seem to indicate that the “autonomous” photosensitivity of the skin would appear later than the ability to detect light in the eyes, around hatching or maybe even later, under direct environmental light.

We identified a very low expression of Sof_r-opsin1 and Sof_reti1 in the brain of a juvenile *S. officinalis*. These genes were also significantly expressed in the CNS (brain + optic lobes) of the adult as well as Sof_v-arr in the optic lobes. *In situ* data seem to indicate that Sof_r-opsin1 could be found in a few cells of the cortex of the optic lobes but this needs to be confirmed. In the literature, numerous non-image forming roles are described in a large variety of tissues [review in mammals by Leung and Montell (2017)]. As an example, in *D. melanogaster*, opsins are also known to entertain the circadian rhythm in the clock neurons of the brain, together with other photosensitive receptors such as cryptochromes (Szular et al., 2012). Thus it would be interesting to have a better localization of these receptors up to the cellular levels in order to better understand their functions.

## Conclusion

For the first time, 6 opsins receptors, 2 cryptochromes and 1 visual arrestin were identified in transcriptomes from *Sepia officinalis* embryos. The evolutionary history of these molecules is intricate with a gene duplication of an arrestin shared at least by all Coleoidea and two duplications of opsins shared by at least all Decabrachia which might have important implication of the functioning of the visual system in some cephalopods. Our results showed that there is an expression of photosensitive receptors in the developing eyes of *S. officinalis* as early as stage 23. Expressions of four of these photosensitive molecules (Sof_r-opsin1, So-reti1, Sof_reti2 and Sof_v-arr) increased significantly when the eyes are developing and starting to be functional (from stage 25 to 28), suggesting they play a role in visual phototransduction cascade. Not only the visual system seemed to be already effective before hatching but this light-detection system set up earlier in the eyes than in other tissues with photosensitive properties (i.e., CNS and skin) and most likely involved part of the same r-opsin transduction cascade. Furthermore, we showed for the first time an eye-specific expression of a cryptochrome in the eye of *S. officinalis* embryos. After hatching the expression of this Sof_CRY_6_ is also found in the CNS. This could indicate a indirect or most likely indirect role of CRY_6_ in the phototransduction cascade of *S. officinalis*. Finally, this study allows us for the first time to have quantitative data on the expression of these genes in embryos living in standard conditions thus opening the way for comparative studies in the future.

## Data Availability Statement

Data are publicly available on the NCBI platform under the accession numbers MN788446 to MN788460.

## Ethics Statement

The eggs were obtained in the Centre de Ressources Biologiques (CRB) in Roscoff (EU0413 – Station Biologique de Roscoff – Sorbonne University) taken in the wild or obtained from animals captured in the wild. The CRB-Station Biologique de Roscoff is an authorized institution, EU-0413, with the agreement number B-29-239-11 (delivered date 2014/04/30) to keep wild animals in captivity and do research and to capture animals in wild (Permit 102-2019 from the Maritime Affairs Directorate of the French Ministry of Ecology). The genitors in Roscoff were used only for eggs providing. They all die after reproduction as they usually do in the wild. The eggs developed in the lab in Paris. The eggs/embryos before hatching of cuttlefish are not under the legislation. **The juveniles** hatched from eggs obtained from the CREC, Université Caen Normandie in 2015. The CREC has all the authorization required to keep *Sepia officinalis* in the marine station (CREC- agreement A14384001) (delivered date 2014/12/24). Eggs were kept in aquarium of “Le Palais de la découverte” (EU 75-576) until hatching. Juveniles were kept under controlled conditions and killed at 1 month using MgCl2 as anesthetics. This experiment took place in 2015 and we had obtained an official notification from our local Ethical Committee Cuvier n°68. At that time, an authorization was not required because nothing was done that leads to suffering of animals and all were done to avoid pain. The subadults fished were used only for personal consumption by the fisherman. We have taken the target organs when the animals were just dead and the animals were kept by the fisherman. There is no need of a permit to fish in the sea.

## Author Contributions

MB designed and planned the experiment, performed data collection and analysis, as well as manuscript preparation and editing. AO performed bio-informatics analysis and supervised data collection in Japan, as well as manuscript editing. EC assembled the transcriptome of juveniles. LB-P and YB wrote the project, supervised the experiments, and assisted with the data interpretation and manuscript editing. All authors gave final approval for publication and agree to be held accountable for the work performed therein.

## Conflict of Interest

The authors declare that the research was conducted in the absence of any commercial or financial relationships that could be construed as a potential conflict of interest.

## Footnotes

1 www.graphpad.com

## Supplementary Material

FIGURE S1 Table of all the sequences used for phylogenetic analyses.

FIGURE S2 Full alignment used for phylogenetic analyses: opsins tree (1), cryptochromes tree (2) and arrestins tree (3).

FIGURE S3 Prediction of transmembrane helices in opsin proteins: Sof_r-opsin1 (1), Sof_r-opsin2 (2), Sof_reti1 (3), Sof_reti2 (4), Sof_xeno1 (5) and Sof_xeno2 (6).

FIGURE S4 Alignment of photosensitive molecules with emphasis on important features. 1. opsins: Alignment of the six opsins from *Sepia officinalis.* The seven transmembrane domains are indicated in gray boxes (based on *S. officinalis* rhodopsin annotation-Uniprot O16005). Important features of GPCR from the rhodopsin family are highlighted (black boxes): the two cysteines forming a dissulfide brigde for protein stability (1 and 3), the conserved lysine (4) of the predictive chromophore binding site (K296 in bovine rhodopsin and K305 in *S. officinalis* rhodopsin/r-opsin1) and the D/ERY domain (2) and NPXXY motif (5) for interaction with G-protein. 2. Cryptochromes: Alignment of cryptochrome sequences from *S. officinalis* annotated with the two conserved domains: the photolyase domain (dark gray) and the FAD-binding domain (light gray).
