# Supplementary material for: Diversity of Light Sensing Molecules and Their Expression During the Embryogenesis of the Cuttlefish (Sepia officinalis)
Source: Front Physiol. 2020 Sep 29;11:521989. doi: 10.3389/fphys.2020.521989 (PMC7553075; doi:10.3389/fphys.2020.521989)
Supplement: FIGURE S1 — Table of all the sequences used for phylogenetic analyses. [file Table_2.DOCX]

**Supplementary data : S2 to S4**

Table of content

[**S2: Full alignments used for phylogenetic analyses** 1](#_Toc43463027)

[**1.** **Opsins tree** 1](#_Toc43463028)

[**2.** **Cryptochromes tree** 24](#_Toc43463029)

[**3.** **Arrestins tree** 33](#_Toc43463030)

[**S3: Prediction of transmembrane helices in opsin proteins (TMHMM output)** 40](#_Toc43463031)

[**1.** **Sof_ropsin1** 40](#_Toc43463032)

[**2.** **Sof_ropsin2** 41](#_Toc43463033)

[**3.** **Sof_reti1** 42](#_Toc43463034)

[**4.** **Sof_reti2** 43](#_Toc43463035)

[**5.** **Sof_xeno1** 44](#_Toc43463036)

[**6.** **Sof_xeno2** 45](#_Toc43463037)

[**S4: Alignment of photosensitive molecules with emphasis on important features** 46](#_Toc43463038)

[**1.** **Opsins** 46](#_Toc43463039)

[**2.** **Cryptochromes** 47](#_Toc43463040)

**S2: Full alignments used for phylogenetic analyses**

1. **Opsins tree**

>Sof_r-opsin1_MN788446

QVPDAVYYSLGIFIGICGIIGCTGNGIVIYLFTKTKSLQT-PANMFIINLAFSDFTFSLVNGFPLMTISCFIKKWVFGMAACKVYGFIGGIFGLMSIMTMSMISIDRYNVIG--RPMAAS--KKMSH-RRAFLMIIFVWMWSTLWSIGPI--FG-WGAYVLEGVLCNCSFDYITRDS--ATRSNIVCMYIFAFCFPILIIFFCYFNIVMAVSNHEKEMAGASAEMKLAKISIVIVTQFLLSWSPYAVVALLAQFGPWVTPYAAQLPVMFAKASAIHNPLIYSVSHPKFREAIAENFPWIISCC-QFDEKEV

>Doryteuthis_ropsin1_comp42272_c1_seq1

QVPAAVYYSLGIFIGICGIIGCVGNGIVIYLFTKTKSLQT-PANMFIINLAFSDFTFSLVNGFPLMTISCFLKYWVFGNAACKVYGLIGGIFGLMSIMTMTMISIDRYNVIG--RPMSAS--KKMSH-RKAFIMIIFVWIWSTTWAIGPI--FG-WGAYSLEGVLCNCSFDYISRDS--STRSNIVCMYLFAFMCPIIVIFFCYFNIVMSVSNHEKEMAGASAEMKLAKISIVIVTQFLLSWSPYAIVALLAQFGPWVTPYAAQLPVMFAKASAIHNPMIYSVSHPKFREAIASNFPWILTCC-QFDEKEI

>Euprymna_ropsin1_EU344773.1

QVPDAVYYGLGIFIGICGIIGCGGNGIVIYLFTKTKSLQT-PANMFIINLAFSDFTFSLVNGFPLMTISCFLKKWVFGHAGCKVYGFIGGIFGLMSIMTMAMISIDRYNVIG--RPMAAS--KHMSH-RRAFIMIIFVWLWSVLWAIGPI--FG-WGAYTLEGVLCNCSFDYITRDY--ATRSNIICMYVFAFCFPIIIILFCYFNIVMSVSNHEKEMAGASAEMKLAKISIVIVTQFLLSWSPYAIVALLAQFGPWVTPYAAQLPVMFAKASAIHNPMIYSVSHPKFREAISNTFPWILTCC-TFDEKET

>Obimaculoides_ropsin1_A0A0L8G9D3

PIPDAVYYSVGIFIGVVGIIGIFGNGVVIYLFSKTKSLQT-PANMFIINLAMSDLSFSAINGFPLKTISAFMKKWIFGKVACQLYGLLGGIFGFMSINTMAMISIDRYNVIG--RPMAAS--KKMSH-RRAFLMIIFVWIWSIVWSVGPV--FN-WGAYVPEGILTSCSFDYLSTDS--STRSFILCMYFCGFMLPIIIIAFCYFNIVMSVSNHEKEMAGQSAEMKLAKISMVIITQFMLSWSPYAIIALLAQFGPWVTPYAAELPVLFAKASAIHNPIVYSVSHPKFREAIQNTFPWLLTCC-QFNEKEC

>Enteroctopus_ropsin1P09241

PIPDAVYYSVGIFIGVVGIIGILGNGVVIYLFSKTKSLQT-PANMFIINLAMSDLSFSAINGFPLKTISAFMKKWIFGKVACQLYGLLGGIFGFMSINTMAMISIDRYNVIG--RPMAAS--KKMSH-RRAFLMIIFVWMWSIVWSVGPV--FN-WGAYVPEGILTSCSFDYLSTDP--STRSFILCMYFCGFMLPIIIIAFCYFNIVMSVSNHEKEMAGASAEMKLAKISMVIITQFMLSWSPYAIIALLAQFGPWVTPYAAELPVLFAKASAIHNPIVYSVSHPKFREAIQTTFPWLLTCC-QFDEKEC

>Ovulgaris_ropsin1_A0A0R6YPZ7

PIPDAIYYSVGIFIGVVGIIGIFGNGVVIYLFSKTKSLQT-PANMFIINLAMSDLSFSAINGFPLKTISAFMKKWIFGKVACQLYGLLGGIFGFMSINTMAMISIDRYNVIG--RPMAAS--KKMSH-RRAFLMIIFVWIWSIVWAVGPV--FN-WGAYVPEGILTSCSFDYLSTDS--NTRSFILCMYFMGFMLPVVIIAFCYFNIVMSVSNHEKEMAGQSAEMKLAKISMVIITQFMLSWSPYAVIALLAQFGPWVTPYAAELPVLFAKASAIHNPIVYSVSHPKFREAIQNTFPWLLTCC-QFNEKEC

>Nautilus_ropsin_A0A0H5B8K8

GVPDAAHYIVGIFITVVGICGVMGNGVVIYIFSRTKSLRT-PANMFIINLALSDLTFSAVNGFPLLSISSFQKKWIFGQTACELYGLAGGIFGLMSINTMAMISIDRYNVIA--RPMAVS--KRMSH-KKAFIMIISVWIWAAVWTLPPL--FG-WGAYIPEGFQTSCTFDYLTRNN--YFRSYVLCLYLFGFITPVTIIAICYFFIFKAVADHEKEMSEQRAEMKIAKISMIIITQFLLSWTPYAVVAMLGQFGPWVTPYASEVPVMFAKASAMHNPIVYALSHPKFREAINERFPFLISCC-AFDEKET

>Idiosepius_ropsin2_A0A0H5ARC1

QVPDAVYYCLGIFIGICGIIGCGGNGIVIYLFTKTKSLQT-PANMFIINLAFSDFTFSLVNGFPLMTISCFLKKWIFGMAACKVYGFIGGIFGFMSIMTMSMISIDRYNVIG--RPMAAS--KKMNH-RRAFLMIIFVWLWSTMWAIGPI--FG-WGAYALEGVLCNCSFDYISRDP--STRSNIICMYILGFMLPIIIIFFCYFNIVMSVSNHEKEMAGANAEMKLAKISIVIVTQFMLSWSPYAVVALLAQFGPWVTPYAAQLPVMFAKASAIHNPMIYSVSHPKFREAIAAQFPWILTCC-QYDEKEI

>Obimaculoides_ropsin2_XM_014916694.1

--------------------------IVIVLNPGYKRLHT-SPNIILASLAFSDTIMSSI-DMCFVSVSSFSGRWVFGVVGCKAYGFIVSVVSILSICCLTLIAIDRYVVIV--KGWVNY--RSSRK-KSAFVSILIAWIYTLAWCICPL--FG-WGDYILEGIGSSCTFDYITRTP--QNRSYMLCLNIFVFFLPIVIILICYINIIIAMYKHKKDMNKMSVELKTVRSLAAAIILFCAGWMPYSVLSLIAQFGNIITRASTGYPGIAAKCITCANPFFYALSNPIFLKRLKIFIFCGRVSSFQTSKSWI

>Ovulgaris_ropsin2_XM_029783631.1

--------------------------------------------------------MSSI-DMCFVSVSSFSGRWVFGVVGCKAYGFIVSVVSILSICCLTLIAIDRYIVIV--KGWMNY--GSSRK-KSAYVSILIAWIYTLAWCICPL--FG-WGDYILEGIGSSCTFDYITRTP--QNRSYMLCLNIFVFFLPIVIILICYTNIIIAMYKHKKDMNKMSVELKTVRSLAAAIILFCAGWMPYSVLSLIAQFGNIITRASTGYPGIAAKCITCANPFFYALSNPIFFKRLKIFVSCGRVSSFQTSKSWV

>Euprymna_PutativeR-opsin2_SRIE01056463.1

EVPSDYHIGAGVIMCISSVISIFGNGMVLLVICKFKRMHS-SANILVGNLAFADIIMSGM-DAAFVTTSSFYRQWIFGNLG-------------------------RYVVIV--KCWVNY--RRSRK-KTAVWFLIIAWVYAIFWATCPL--FG-WGAFMLEGFGSSCTFDYLTRSL--RNTSYVICLILFAFFIPISIITVCYINIIRAMYKHRKDMKKLNVELKTARALAIAIVLFCAAWAPYAIMSLIAQFGDLISRLATGIPGIAAKCVTCMNPFLYAISNPLFLRKIKEVLCLRQTRQ-QEIEESS

>Sof_r-opsin2_ comp254721_c1_seq2

LIPSDYHIGTGVVMCISLFMSIFGNSMVLLVICKYKRMHS-SANIMVGNLALADIVMSSL-DAGFVAVSSFHGRWIFGNFGCKAYAYTVSMMSILSISTLTLIAIDRYVVIV--KCWVNY--RRSRK-KTTIWFLSIAWFYAILWATCPL--LG-WGAFMLEGFGTSCTFDYLTRSL--NNTSYVICLNIFAFFIPVSIISVCYVNIIRSMYKHKKDMKKLNVELKTARALAIAIVLFCAGWAPYAIMSLIAQFGDLISRLATGIPGIAAKCVTCMNPFLYAISNPIFLRKIKEILCCRRTQP-QEREDST

>Idiosepius_ropsin2_A0A0H5ANT7

---------------------------------------------------------------------------------------------------------------------------------------------------------------MLEGMGTSCTFDYLTRSP--NNTSYVICLNIFAFFVPVSIISVCYVNIIRAMYKHKKDMKKLNVELKTARALAIAIVLFCAGWAPYAIMSLIAQFGDVISRFSTGIPGIAAKCMTCMNPFLYAISNPLFFRKIKEMLCFRRTQP-QEREDSS

>Platynereis_ropsin2_KC810972.1

AQPYSTHLAIGITMSIMGFVAITTNTFVMFVFLRFRSLRT-PGNLLVVNLAFSDSMLALL-GFPLYAASSFIGHWSFGKIGCNFYGFSGASFGLMSINTMAAIAVDRYLVIV--RPYMSI--RRISY-SQAYIMLGIVWVNAIGWSVPPL--VG-WSRYILEGLGTTCTFDYLSRDP--VTRTYLVSLYVGGFVIPVLLIVYCYTYIFLRVRQHEKAFATRRADVKTARTGFLAVIVFCIAWSPYAVMALVGQFGDLLTPIVSAIPGILAKSSTIYNPMIFAVSHPRFRRKLRYLYFAT-----ANTDDTD

>Lottia_hypothetical_XP_009065095.1

PVSAAWHNFIGIFITFVGITGVIGNFVVIYTFSRTKSLRT-ASNMFVVNLALSDLTFSAVNGFPLFSLSSFSHKWIFGRVACELYGLIGGIFGLMSINTMAMISIDRYLVIT--SPFTAM--RNMTH-KRAFLMIVGVWIWSILWAIPPI--FG-WGAYIPEGFQTSCTFDYLTRGD--NRRSYIMCLYICGFVVPLGVIIFCYVFIIKSVMNHEKEMEKAKAEIKIAKVSMTIILLYLMSWTPYAIVALIAQWGPVVTPYVSEIPVLFAKASAMHNPVIYALSHPKFRDAVSKLMPWFLCCC-GLTDAE-

>Lottia_hypothetical_XP_009062294.1

PIDEFASIIAAISMIVVFFIGTAGNSTVIYLFARHKNLQT-SANMYVANLASADLIMCLF-NFPILIWASLKGQWQLGNIGCQIYGVIGALTGFMSINTLTAMAVDRYQVIN--TNNSFM--QKTSK-GRHAFIICGIWVYSVSWALVPV--AG-WGRYVLNGGRTSCCFDYLTRDS--INLAFIVCLFIFCFTIQLFVILYCYTSMLVFICRHERAFKKIKLELKVARLVFIIVSAFCLSWMPFAVVTLIGAFGDYITPTVSIVPGLLAKISTAINPMLYAISHPKFQSKLRASCPCPLFKA-GKAGDLT

>Biomphalaria_rhodopsin-like_XP_013084162.1

LISPAWHYAMGIFITFVSIFGSLGNLLVMYIFGTTKSLRT-PSNMFIVNLSMSDFIFSIIMGFPLMTISCFNRRWIFGKVACELYGLVGGIFGLMSINTLAVIAFDRYSVIA--RPIKAS--RSLSF-RKAFFMLVFVWCWSTTWTIPPL--FG-WGAYIPE------------------------------------------------------EMSAQKAEIKTARIAMTIVITYLMSWVPYATIALIAQFGPLVTPYVSELPVMFAKASAMHNPMIYALSHPRFREALDKKFPWLLCCC-GVSEKEK

>Pomacea_rhodopsinGQ-coupled-like_XP_025104434.1

QLDPALRVIMGTIMLVVGIGGAVGNCTILYFLFRYKKLRK-SANVYVVNLASVDVLMCIA-NFPLFVYSCFEGTWVTGYTTCQVYSFLGGVTGFVSINTLTAMACDRYSAIF--RRHGVARSDSATC-SRSLGLLISVWIYSIFWSVPPF--FG-WGQFALDGGRVSCCFDYLTRSA--SNVSYIVAIFIFCFAIQMAVIIFCYVSMVAAFVQRKGDLKRRRLELRVTRLVCCITMSFCFSWLPFAVVALIGTFGNQITPMVSTLPGMLAKTSTVVNPLIYAIGHPHYRRVLQKNWRCG-----KTYKRRA

>Pomacea_rhodopsinGQ-coupled-like_XP_025112895.1

PLPYAMHVCIGVVVFFVAAFAIVGNVLVLWSIVRFRSLRT-SSNLFIISLAVADIIMSVI-DFPLFAVSSFLGYWSFGYNMCQMYTISTGAAGLVNINTLAAISYDRYTVVV--RRVLPV--HHVSR-SMTRLVIAVIWISSVLWMMFPM--MG-WGHFTLEGTGTSCTFDYLDRSA--ANRAYVTTLTLANFVVPLIVIVFSYVRIFLSVSAVRRGLRKLHTELKMAVTTLVIVGVFCVSWTPYVIVATIALFGDLVTPMTSAVPCLLAKVATVSNPPLYSLGHPKFRKKIRLLVMSA------------

>Pomacea_rhodopsinGQ-coupled-like_XP_025099879.1

GVSQLQHTAIGVFLVGFTVTATVSNILVVSTCLGYRSLRT-TSNLLVVNLAAGDLVMCVV-DFPLFAVASFFNFWPFGMNVCQVYSLITAMAGLVTINTLAVISADRYRAIV--NRLCQHH-QRTSR-PTTLLIVLFIWGWSALWAVAPM--LG-WGRYVLDGVGTTCTFDFLTRTA--NNISFVMAMMMGNFVIPLGVITFSYLHIWRAVLDAKRRLLRIRSETRTACTILTLVLAFVVAWFPYLVVCMIGLFGDQVTVTASVVSSLVAKTSTVSNPVLYCIIHPKVRRKLHVTLLRLLWST-SPRRSFH

>Aplysia_rhodopsinGQ-coupled-like_XP_012943629.1

AVPYTFHLVVGIFITIVGILAVVGNVLVLCTFARHSSLRT-SSNLLVVNLTVADLVMSSL-DFPILAISSYKGCWVMGFLGCQVYGVSSGVAGLVTINTLAAISVDRFVVVV--HRLSPM--HQMGK-STTGVIIIAIWALSVIWAVLPI--TG-VSSYRLEGMGTSCTFDYASRTS--SNRWFFIALVIFNFFIPLALIIFSYWRIYASVRAVKRELFEVQAEIKTAITALVIISIFCLAWTPYVIIAFVGLYGPAIDPLVSMFPNILAKISTVSNPILYSIGHPEVRKKMKKLFL-------PGQQDSS

>Mizuhopecten_RhodopsinGQ-coupled_OWF40989.1

PVSDFWFYFVGIYIGIVGITGVVGNILVIWMFTTNKKLKT-PSNMLIVNLALSDLTFSAVNGFPLKSISAFSKKWVFGMVACELYGLIGGIFGFMSISTLAAISIDRFICIT--KPLQAA--RIMTR-KKAFIMIVVVWTWSVFWSIPPL--FG-FGAYIPEGFQTSCTFDYITKST--SNRIFIIGMYVFGFLMPCLIIIGCYIQILKAIKAHGKEMKKAKAEMKIAKIALMLISLFILSWSPYATIALIAQFGSFVTPLMSELPVLLAKTSAMHNPLVYALSHPRFRAALAEKAPCFMVCC-PPEKTPT

>Mizuhopecten_rhodopsinGQ-coupled-like_XP_021373217.1

PVSDNWHYFIGLFITVVGISGVVGNIVVIWMFSSTKTLKS-PSNMLITNLALSDLTFSAVNGFPLLTISAFNKRWVFGDAACEFYGLIGGIFGLMSINTLAMISIDRYICIT--KPLQAA--RLMTR-KKAFFMIVIVWSWAVGWSLLPL--FG-LGAYIPEGFQTSCTFDYLTKTT--LNRIYIIGMYLFAFALPLVIIIGCYIAILKAIRKHAKEMEKSKTEIKIAKIAMMLISLFILSWSPYATIALMAQFGDFVTPFMSELPVMLAKASAMHNPIVYALSHPKFREALMKKAPCLLSCC-APSEKPK

>Crassostrea_rhodopsinGQ-coupled_XP_011447068.1

LVSDNWHYAIGVFISIVGIIGIFGNATVIYIFSTTKNLKT-PSNMFIVNLALSDMIFSLVMGFPLLTISAFNKKWIWGNTACELYGLVGGIFGLMSITTLSAISVDRYYAIA--HPLRAA--RNMTR-KKAFMMICIVWVWSLCASLPPL--FG-WGRYVAEGFQTSCTFDYLTTTP--NNRTYIFFLYLFGFAAPLLVIALSYILIIRALKKHERKMEKTKAEVKVAKVAIIIVFFYMLSWSPYATVALIGQFGPWVTPFLSELPVMLAKASAMHNPIVYALSHPKFREALYKRAPWIFCCC-EPPSKAT

>Argopecten_opsinGq1_ALO02514.1

PVTPEWHYVIGIYITIVGLLGITGNSVVIYIFSHTKSLRS-PSNLLVVNLAVSDLIFSAFNGFPLLTVSSFHQKWVFGSVTCQLYGFIGGVFGLMSINTLTAISIDRYVVIT--RPLQAS--QTMTR-SKVHLMIFFVWLFSILLSVPPF--FG-WGAYIPEGFQTSCTFDYLTKTP--RTRAYIVVLYLFGFALPLFIIGVCYVMIIHGVRRHDQKMKRARSEMRISKIAMAVTCLFTISWSPYAIVALIGQFGPWITPLVSELPMMLAKTSAMHNPIIYALSHPKFRKALYRKAPWVFCCC-KPEDKPD

>Argopecten_opsinGq3_ALO02516.1

VVSDSWHFFVGLFITVVGISGVTGNIVVIWIFSSVKTLKT-PSNLLIVNLALSDLTFSAVNGFPLFTISAFNKRWVFGDAACEFYGLIGGIFGLMSINTLAMISVDRFICIT--KPLQAA--RIMTR-KKAFLMIVVVWSWAIGWSILPL--FG-LGAYIPEGFQTSCTFDYLTKTT--SNRIYIIGMYVFAFALPLLIIIGCYVGILKAIRKHAKEMEKSKTEIKIAKIAMMLISLFILSWSPYATIALMAQFGDFVTPYMSELPVLLAKASAMHNPIVYALSHPKFREALMKKAPCFLSCC-MPSDKPQ

>Argopecten_opsinGq2_ALO02515.1

PVPDTWHYFIGLFITVVGISGVVGNIVVISVFTSTKTLKS-PSNMLIVNLALSDLTFSAVNGFPLLTVSAFNTKWVFGDVACQFYGFIGGIFGLMSINTLAMISIDRCICIT--RPLQAA--RIMTR-KTAFIMIVVVWVWAVGWSLLPF--FG-LGAYIPEGFQTSCTFDYLTKSL--SNRIYIVGMYVFAFALPLLLIVGSYVMIIGAIRKHAREMEKTKAEIKITKIAMTLISLFILSWSPYATIALMAQFGDFVTPLMSELPVMLAKASAMHNPIVYALSHPKFRAALNEKAPCFLNCC-RPKPKPP

>Argopecten_opsinGq4_ALO02517.1

PVSDSWHIFDGLFIGVVGITGVIGNILVIWMFTTCKKLKT-PSNMLIVNLALSDLTFSAINGFPLKSISAFSKKWVFGMVACELYGLTGGIFGFMSIGTLAAISIDRFICIT--KPLQAA--RIMTR-KKAFIMIVIVWIWSTLWSIPPL--FG-LGSYIPEGFQTSCTFDYITKST--SNRYFILGLYVFGFLTPLTIIIGCYIIILRAIKAHGREMKKAKAEMKIAKIALMLISLFILSWCPYATIALIAQFGSFVTPLMAELPVLLAKTSAMHNPLVYALSHPRFRAALAEKAPCFLRCC-PVEPAQT

>Mizuhopecten_rhodopsinGQ-coupled_XP_021357501.1

PVTEEWHYIIGVYITIVGLLGIMGNTTVVYIFSNTKSLRS-PSNLFVVNLAVSDLIFSAVNGFPLLTVSSFHQKWIFGSLFCQLYGFVGGVFGLMSINTLTAISIDRYIVIT--KPLQAS--QTMTR-RKVHLMIVIVWVLSILLSIPPF--FG-WGAYIPEGFQTSCTFDYLTKTA--RTRTYIVVLYLFGFLIPLIIIGVCYVLIIRGVRRHDQKMKRARSELRISKIAMTVTCLFIISWSPYAIIALIAQFGPWITPLVSELPMMLAKSSSMHNPVVYALSHPKFRKALYQRVPWLFCCC-KPKEKAD

>Mizuhopecten_rhodopsinGQ-coupled_OWF48482.1

PVTEEWHYIIGVYITIVGLLGIMGNTTVVYIFSNTKSLRS-PSNLFVVNLAVSDLIFSAVNGFPLLTVSSFHQKWIFGSLFCQLYGFVGGVFGLMSINTLTAISIDRYIVIT--KPLQAS--QTMTR-RKVHLMIVIVWVLSILLSIPPF--FG-WGAYIPEGFQTSCTFDYLTKTA--RTRTYIVVLYLFGFLIPLIIIGVCYVLIIRGVRRHDQKMKRARSELRISKIAMTVTCLFIISWSPYAIIALIAQFGPWITPLVSELPMMLAKSSSMHNPVVYALSHPKFRKALYQRVPWLFCCC-KPKEKAD

>Mizuhopecten_Rhodopsin_O15973.1

PVTEEWHYIIGVYITIVGLLGIMGNTTVVYIFSNTKSLRS-PSNLFVVNLAVSDLIFSAVNGFPLLTVSSFHQKWIFGSLFCQLYGFVGGVFGLMSINTLTAISIDRYVVIT--KPLQAS--QTMTR-RKVHLMIVIVWVLSILLSIPPF--FG-WGAYIPEGFQTSCTFDYLTKTA--RTRTYIVVLYLFGFLIPLIIIGVCYVLIIRGVRRHDQKMKRARSELRISKIAMTVTCLFIISWSPYAIIALIAQFGPWITPLVSELPMMLAKSSSMHNPVVYALSHPKFRKALYQRVPWLFCCC-KPKEKAD

>Acanthopleura_opsinPartial_APF30601.1

PVPDHWHYIIGVYITLVGITGVFGNFLVIWIFSRTKSLRT-PANMFVVNLAISDLTFSAINGFPLLSFSAFYKRWIFGKAACELYGLVGGIFGLMSINTMAMIAIDRYLVIA--RPLSVM--RHMSH-KRAFFMLMLVWIWSILWAIPPI--FG-WGAYIPEGFQTSCSFDYLTRTD--YFRSYIFCLYICGFVVPVLIIFFCYVLIVKAVADHEKEMQDQKTEIKTAKIAFYTITLFLLSWTPYAVVALIGEFGPYVTPYASEIPVMFAKTSAMYNPIVYALSHPKFREVLNKKLPWLMVCC-KPKEKPS

>Leptochiton_r-opsin_AMB26726.1

----------------------------------------------------------------------------MGKAACELYGLAGGIFGLMSINTMTMIAIDRFLVIA--RPISVM--RKMGH-KRAFFMIMLIWVWSLLWATPPI--FG-WGAYVPEGFLTSCSYDYLTRTD--HNRSFIICMYSFDFVLPVSIIFYCYTKIVKAVADHEKEMDDQRTELKTAKIAFITIMLFLLSWTPYAVVALIGEFGPYVTPYAAEIPVMFAKASAMYNPIVYSLSHPKFRAVLNEKFPWLMICC-KPKKKVR

>Platynereis_RhabdomericOpsin3_R4RY94

ELPDIIHWVIGVYIAIVGMIGVIGNFLVIFFFSTTRSLRT-PSNLFVINLAISDLGFSAVNGFPLMTISSFMRKWYFGNIACTLYGFLGGIFGFNSIGSLMFISLDRYYVIA--KPLEAM--RKATK-KRAFLQIIVIWIWALIWSGPPL--IG-FGSYIPEGLQTSCSFDYLTRSP--TNIAFNMGLFGMGFCFPLLIIIACYVQIIAAVSRQAREMRKKQQEIQLAKVAAGTISLFCISWIPYALVAQFGIWGLFVNPITCVVPVLFAKASAMWNPILYALSHPRFRAVLDDKMPWLLCCK-GKNMDKG

>Platynereis_RhabdomericOpsin_Q8T359

TIPDSWHYAVAAWMTFFGILGVSGNLLVVWTFLKTKSLRT-APNMLLVNLAIGDMAFSAINGFPLLTISSINKRWVWGKLWRELYAFVGGIFGLMSINTLAWIAIDRFYVIT--NPLGAA--QTMTK-KRAFIILTIIWANASLWALAPF--FG-WGAYIPEGFQTSCTYDYLTQDM--NNYTYVLGMYLFGFIFPVAIIFFCYLGIVRAIFAHHAEMADKKSEIQIAKVAAMTIGTFMLSWTPYAVVGVFGMIKPFIHPLLAEIPVMMAKASARYNPIIYALSHPKFRAEIDKHFPWLLCCC-KPKPKAQ

>Platynereis_RhabdomericOpsin2_A0A0K0YBF1

NVPDSVHYILGIYITFVGFAGVIGNAIVIFVFTATKSLRT-PSNMFIVNLAMSDLGFSLVNGFPLMSVSSFMRKWYFGRVGCILYGTLSGVFGLTSINTLALIAFDRFYVIQ--FPLRAI--RTVTR-TRSFVQICLVWIWATFWSCPPL--FG-WGRYIPEGLQTSCGFDFLSQDP--LNRAFNYCIFSCGFVLPVTFAICSYCGILATVSMQAKHMKEKKQQIRLAKIAAGTISLFIISWMPYALLVILSTSGYIMTPYVCQIPSVFAKASAIWNPFVYSISHPKYRQALQERFPWLLCNK-KDTDDVI

>Platynereis_RhabdomericOpsin5_A0A0K0YBF4

PPPHEIHITIGFAMATIGVLAVAGNTFVIFVFLRFRSLRT-PGNLLMINLAVSDLLMAVT-GFPLYSISSFYGRWVLPDAVCLFYGACGATFGLLSINSLAAIAVDRYLVIA--HSYAVT--KRTNR-RQAIVMIVLSWINSLCWAIPPL--LG-WNRYLLEGFGTTCTFDYLSRTK--SDRLFVMLMFCCGFCLPLLLIIGSYAYIYSVVHRHERMFATQRTEMKTARTVILAVLFYCISWVPYATIALIGIYGNLLTPLVTAVPGILAKMSTIYNPLLYTFSHPRFHKKVMLLLFKRSMVLDKNTSNMD

>Platynereis_RhabdomericOpsin4_R4S0M6

AQPYSTHLAIGITMSIMGFVAITTNTFVMFVFLRFRSLRT-PGNLLVVNLAFSDSMLALL-GFPLYAASSFIGHWSFGKIGCNFYGFSGASFGLMSINTMAAIAVDRYLVIV--RPYMSI--RRISY-SQAYIMLGIVWVNAIGWSVPPL--VG-WSRYILEGLGTTCTFDYLSRDP--VTRTYLVSLYVGGFVIPVLLIVYCYTYIFLRVRQHEKAFATRRADVKTARTGFLAVIVFCIAWSPYAVMALVGQFGDLLTPIVSAIPGILAKSSTIYNPMIFAVSHPRFRRKLRYLYFAT-----ANTDDTD

>Apis_OpsinBlue-sensitive_P90680

APGKHFHIGLAIIYSMLLIMSLVGNCCVIWIFSTSKSLRT-PSNMFIVSLAIFDIIMAF--EMPMLVISSFMERMIGWEIGCDVYSVFGSISGMGQAMTNAAIAFDRYRTIS--CPIDG----RLNS-KQAAVIIAFTWFWVTPFTVLPL--LKVWGRYTTEGFLTTCSFDFLTDDE--DTKVFVTCIFIWAYVIPLIFIILFYSRLLSSIRNHEKMLKERSAEVRIAKVAFTIFFLFLLAWTPYATVALIGVYGNLLTPVSTMLPAVFAKTVSCIDPWIYAINHPRYRQELQKRCKWMGIHEPETTSDAT

>Apis_OpsinUltraviolet-sensitive_O61303_SV_1

EPNPSLHYLLALLYILFTFLALLGNGLVIWIFCAAKSLRT-PSNMFVVNLAICDFFMMI--KTPIFIYNSFNTGFALGNLGCQIFAVIGSLTGIGAAITNAAIAYDRYSTIA--RPLDG----KLSR-GQVILFIVLIWTYTIPWALMPV--MGVWGRFVPEGFLTSCSFDYLTDTN--EIRIFVATIFTFSYCIPMILIIYYYSQIVSHVVNHEKALSSQSAEIRIAKAAITICFLYVLSWTPYGVMSMIGAFGNLLTPGVTMIPACTCKAVACLDPYVYAISHPKYRLELQKRLPWLELQE-KPISDST

>Apis_RhodopsinLong-wavelenth_Q17053

PLNPMWHGILGFVIGMLGFVSAMGNGMVVYIFLSTKSLRT-PSNLFVINLAISNFLMMFC-MSPPMVINCYYETWVLGPLFCQIYAMLGSLFGCGSIWTMTMIAFDRYNVIV--KGLSG---KPLSI-NGALIRIIAIWLFSLGWTIAPM--FG-WNRYVPEGNMTACGTDYFNRGL--LSASYLVCYGIWVYFVPLFLIIYSYWFIIQAVAAHEKNMQNTSAECKLAKVALMTISLWFMAWTPYLVINFSGIFNLKISPLFTIWGSLFAKANAVYNPIVYGISHPKYRAALFAKFPSLACAA-EPSSDAV

>Lingula_rhodopsinGQ-coupled_like_XP_013407359.1

PPSTTIILVSGIILLVFAVISILGNAFVIGIFCSSPSLRT-PANMLILNLSWSDLMMST--AVPMVTVAALEGKWVFGKVGCDIHAMVVGIFGLMSMNTIAAIAFERYKVIV--RSCP----TKGSR-RQVAVSVVVTWLYSILWTLPPI--FG-WGQYILEGTGTSCTFDFLTQTL--ENRLFVLALFIGGFVVPFLVILVCYCNVFYYVHASNKLLKALKSEVKVVKMTMVILLLFCLSWIPYSVVALIGTFGDQISPLTAGIPVIFAKSSAMYNPVIYTFYHQGFRRELRRRRICGRLSA-RFFNLSS

>Xenopus_RetinalGPCR_Q28EK0

GFTETEVFAIGTTLLVEALLGLLLNGLTLLSFYKIRELRT-PSNLFIISLAVADTGLCLN-A-FVAAFSSFLRYWPYGSEGCQIHGFQGFVAALSSIGSCAAIAWDRYHQYC--TR------SKLHW-STAVSVVFFIWGFSAFWSAMPL--FG-WGEYDYEPLRTCCTLDYSKGDR--NYISYLFTMAFFEFLVPLFILMTAYQSIYQKMKKSGQIR------------TSMPVKSLVFCWGPYCLLCFYAVIQDILSPKLRMIPALLAKTSPAVNAYVYGLGNENYRGGIWQYLTGQKLEK-AETDNKT

>Crassostrea_Rhodopsin_K1QMS3

-----------------------------------RSLRI-SSNLLVLNLAITDTFLALG-NLPWLAISSFRGVWIFGYIGCQVYGFVGAMSGFISINTLAMIAIERFFVIVIREPYRH---IRTSN-KTVLISIMFIWIYSFVWAICPL--IG-WGSIILEGSMTSCTFDFFSRDV--NTKSYVASILVFCFTVQLFLIICSYVRIYLKVLQHEKEIKFRSVHVKTAKISLVIISIFCLSWTPYAVVAIIGNFGDVITPLASTIPGVFAKLSTVLNPMIYALLHPKFRNKLPFRKKVL------------

>Ambigolimax_rhodopsin_BBA21101.1

LITPAWHYAMGIFITFVSIFGSMGNMLVMYIFGTTKTLRT-PSNIFVLNLSMSDLIFSIINGFPLMTISCFNRRWIFGKVACELYGLVSGIFGLMSINTLAAIAFDRYNVIA--RPMKAS--RGMSY-RRAFMMLVFVWCWSATWTIPPL--FG-WGAYIPEGFQTSCTFDYLTRGN--YFRSYILCLYICGFATPLLIIIFCYVQIYGAVARHEKEMNARRTEIKTAKVSLTIVLAFLLSWTPYATVALIAQFGPLVTPYVSEIPVMFAKASAMHNPLIYALSHPRFREAVDKRFPWLLCCC-GMTAKEK

>Ambigolimax_xenopsin_BBH84660.1

TLSGTGFIFIGCMLSVTWSFGTFFNGLCLYIFATTAGLRS-PTNIFIIALNLCDFLMCFV-GTPAALTSAWARRWLWGRAGCKIEGFLVYFLGMTSMYLLTAIAVIRYIAIS--KPLLV---RRTST-AVAVVSCVCCSLLGFLWAVLPA--FG-WNEFGLEGAGISCSVIWHSTDP--SCMSYIWTVFFTCLVIPVGIMLFSYGGVLVTLRNLNRNSKNLAIEKKMLRTSVLIVLSFICFWTPYAAVSFITAFFGHISAIFASIPPVVAKCQGLMDPVIYVASNKQVRTEVLKMLPCRVLQE-QLLKRIS

>Ambigolimax_opsin5A_BBH84661.1

TMGNTWDIVIALVVLCVIITGFTLNSLVLWILIRLKATQPHNSDYYIMALAASDLLVVLS-CYPLTFMSTISHRWLFGDVGCAINGFFGFLFSLIDIGALTMMSLHRLLSVK--MPHCA---FVRSA-GYTWVALVGVVLYALVWTVSPL--LG-WGRFAPEPFGTSCTIDWKN-----PSRSYVTSVFTGVLTIPMLVMLMCYSSIVFHMRATSAKFKREKRQNRLVRIAVLMCTCFLVIWLPYSVLSICFSYVPSVHPVHSAIPTVLAKASHVVNPLICFATNKSYRRLVRRSFLCF-----ETSKSCQ

>Ambigolimax_opsin5B_BBH84662.1

TLSTTEDIVVGSYLIVVECMAILLNGSILGLSAWKRRTLK-VSDWYVVWLAMSDLGHPVV-GYPMTITSSFKYAWVYGDIGCQWNAFTGFFFGVNSMITLAAMSLSRLFIIT--QPDFA---RQHSN-KMAVCLIGFTLLYALFWAACPF--LG-WGQYGPEPYKTSCTLVWDQ-----PDQTFVTAAFIGCLAIPAATMCYSYYRILHPAIRTRHERVWEKKEMRLLKITIIMCASFMVCWTPYSVVAMLKAYSSHLSPSLSVLPALAAKTSHIIDPVIYCGMNRNFNRIIPELCR--RGQE-VIIKDHS

>Danio_RetinalGPCR_Q567Y2

GFSEFDVFSLGSCLLVEGLLGFFLNAVTVIAFLKIRELRT-PSNFLVFSLAMADMGISTN-A-TVAAFSSFLRYWPYGSDGCQTHGFQGFMTALASIHFIAAIAWDRYHQYC--TR------TKLQW-SSAITLVLFTWLFTAFWAAMPL--FG-WGEYDYEPLRTCCTLDYSKGDR--NYVSYLIPMSIFNMGIQVFVVLSSYQSIDKKFKKTGQAK------------CGTPLKTMLFCWGPYGILAFYAAVENLVSPKLRMIAPILAKTSPTFNVFVYALGNENYRGGIWQLLTGQKIES-PAIENKS

>Danio_RetinalPigmentEpitheliumGPCR_Q6DH22

GFTDFDMFAFGSALLVGGLLGFFLNAISVLAFLRVREMQT-PNNFFIFNLAVADLSLNIN-G-LVAAYACYLRHWPFGSEGCQLHAFQGMVSILAAISFLGAVAWDRYHQYC--TK------QKMFW-STSITISCLIWILAVFWAAMPLPAIG-WGVFDFEPLRTCCTLDYSQGDR--GYITYMLTITVLYLAFPVLVLQSSYSAIHAYFKKTHHYR------------TGLPLKALLFCWGPYVVVCSLACFEDVLSPRLRMVLPVLAKTSPIFHAVLYAYGNEFYRGGVWQFLTGQ-----KSADKKK

>Danio_RPRperopsin_Q66I55

AFTQTEHNIVAAYLITAGVISLSSNIVVLLMFVKFRELRT-ATNAIIINLAFTDIGVAGI-GYPMSAASDLHGSWKFGYMGCQIYAALNIFFGMASIGLLTVVAIDRYLTIC--RPDIG---QKLTT-RSYTLLIVAAWLNAVFWSSMPI--VG-WAGYAPDPTGATCTINWRNNDT--SFVSYTMTVITVNFIIPLSVMFYCYYNVSATVKRFKASNMDWSDQMDVTKMSVIMIVMFLAAWSPYSIVCLWASFGDKIPAPMAIIAPLLAKSSTFYNPCIYVIANKKFRRAIIGMIRCQTRQR-VTINNQL

>Danio_novopsin8_F1QCL1

KLSSAVDYGIGAFLLLIAILSILGNLMVLVMAYKRSNHMK-PPELLSVNLAVTDLGAAVT-MYPLAVASAWNHHWIGGDVSCVYYGLMGFLFGAASMMTLTIMAIVRFIVSL--TLQSPK--EKISK-RNAKILVATTWLYALLWAIFPL--IG-WGKYGPEPFGLSCTLDWRDMKE--HSQSFVITIFLMNLILPAIIIVSCYCGIALRLYVTYKSMNMIKMQRRLMVIAVLISIGFVGCWAPYGIVSLWSIYRPSIPAEVSMLPCLFAKTSTVYNPFIYYIFSKTFKREVNQLSRCGRSNICRPTDAKN

>Danio_novopsin7_F1R0P5

KLSPAVDYSAGTFLLVIAILSILGNAAVLLTAAWRHSVLK-APELLTVNLAVTDIGMALS-MYPLSIASAFNHAWIGGDPSCLYYGLMGMIFSVASIMTLAVMGLVRYLVTG--NPPKSG--SKFRR-KTISILIGVIWMYSLLWAVFPI--LG-WGGYGPEPFGLACSVDWMGYQHSLNRSSFIMALAILCTLMPCVVILFSYSGIAWKLHKAYQSINSGAVERKVTLMGILISTGFIVSWAPYVFVSLWTMFRSSVVPIVSLLPCLFAKCSTVYNPLVYYVFRKSFRREIHQIRICC------------

>Danio_novopsin9_F1R302

KLAPALDIAVGLIIMSIVILSVLGNGVVLVISYRRRKKIA-GSELLCVNLAVVDFLCCIC-FYPLSILSSFSHVWLGRHITCVYYGLGCYIFGLCGMFTIAAISVIRYIKTC--YAIWLEG-------VNIRLVCVATWLIAAVWSSFPL--FG-WGEYVPEPYGISCTIAWRRYHTSTKDAIYVICSFACFVLLPVLLIMVSQCKILLKIYRFSRTLNLRHAEKHLSVMFFCISLGFILAWAPYAVVSFLFIFHKYMAPEGFIFPALFAKSSHVYNPFIYFYFNKAFRQELRNLLFSLPIQI-QLQEQAV

>Danio_teleostNeuropsin_Q5RIV6

KLSKEADIVAAFYILVIGILSATGNGYVMYMTFKRKTKLK-PPEIMTLNLAIFDFGISVS-GKPFFIVSSFSHRWLFGWQGCRYYGWAGFFFGCGSLITMTVVSFDRYLKIC--HLRYG---TWLKR-HHAFLSVVFIWAYAAFWATMPV--VG-WGNYAPEPFGTSCTLDWWLTQASVSGQSFVMCMLFFCLIFPTVIIVFSYVMIIFKVKSSAKEVNNHSLEMKLTKVAMLICAGFLIAWIPYAVVSVMSAFGESVPIPVSVVPTLLAKSSAMYNPIIYQVIDCKKKCVKSCCFQ--AWRK-KKPSKTS

>Danio_novopsin10_A0A0R4IEG0

QLSPSADLSIAVFLIITGVVSVFGNGLVLLVYGQRRKKLR-AHELMTINLAVCDFGYSLL-GAPCIIISSLSHAWIFGETGCIWYGIQGFVFGIGSLITTCLISLDRCFKIC--SFRYG---QWVER-KHASLSVTLVWIYTLFWASLPM--FG-FGSYGPEPFGTSCTINWWRMNSSLTDRIYIFLILSLCFGVPTLIIIASYLAIVIRVYWSSHILTHNSKDLRLTKIAAVVCSSFLIAWTPYAIVSLYSAVMAILKPEFSLIPAIFAKSHCMINPFIYQIMNRDFREDVYDLLCMRRRRS-RSTDGSD

>Danio_novopsin1_A0PJR8

PLSDQGETIIGVYLLILGWLSWFGNSIVIFVLFRQRSTLQ-PTDYLTLNLAVSDASISVF-GYSRGILEIFN---IFKDSTCQVDGFFTLVFGLSSINTLTVISITRFIKGC--HPHKA---HCITN-STVAVCVVFIWIGAFFWSAAPV--LG-WGSYTDRGYGT-CEIDWVKANYSTIHKSYIISIFIFCFLVPVLLMLFCYISIINTVKRGNAMNRQRKIERDVTIVSIVICTAFILAWSPYAVVSMWSAWGFHVPNLTSIFTRLFAKSASFYNPLIYFGLSSKFRKDVSVLLPCG-----REGRDPV

>Danio_MammalianlikeMelanopsin3_E7FEB1

DVPDHAHYTIGSVILAVGITGMVGNLLVMYAFCKSRSLRT-PANMFIINLAVTDFLMCVT-QTPIFFTTSLHKRWIFGEKGCELYAFCGALFGICSMITLMIIAVDRYFVIT--RPLASI--GVMSR-KRALLILSAAWAYSMGWSLPPF--FG-WSAYVPEGLLTSCSWDYMTFSP--SVRAYTMLLFTFVFFIPLFVIIYCYFFIFKAIRETNRAVHRMKNEWKMAKIALIVILLYVISWSPYSCVALTAFAGYMLTPYMNSVPAVIAKASAIHNPIIYAITHPKYRSAIAKYIPCLGVLL-CVPRRDR

>Danio_MelanopsinOpn4_F1QZ18

DVPDHAHYIVAFFVAVIGALGVIGNLLVMYAFFSNKKLRT-PPNYFIMNLAVSDFLMAIT-QSPIFFINCMYKEWVFGEMGCKMYAFCGALFGITSMINLLAISIDRYIVIT--KPLQAI--RWTSG-RRTLIIILVVWIYSLAWSLAPL--IG-WSSYIPEGLMTSCTWDYVTSTP--ANKSYTLMLCCFVFFIPLGIISYCYFFMFLAIRSASRDVQSIKTEWKLAKIAFVVIIVFVLSWSPYACVTLIAWAGYVLSPYSKAVPAVIAKASAIYNPFIYAIIRSKYRDTLAEKVPCLHFLA-QSSRKDV

>Danio_Opsin1ShortWaveSensitive_Q9W6A9

IAPKWAFYLQAAFMGFVFIVGTPMNGIVLFVTMKYKKLRQ-PLNYILVNISLAGFIFDTF-SVSQVFVCAARGYYFLGYTLCAMEAAMGSIAGLVTGWSLAVLAFERYVVIC--KPFGS---FKFGQ-GQAVGAVVFTWIIGTACATPPF--FG-WSRYIPEGLGTACGPDWYTKSEEYNSESYTYFLLITCFMMPMTIIIFSYSQLLGALRAVAAQQSTQKAEREVSRMVVVMVGSFVLCYAPYAVTAMYFANSDNKDYRLVAIPAFFSKSSCVYNPLIYAFMNKQFNACIMETVFGK-----KIDESSE

>Danio_Rhodopsin_Q801U8

LADPWQFKALAFYMFFLICFGLPINVLTLLVTAQHKKLRQ-PLNYILVNLAFAGTIMAFF-GFTVTFYCSINGYMALGPTGCAIEGFFATLGGQVALWSLVVLAIERYIVVC--KPMGS---FKFSS-NHAMAGIAFTWVMASSCAVPPL--FG-WSRYIPEGMQTSCGPDYYTLNPEYNNESYVLYMFSCHFCVPVTTIFFTYGSLVCTVKAAAAQQSTQKAEREVTRMVILMVLGFLVAWVPYASFAAWIFFNRAFSAQAMAIPAFFSKASALFNPIIYVLLNKQFRSCMLNTLFCGKSPL-GDDESSS

>Danio_parapinopsin_A0A0N9NUD7

VMPRMGYTILAVIIGVFSVCGVILNVTVITVTLKYKQLRQ-PLNFALVNLAVADLGCAVF-GGLPTVVTNAMGYFSLGRVGCVLEGFAVAFFGIAALCSVAVIALERCMVVC--RPVGS---ISFQT-RHAVFGVAVSWLWSFIWNTPPL--FG-WGRFELEGVRTSCAPDWYSRDL--ANVSFIVCYFLLCFALPFSVIVYSYTRLLWTLRQVSRLQSAARAEAQVSCMVVVMILAFLLTWLPYASFALCVILIPYIDPVIATVPMYLTKSSTVFNPIIYIFMNRQFRDRALPFLLCGRNPW-AAEAEEE

>Ovulgaris_Dopamine1like_XP_029649638.1

HSNTTKTIIIGLLLSIMILVSVVGNSLVCVAVFKVKQMRK-IGNFYLVSLAIADLLVSLL-VMTFALANDIMGEWVFGRPFCNVWISLDVMCSTASILNLCAISLDRYIHIR--DPLHYE--NWMTK-KRTAIIISVVWVCSFLISFLPI-HLG-WHSPDAASQRTGCDLD--------INPIYAVISSTISFIIPCIVMLSIYCKLYSTAQQHVRNIDGSLSDHKAAITLGIIMGVFLLCWTPFFVVNLTSAACKCIPPLAFKILTWLGYANSSLNPIIYSIFNTDFREAFRRVIL-------TANDDGY

>Pomacea_dopamine1like_XP_025095706.1

EYSLPEKAVIGAILTVMVFVAVVGNLLVCAAVFTDRRLKR-NSNFFIVSLAIADLLVALV-VMTFAIANDIHGRWVFGSIFCKVWISSDIMCSTASILNLCVISLDRYIHIR--DPLRYE--AWMTT-RKTVLFIATVWILSVLISFVPT-HLG-WHEPPMGELQELCVFN--------INPVYAIVSSTVSFYIPCVVMVSIYVRLYVYARRHVQTISYKVSDHKAAVTLGIIMGVFLLCWVPFFIVNPIAAFCSCIPKLVFQILTWCGYVNSCLNPIIYSIFNTEFRDAFRRILFCF-----RRQKDVL

>Mizuhopecten_D1likeDopaReceptor__XP_021361185.1

PYTLVESIVIGTILSLVIILAIAGNILVCVAVFTDRRLKH-LNNLFIVSLAIADLLVAIL-VMTFAVVNDIQGRWLFGSVFCDIWISSDIMCSTASILNLCVISLDRYIHIR--DPLRYE--NWITW-KKVAGMISSVWIMSLIISFVPI-HLR-WHKNPEDYDENACHMD--------LNRAYAVVSSTISFYIPLIVMLAIYIQLYAYARKHAANIGSKMSDHKAAVTLGIIVGVFLFCWLPFFIMNLISAFCEDVPAAVFSILTWLGYANSFLNPIIYSIFNRDFRDAFKRILFCI-----RRHNNGF

>Sof_reti2_MN788449

LFTIWEHSFTGVIYMLIGCCVYVSCGITAIILSRQNTQPK-RKYTTLIYFLLACIAV-NG-ANPFHAASSLAGRWIFGETGCIACGFYGFLAGIFQIWCLFAFCVERYIATN--FKNFY---KTIPS-IYYTMTVITMFGIGLFWAFMPI--LG-WSQYGLEAPGTSCSVDYSVATQ--HYQSLLISMSLVTYGAPLLASYWAVSKAMAALRNTPDEETHLLSEEQLTALAAVFIPIALISWSGFAYIALYAAFTNYITNLAGTLPPLMAKFGCLLYPLFIFVARIRTMPKLSSKKP--------------

>Euprymna_PutativeReti2_SRIE01025459.1

LFTMWEHSFTGVIYMLIGCIVYITCGITAVCLARQNTQPR-RKYTTLIYFLLACIAV-NG-ANPFHAASSFAGRWIFGETGCIACGFYGFLAGISQIWCLFAFSVERYIATN--YKKFC---KTIPS-IYYTLTIIAMIGIGLFWACMPL--LG-WSQYGLEAPGTSCSVDYSVATD--QYRSLLISMCLVTYGAPLVISYWAVSKAMTALKNTPEEENHLLSEEQLTQLAAVFIPIALISWSGFAYIALYAAFTDYITNLAGTLPPLMAKFGCILYPILIFLTRVRTMPKSSIKKP--------------

>Idiosepius_reti2_A0A0H5B8L2

LFTMWEHSFAGAIYMLIGCFVYVTCGATAVMLSRQNTQPR-RKYTTLIYFLLACIAV-NG-ANPFHAASCMAGKWIFGETGCIACGFYGFLAGISQIWCLFAFCVERYIATN--FKSFY---KTIPS-IYYTLTVLTMIGIGLFWAFMPI--LG-WSQYGLESPGTSCSIDYSVATE--HYQSLLISMSLVTYGAPLMGSYWAVSKAMTALKNTPEEETHLLSEEQLTALAATFIPVALISWSGFAYIALYAAFTNYISNLAGTLPPLMAKFGCTLYPIFIFLARVRTMPKVSIKKL--------------

>Ovulgaris_reti_XM_029782381.1

TFSMWEHYVTGALYLIIGVTAFATNGLAAIVLARQNTQPR-RKYSMLIYFLLTSALV-NI-GFPLNSSSSIAGRWLFGNFGCHFYGFIGFVVGISHIWMLFAFCIERYIATC--HREFY---KRMPS-IYYTLLIAAMYSVGTFWAVMPL--LG-WGEYGLEPTGTSCTISYINATD--SFQSLIMSMVIVTYSFPIVVSCYAVSKAWTALGNMSDSERDLLSEEQLTAMATIFIPIALVSWSGFAYVSIYSAITDTLSHLSVTMAPLLSKSGVILYPLIILVAGIRSIPKAESKKP--------------

>Obimaculoides_reti_XM_014915229.1

TFSMWEHYVTGALYLIIGVTAFSTNGLAAIVLARQNTQPR-RKYSTLIYFLLTSALV-NI-GFPLNSSSSIAGRWLFGNFGCHFYGFIGFVVGISHIWMLFAFCIERYVATC--HREFY---KRMPS-IYYTLLFAAMYSVGTFWAVMPL--LG-WGEYGLEPTGTSCTISYINATD--SFQSLIMSMVIVTYSFPIIVSCYAVSKAWTALGNMSDSERDLLSEEQLTAMATVFIPIALISWSGFAYVSIYSAITDTLSHLSVSMAPLLSKAGVILYPMIILVAGIRSIPKAESKKP--------------

>Doryteuthis_reti1_comp110548_c0_seq1

KFTTLEHCVTGIVYGLLGCFVFATNGICLVLLTKQSPKPR-RKYAILIHVLITGMGV-NG-GFAAHASSSIAGRWLYGSVGCQIMGFWGFFAGMSHIWMLFAFGMERFVAVC--YRDFY---NTMPS-IYYTVVIGLMYVFGTFWATMPL--LG-WATYGPEVHGTSCTINYSLSDE--SYQSFIFFLAAFSFVCPLVSAWYAITKAWSGLSALPDAEKDILSEEQLTALAGIFILISSISWSGFGYVAVYSAITHSLTHLGGHMAPLMSKIGCALFPVLIFMATSRTIPKGDTKKP--------------

>Sof_reti1_MN788448

QFTMWEHYFTGSIYLVLGCFVFATCGICLILLTRQSPKPR-RKYALLIHILITCMAV-NG-GFAAHASSSIAGRWLYGSIGCQLMGFWGFFAGMSHIWLLFAFGMERYVAVC--HREFY---NQMPS-IYYTVIIALMYIFGTFWATMPL--LG-WGTYGLEVHGTACTINYSLSDE--SYQSFIFFLAVFSFVFPMVSGWYAVTKAWSSLSSIPDAEKDILSEEQLTALAGIFILISMISWSGFGYIAIYSAITHSLSHLAGHMAPLMSKSGCALFPILIFIATARTIPKIDTKKP--------------

>Euprymna_PutativeReti1_SRIE01025459.1

QYTMWEHYFTGSIYLVIGCFVFAACGICIVYISRQSTKPK-KKYAILIHVLITCMAV-NG-GFAAHASSSIAGRWLYGGFGCQMMGFLGFFGGMSHIWLLFAFGVERYIAVC--HRDF----------------------------------------YGLEVHGTSCTINYMVSDE--SYQSFVISLALFSFVFPMFSGWYAVTKAWSGLSSIPDAEKDILSEEQLTALAGVFILLSMVSWSGFGYIAMYSAVTHNLSHLAGHMAPLMSKCGCALFPVLIFLTSVRTLPKSDTKKP--------------

>Idiosepius_reti1_A0A0H5ANL2

QFTMWEHYFTGSIYLILGCFVFSTCGIALVLLARQNVKPR-KKYAILMHVLITAMGV-NG-GFAAHASSSIAGRWLYGGFGCQLMGFWGFFAGMSHIWMLFAFGIERYIAVC--HREFY---NQMAS-VYFSVIIGLMYIFGTFWATMPL--LG-WGTYGLEVHGTSCTINYMLSDE--SYQSFVIFLALFSFVFPMVAGWYAVTKAWSGLSLIPDAEKDLLSEEQLTALAGIFILLSMVSWSGFGYIAIYSAVTHSITHLGGHMAPLMSKFGCALMPVLLFVTTARTLPKSDTKKP--------------

>Nautilus_reti_A0A0H5ANK7

AFTQTEHTVTGILYIVLGILGIIGNGTLAVMLARLKTTQA-SKYQLHIQFAIANILV-VA-GFPFTGSSSIAGRWLFGSVGCQAYSFEGMLAGIASIGFVIAVCIERYVATC--CKETY---RTKYP-NSSNTIVLLIWGNALFWGLMPL--LG-WCRYGLEPTGVSCTINYQIVDQ--QYKSFILALFCCCFAILWPIALICLMKAYSALARTPASEKEAFTEEQITAMSFTSLMLSVIGWTPFAFICLYSLLYDQISHLGATLPPLLAKGSTVLHPLLYLVAGSRLQGSFSSRRKSQ------------

>Mizuhopecten_VisualPigment-likePeropsin_XP_021349313.1

MFSAWEFQCVGIAYFIFGAAGVLANAFTVVTFMRETPISS-PRHILQLNMAVANLLV-CA-PFPFSGLSSFRGKWLFGDLGCQLYGTESFLGGMAATTFIPVVCVEHYLASC--KKDFY---DTLSS-GTWWTVAMLCWMYAALWAILPL--FG-WNSYAIESSGVACGINWLKKDS--NHMTYLQAMVITASILYVMAFYGLYQSRVHW--------NNWFTERQQAWICLAFMCIMFIGFGPYAILGLWAALTDTVSTLAIIIPSLACKASTSLYPIPYLVASDKFRAAYLGYRV-------TEHEAKT

>Argopecten_retinochrome_APB88020.1

MFSVWEFRFVGIAYFVFGSAGVLANAFTILTFMRESPIGS-PRHILQLNMAIANLLV-CA-PFPFSGLSSFKGQWLFGELGCQLYGTESFLGGMAATTFIPVVCIEHYLASC--KKDFY---DTISS-STWWTVAMLCWMYAALWAILPL--FG-WNSYDVESSGIACGINWLKKDA--SHITYMQAMVITYLILFVMAFYGLYQSRVYW--------KNWFTERQQAWICLAFMGIMCIGFGPYAILGAWSALTDTVSTLAIIIPSLACKASSSLYPLPYIVASDKFRAAYLGYRV-------SEQEAKA

>Crassostra_VisualPigment-likePeropsin_XP_011438181.1

EFSEVEHKGVAILYFIFGVSGVLANSFVLKTFMKEGVLVS-PKNILHINLAFSNILV-VL-GFPFSGLSSWHGKWVFGIRGCQLYGVESYTGGMACPAFIFTLCLERYLANR--QRHIY---DTMTT-GTWWLIALGVWLHAITWAILPI--LG-WNSYSMEASGVSCGLSWLKHDF--NHASFIMVMTIEYATLFLLAILF--------LRSAKSYVKNWFTEKQLVWITFAFLMIAGVGWGPYGYFGIWSQRTKSVSMLAVTLPPLFAKASASLYIVPYLAASDHFREAIVGGAT-------PVKKDQ-

>Mizuhopecten_VisualPigment-likePeropsin_OWF48091.1

HIPSIGHDIIGGVLIVTMAVGICTNSLGIAIFIKDKTLRS-PTNLFIAGLALCDLSMLVV-ATPLPTASSFAHRWLWGHTGCVFEGFMVYFLGLTSLYLLCAISVDRYIVIA--LPLKI---ALVTK-RAATLTIVACYGLGFFWAMLPL--VG-WNSYQLEGLMTSCSVVWNTSNP--KDYSYNVVIFFTCLIFPIGVMVYCYYHVYMT--------RNLEVERKISRTILVMIGVFIGSWTPYSIVSFWAAFGVDIPVAVAGVPPYIAKTASVWNPIIYICTNKQFRRSFFNILPFANLQR-ESEEEQV

>Mizuhopecten_VisualPigment-likePeropsin_XP_021355900.1

AFRRGMLQATAIFMLLVAIFGSLFNSMFIYILLEQSIRKS-RTNIYVITLCVASILIAVF-IVPSLGISSLADNWVFGEHGCILHGFAMTALGLFQIFILTVMSFEKYIIMA--KKSWE---RLLSR-TGTTVTIIGCSIIGLFLGSCPL--IG-WNSYKLEEQKTACAIDWSDRSP--SALCYTVLLMFIGLVIPVSIMIFSYVNIFLVIRGHRHKLNMLKREIKVIKTMFILVCAFVFSWLPYSAISLYAVFDDKVNPVLGMLPALFAKASVIWNPIIYMFINQSYKKVLKEKLSCK-----CIRRGDN

>Aplysia_retinochromelike_XP_005094221.2

GFTKFEHASVGALYMLFCIVGVTLNLLTALTFYKDTKLIKGSQPWLHILLALANVGV-VA-PSPFPASSSFSGRWLYGSTMCQIYAFEGMFIGIAAIGAVIALCIERYIACQ--RSGA----NDTQG-WFYGWSITLVLGNALFWAIMPL--LG-WSRYSVEHTGTSCSIDWKNPDE--SFVSYIMTLEVFSFGIPMMSAFFCLISASPRPQPQGGATKGCFSEDQLRLLCYVFIGFVLVGWGPFAYLCTLAVFSDGISMLAAAIPPLACKAMVSAYPLAYAVVSPRFRQSFLALIGGG-----EKKKE--

>Lottia_hypotheticalProt_XP_009060307.1

EFSSFEHSIVGITYMVIGISGTLLSLLVALTFIREKGLFKYGRAWLHISLAIANVGV-VG-AFPFSGSSSFSGRWLYGSGMCTFYGFIGMFFGIAAIGNVFALCVERYLVSK--KKDSV---DKVSN-QFYWMITALVWINAFFWGIMPA--LG-WTSYDIEPSGTSCTIKWQNYDS--GYPSFMAMLSLTCFLIPLPVALICLILSGTD--------KTYFREDQLRSTCTFLLILALIGWGPYCFICIWALFADQVSMLAAVIPPLAAKTMVLLYPVAYCQGNKRFKNAFLGMFI-------NESPKQQ

>Ambigolimax_retinochrome_BBH84663.1

GFTKFEHATVGVLYLLFCVVGVTTNFLTALTFYKDARLLSNGKPWLHICLALANIGV-VA-PSPFPASSSFSGRWLYGKTVCQVYAFEGMFVGMVAIGSVIGLCLERYYIANTLRSKS----KETSG-WFYGWTILLIVGNGFFWAVMPL--LG-WSSYDIEHTGTSCAINWKNPDE--SFISYMLTLMVFSFALPSAVAFFCLYSTHAKPVDQSTPALGPISEDQMQSLCYVFLLLMLTGWGPFALLCLVTILEGGLSMLAGCIPPLTCKLMVSMYPLAYAIISPRFKQSFKALLGGT-----DKKQD--

>Pomacea_VisualPigment-likePeropsin_XP_025113272.1

EFTRFEHATVGALYMIFGVGGVVTNLLIILAFFKDKALLRGSTSWLHVSLAFANICV-VA-PAPFPASSSFSGRWLYGDKVCQAYAFEGMFVGIAAIGAVFSLCVERYLVSK--REDAM---QQNPA-AFYWVATVINISNALFWSVMPL--LG-WSRYSVDHTGAACAIDWKSGDE--SYASYMTMLCFFPFALPFAFAIACLYASIPKAPALTSASTSSFTEGQLRWLCLCFLVLVLVGWGPFAWLCLWAMVGDGVSMLAATIPPLACKFTTMMYPLTYNVINPRFRAAYFSILGTS-----ATEETVK

>Biomphalaria_retinochromelike_XP_013096913.1

GFNRFESATVGSLYMLFCVVGVTTNLLTALTFYKDTKLLKTSKPWIHVCLALSNVLV-VA-PSPFPASSSFSGRWLYGSSLCQFYAFEGMFVGIAAIGAVIALCIERYMVAS--KPNM----KENPG-WFYTWSILLVVGNAFFWAVMPL--LG-WSRYTIEHSGTSCAIDWKNPDE--SYISYMVSLEIFSFLLPVSLAVYCLRPTAGQPPEPSTSGMAPFNENQLKSICYIFLGLVIVGWGPFAFLCTSTMFGGGLSMLAASLPPLACKCMVSAYPLVYAVASPRFRHSFTSVIGIF-----DQKQD--

>Lingula_VisualPigment-likePeropsin_A0A1S3JA52

ELTGLERGILGCLYVIAGIFGTTGNLLTLVTWGRGLQELG-CRAWLHMNFALSNILIVVV--FFFPASSAFAGRWLYGEVACQSYGFEGMIAGMGQIGALTALAWGQANVDA--DA------------DSRKRSILFGWLFASVWAIGPL--IG-WSSYGIEAGGVSCTFNWQVVNV--SYVTYAVMIWAFGFVIPYFLSINWVKSTACI--------KDWSNYPEINTVIGWCIFVSIIAWIPYGLAAAWPLFASTVPKIVGVYAPVSAKLGTCFYPWIHIWHSARFRQTVSSLLFGTTSSA-STAEKEN

>Platynereis_Peropsin_W5XRX9

QHDFLFHSITGIGYLIIGILGIIGHVTLAALFSRENLVSR-GSAAVHVAMAISNAGV-LA-GFPFTASSAFAGRWLFGEAGCQFYAFEGMFFGISSILLLGVLSVDRFINIH--WPQYY---GDLYL-RPYWLAILVCYLTAAFWSTVPI--VG-WARYALDKTHVACVVDWANPTS--SYKSYIFAITMSCFMLPYALMAIGFIRTCLGRKATSTNV----SDRDHDLIVRSLSIVSMVTWTPFAILCLQFLVRDDTSITMAAMPALICKAVTAGVPLVYAVCSAEIRYSIKHMFS-------TCPERKR

>Xenopsus_VisualPigmentlikePeropsin_XP_002934288.1

VFSQSEHNIVAAYLITAGVISILSNIIVLGIFVKYKELRT-ATNAIIINLAFTDIGVSGI-GYPMSAASDLHGSWKFGYVGCQIYAGLNIFFGMASIGLLTVVAIDRYLTIC--RPDIG---RRISG-RHYTAMILAAWINAVFWSVMPV--VG-WSSYAPDPTGATCTINWRKNDV--SFVSYTMSVVAVNFVVPLMVMFYCYYNVSRTMKGYGSRSADWSDQTDVTKMSMVMIVMFLVAWSPYSIVCLWSSFGDKIPPAMAIIAPLFAKSSTFYNPCIYVIANKKFRRAILSMVQCKSRQE-VTLDNHF

>Xenopus_Opsin5_F6UZB2

KLSREADIFAGVYLMAIGILSTLGNGYVIYMACSRKKKLR-PAEIMTINLAVCDLGISVT-GKPFAIVSCFSHRWVFGWNACRWYGWAGFFFGCGSLITLTVVSLDRYLKIC--HLRYG---TWLKR-RHAFIALAVIWAYATLWATLPL--VG-VGNYAPEPFGTTCTLDWWLAQASVKGQIFVLSMLFFCLLFPTMVIVFSYAKIIAKVKSSAKEVNNHTLEIKLTKVAMLICAGFLIAWFPYAVVSVWSAFGQSIPIELSVVPTMMAKSASMYNPIIYQVIDCKPAC-------CK------------

>Chiton_opsin_APF30602.1

PMSIFEHSIVGVLYFTIGIIGTVGNLLTFIVFMYQNPVKS-PRNLVFANFALANTFV-VA-PFIIPGSSSFVGRWLFGDTACQAYAFEGMLAGIGAVGALIVLGVERYLAIC--CKDLY---LNLSS-GNVMCMILLGWLNALFWAAMPL--LG-WCRYSIEPSGTSCTLDWQVIDD--QYVSYVTGLTTFCYCIPFVVLFLCLIRASNSLSSETESAEEWFSEKQLLKLTWVFTLTALIGWGPYAYVVIWPFFNDSLTAVLAVLPPLMAKSSTMIYPFAYMSQSSTFRKAMFLTMGCG-----EQEKKAK

>Ovulgaris_melanopsinlike_XM_029798286.1

TVSAEGGVAVATVLILFSLVAVTENGAVLFTFYICRDLIT-PTNLFILSLTVCDFCIALF-GNPFVVVSGLARGWYFGHFGCIFYGFFMTFLGLTSISLLTAISVDRYILIV--HTMRA---VTIDF-RAAIFSVIGCLLYALFWSGVPL--FG-WNLYVQEASGLACSVQWQGSTT--SDIAYIVLMLIFCLAIPLLLIGYSYIQIFIA-----------------------------------------------------------------------------------------------------

>Sof_xeno2_comp250920_c0_seq2

GVSAEVGILVAVILTLISFLAFVQNGILLTTFYFCRDLLT-PTNLFILALSVCDLLVAGL-GNPFVVAASMYKHWYFGHSMCIIYGFIMTFLGLTSITLLTAISLDRYILIV--RTMRS---VTIDC-RIALRAIGGCVLYALVWSGMPL--LG-WNEYVLEASGLACSVNWQSKSP--ASMSYIILLLIGCLFLPLGFIGFSYTRIFVRVYKKSHSNINSRIEMKVAKTIFFVTLAFLASWLPYAIVSLLFVLGDIITPAAEISCAILAKCSVIWNPLIYVMTNAQLRHSMLDVFRCNRKEY-AMNGREE

>Euprymna_PutativeXenops_SRIE01029988.1

VITTEVGLSVALVLAFFSAGAFFLNGSVIILFIVRKELLT-PTNVFIVTLSVCDFLIATL-GNPLAIVSSAMRHWYFGRTVCVWYGFTMTFLGLTAISLLTAISVDRYVLIV--HTMRT---VTISM-RTSVVCVAGCSFWALFWAVMPL--LG-WNGYVTEINGIACSVDWQGKTT--AATSYLIMLLIWCLLIPMMALGFSYIKIFMTVYKKSHSNINSRIETKVAKTIFFVT----------------------------------------------------------------------------

>Doryteuthis_xenopsine1_comp31393_c0_seq1

SISSEVGLTIALILAMFSVGAFFLNGAVITLFLIRKDLIT-PTNVFIMTLSLCDFLIAIL-GNPLAIVSSAMRRWYFGRSVCVWYGFTMTFLGLTAISLLTAISVDRYVLIV--HTMRT---VTISM-RTSIVSVICCGIWGLFWAMMPL--LG-WNGYVTEINGLACSVDWQGKTE--AAVSYIILLLIFCLVIPMMLLGFSYIRIFMT-----------------------------------------------------------------------------------------------------

>Doryteuthis_xenops2_comp37005_c0_seq1

GVSTEVGILVAVILSFFSLVAFIQNSILLTTFFFCRELLT-PTNLFILALSVCDLLVAGL-GNPFVVASSLYHQWYFGHSLCVVYGFFMTFLGLTSITLLTAISLDRYILIV--RTMRS---VTIDC-RIA------------------------------------------------------------------------------------------------------------------------------------------------------------------------------------

>Sof_Xeno1_MN788450

SVSSEVGLTVALILSMFSAAAFLLNGAIVIFFIVRKDLIT-PTNVFIMTLSVCDFLIATL-GNPLAIVSSAMRRWYFGRNICVWYGFTMTFLGLTTISLLTAISLDRYVLIV--HTMRT---VTISM-RTSVVCVICCAIWGLFWAVMPL--LG-WNGYVTEINGLACSVDWQGKTP--AATSYIIMLLICCLLIPMLILGYSYIRIFMTVYKKSHSNINSRIEMKVAKTIFFVTLAFLASWLPYAIVSLLFVLGDIITPAAEISCAILAKCSVIWNPLIYVMTNAQLRHSMLDVFRCNRKEY-AMNGREE

>Idiosepius_xenopsin_A0A0H5ATB0

TVSREAGLIVALVLAMFSAASFLLNGAVIVLFMLRKDLLT-PTNVFIVTLSVCDFLIATL-GNPLAIVSSAMRRWYFGRSVCVWYGFLMTFLGLSAISLLTAISVDRYVLIV--HTMRT---MTISL-RTSVVCLICCGLYALFWAVMPL--LG-WNGYVTEINGIACSVDWQGETP--AATSYIILLLICCLIIPMFLIGFSYIRIFMTVYKKSHSNINSRIETKVAKTIFFVTLAFLASWLPYAIVSLLFVIGDIITPAAEISCAILAKCSVIWNPLIYVMTNAQLRKSMLDVFRCNRKEY-AMNGREE

>Leptochiton_xenopsin_A0A288XNQ3

YLPPYAHYCIGSFLVLIGFLGFSENAAVLYVFAKSKQLRT-ATNMFIISLAISDFSMACL-GNPLASTSSLSQRWLYGETGCKWEAFVVYFFGLASIYNLTCISFDRYVVIV--KPLLG---PKITK-RIAGLAILIGWFLAFFWAVVPL--LG-WSRYGLEAAHTSCSVVWQSKEP--VDISYTMTIFVFCFIVPVGAMVFSYYHVFMTIRNVSRTGRNLRIEKKMAKTIVYMASAFLISWTPYAVVSLWAAIGNDISPLAGTLPAILAKSSIIWNPIIYVATNKQFRHAFYEVVPCAGLKE-AMLEREQ

>Biomphalaria_parapinopsinlike_XP_013088307.1

DVSSLEYALIGSALAAVAAVGTIFNGMAITVFFRYRELRS-PTNSFVIALCVCDFFMSVI-GAPIPTYYAFMNTSITSSTLCSLDAFTVYFLSCTSIYLLAAISVDRYFIIV--KPVSS---LVITQ-KIATLAIFICFSVGLFWALMPL--VG-WNGYSLEGIGVACSVTWNRPDV--LFNSFIIALFLACFLIPLLVMAFCYLSIIFTVRKIFRGAKHYTIECQMIKTVILMIVLFAVSWLPYAIVSFSMAFGSNMTRLVETVPALIAKSSCIWNPIVYVSMNSHFRAGFLSLLPCMKKSF-PTGDSSK

>Argopectans_xenopsin_A0A1J0CN88

QISNTQYAILAIFMFTVFLGGVIFNGLFVYVFLVHSKLKT-RPNILLISLCISSFLIAAL-AIPFVGASAISRKWLFGQFGCVFHGFIVTALGLTQIAILTVLSFEKYIAIV--KCHWS---HRLTQ-SATLLLLFGCFMYGFLLAAYPL--LG-WNRYTMEDGNISCSIDWTSRSA--IDLSYSISLLVIGLVVPLAVMSYVYISILLLIKKQTSISRASKRDVKVMKTILLLVFAFIISWLPYSIFAMTSITGYDIHPLLGTLPSLFAKSSILWNPLIYVCRNRSFKRALLETFPTLAVFY-RCTNKCR

>Argopectens_xenopsin_A0A1J0CN90

YMQKSVLHITAISMLFLAMFGFFFNSLFIYILTTHTNLKK-KNNLYVITLCISSILIAVL-VVPTLGISSLEDRWVFGNIGCSLHGFTMTAFGLFQIFIVTAMSMEKYVIVV--RNNWN---SFVSI-TGTRCSIAACLLLGVAIGSCPI--LG-WNSYKLEEQKISCSIDWSDKSK--MALSYTYSILILGFVAPFSMMVFSYVSIFMEIRGHIFNLNMLKREVKAIKTMLILVCAFLTSWLPYVIISMYAMFNEFLNPWLNMLPSLFAKASVIWNPIIYLFINKSFRNALMDKIPCH-----SLLRDSQ

>Argopectans_xenopsin_A0A1J0CN89

SMSSMYYNLFAVLLFLTWMFGSFFNGSALLIFTKNKHLRT-PTNMFVIGLAINDFCMSSV--ALFAASASYNQGWYHGDTVCAMEGFLVYVLGLTDLYLLCAISFDRYIVIA--KPLSA---SKINH-GVALLAIVGCWLGGTFWSVVPF--FG-WNYYHLELSNVSCGVSFEGNDP--SIQSYLLSIFIFCFLLPLGLIIFSYNGVYQTVRNVARSGKNLRVEKKMAKTIAVMILVFLIAWLPYTIVSFYQAFLAYIPLIVTGLPPVFAKCSAALNPIAYIGTNKQFRMAFYELMP--GMKK-TMIKREE

Argopectans_xenopsin_A0A1J0CN98

LLPTYAYATIGVLMTGASTFGVALNGIFIYVFWKQTALKT-PTNYFILSLSILDFLMSLF-GLPMIAISSFARDWIFGDKGCVYYGFIMTLLGISTISILAAISFDRYIVIV--KTHLK---PMISQ-RVATCIIVGCLLYGLAWAIAPL--LG-WSKYVLEGINISCSVNWNSDNF--GDASFCVALLILVLVIPIIVILFCYGNIFYKVKTSGNNQRGSKMDRDVAKTILWMTLAFIFSWFPYAIFSMTAVIGGVIPSGLTVLPTLLVKLSVVWNPLIYTYRNREIRRSMIELLPCMRIQG--------

>Crassostrea_melanopsinBlike_XP_011421213.1

ELPGPMYVVLAVYLFFLTFFGILVNGAVIYLYFSRREIAT-VSNMYIVALCLCGFLIATL-GIPFAAASSIRHHWLFGDGMCKLHGFLLTGLGIVMIALMTGIAIDKYIHIV--WFQAH---RKVTK-SFALGIITLCYVYGVIWGILPL--FG-WNKYILEPARLTCSVEWTG-DF--SNHSYAITILFTGLLIPVGVIASLYSSILKKIHLQRKSSKMIKREKKVAITLFLMVGSFIVAWLPYSIYGFICILGYDIPLVWHTIPSVFAKASILWNPLIYASRSKVMKKALAETFPFLRWLI-RNPDKVQ

>Crassostrea_melanopsin_XP_011412506.1

FIPQPLYYIIGTGLLFVFTFGSFMNFTGLLVFAKNKHMRS-PTNTFIISLLMGDFGMSIC--SFISMTAHYNRFYLWGDNVCTFEGFWMYFMGLTNMYTIMGISFDRYIVIA--KPLQA---SKITT-RVAVAACLAIWFQGFAWAAFPF--LG-WGRYTYEAGRTSCSVQWDTDDI--ESASYNISIFIWSLFLPLMLIFYCYYNVFMTIRHVARNGKNLRIEKKMFKTIVYMLVSYVGSWTPYSIVSLWAIFGEDIPPYLMTVPAVIAKSACIWDPLIYVGTNRQFRMAFYNTLPCDGLGK-MLIKREE

>Crassostrea_parapinopsin_XP_011412507.1

PIPIPLYYVIGSGLLIVVTLGPFMNITSLAVFAQNKHLRS-PTNIFVISLLLGDVGMSCV--ALISMVAHFNRYYFWGDRVCVFEGFWLYLMGLTNLYTHAVIAVDRYIVIA--KPLSA---HRVTK-RVAVVAVLVVWIQGLLWASFPH--FG-WGKYTYEPARTSCAVEWDSKEI--GSASYNVAITIWSLCIPLGLIVFSYYRVFMTIRHVARSGKNLKMEKKMFKTIAYMLASYLWSWTPYTVVSVWAIIGEDIPVYIITIPAVVAKSSCIWDPLIYLWTNRQFRIAFYKTMPCKSLGE-KLLQRDE

>Lottia_GPCR_V3ZWI5

------------VMTMYCITAVLFDGCAMLVFLKNKNLRS-PTNLFILGLNVCDFLMATI-AAPLSGISSFHHRWLFGTIGCTYEGFAVYFLGQTSMYLLAAISFDRYYVIA--KPLQA---SKITH-RVATIAVLACYAGGFFWAFVPL--VG-WNEYTLEGAKISCSVVWESSNP--VYTSYIFTIFITCLVIPLAVMFFSYFNVYMTVRKLSR--RNFRIEKKMFKTIIAMCTTFVAAWMPYTVVSFYGAFYGSVAPALGTMPALFAKCAGLLNPIIYVATNKQFRTAFYQLVPC-------------

>Lingula_visual_pigment-like_receptor_peropsin_XP_013400870.1

-------------------------------------------------MCVCNLVEAAL-SFPLMASSSFAQRWLFSKIGCQIYAFIIFSLGLTGIFHLVIVAVDRYLVIC--KD------TKVKK-GSAIKGSIACWLSGILWATLPF--LG-WNGYIFERAHLSCCINWYSKDP--QDLSYIVTIMVFCLFVPMAVMSFTYGRIYITIKNSHQPSRKRKRQTKMLKMIIAMTVAFLLSWSPYAIVSLWAAFDDHIPPTSDVIPALLAKASVIWNPIIYVAMNQQFREGFLQFIG-------DIGRRQH

>Lingula_rhodopsinGQ-coupled_XP_013382905.1

YLSPAIHYACGIAMTLGTILGIFGNTSILLMFLKFKNLRS-TTNLFIMSLNVSDLMLAVF-GCPMSGISSFALKWVFGDVGCVLHGFMMYFAGLSEMYCLTAIAIDRYIVIA--KPLLA---PAITQ-TKALIAIVACYVGGLMFAMAPL--FG-WSKYTYEGAGTTCGIDWITKRP--QDVSFAITILIACYLTPVGIMLFCYLNIYLTVLSSTRSGRNARLERKMALMTVAMLGNFLLCWTPYALVSCIATFGDSMPLLLVHIPSMFAKCAAVMNPIIYVGLNQQFRDAFLEMCPCCSGIV-ACFASKS

>Lingula_melanopsin-like_P_013397676.1

HLTTTEHIVIAVIMLNLSFLGMVMNVGVILTYILNKELQN-SVNMFIASLSVGDALMSFL-A-VLVAVNNFAEEWLYGDAGCTFYAFSMTFLGLAAINQMSAVAADRYVVVT--RPHS----VLVEK-PAAVTTIVICWLAAGIWAVMPL--VG-WSSYQPEADGTHCSIHWETEDE--NARSYIISLFVLEFFLPIVIICFCYGNIFHKFKFIGTKVRRKRVERRLAKVICLMIGCFLASWTPYAVFSMWAAFGDSIPSLARVVPALLAKLSCVWDPIIYVGGNAHFRTHFVKYLPRMLTRK-CIEEGED

>Mizuhopecten_VisualPigmentlikePeropsin_A0A210QHL3

HIPSIGHDIIGGVLIVTMAVGICTNSLGIAIFIKDKTLRS-PTNLFIAGLALCDLSMLVV-ATPLPTASSFAHRWLWGHTGCVFEGFMVYFLGLTSLYLLCAISVDRYIVIA--LPLKI---ALVTK-RAATLTIVACYGLGFFWAMLPL--VG-WNSYQLEGLMTSCSVVWNTSNP--KDYSYNVVIFFTCLIFPIGVMVYCYYHVYMT--------RNLEVERKISRTILVMIGVFIGSWTPYSIVSFWAAFGVDIPVAVAGVPPYIAKTASVWNPIIYICTNKQFRRSFFNILPFANLQR-ESEEEQV

>Platynereis_copsin_Q5SBP8

AFTATDYNICAAYLFFIACLGVSLNVLVLVLFIKDRKLRS-PNNFLYVSLALGDLLVAVF-GTAFKFIITARKTLLREEDFCKWYGFITYLGGLAALMTLSVIAFVRCLAVL--RLGSF---TGLTT-RMGVAAMAFIWIYSLAFTLAPL--LG-WNHYIPEGLATWCSIDWLSDET--SDKSYVFAIFIFCFLVPVLIIVVSYGLIYDKVRKVAKTGSVAKAEREVLRMTLLMVSLFMLAWSPYAVICMLASFGPLLHPVATVIPAMFAKSSTMYNPLIYVFMNKQFRRSLKVLLGMGVEDL-NSESERA

>Xenopus_parapinopsin_Q75R39

LMPRIGYTILALIMAVFCAAALFLNVTVIVVTFKYRQLRH-PINYSLVNLAIADLGVTVL-GGALTVETNAVGYFNLGRVGCVIEGFAVAFFGIAALCTIAVIALDRVFVVC--KPMGT---LTFTP-KQALAGIAASWIWSLIWNTPPL--FG-WGSYELEGVMTSCAPNWYSADP--VNMSYIVCYFSFCFAIPFLIIVGSYGYLMWTLRQVAKLGTTSKAEVQVSRMVIVMILAFLVCWLPYAAFAMTVVANPHIDPIIATVPMYLTKTSTVYNPIIYIFMNKQFQECVIPFLFCGRNPW-AAEKSSS

>Xenopus_MultipleTissueOpsin_F7EFF0

NLSPTGHLLVAVFLGVIGSLGFFNNLVVLILFCQYKVLRS-PINMLLMNISLSDLMVCIL-GTPFSFAASTQGHWLIGEIGCIWYGFVNTLFGTVSLVSLAVLSYERYCTML--RSTEA---DLTNY-KKAWLGILVSWIYSLVWTLPPL--FG-WSKYGPEGPGTTCSVNWHSRDA--NNISYIVCLFIFCLALPFAVIVYCYGRLLFAIKQVSGVSSSRAREQRVLIMVIVMVVCFLLCWLPYGVMALVATFGKIISPSASIIPSVLAKSSTVYNPIIYIFLNKQ------------------------

>Xenopsus_parietopsin_Q1L0I6

IFPRSGYSILSFLMFLNAVFSICNNAIVILVTLKHPQLRN-PINIFILNLSFSDLMMALC-GTTIVVSTNYHGYFYLGKQFCIFQGFAVNYFGIVSLWSLTLLAYERYNVVC--EPIGA---LKLST-KRGYQGLVFIWLFCLFWAIAPL--FG-WSSYGPEGVQTSCSIGWEERSW--SNYSYIISYFLTCFIIPVGIIGFSYGSILRSLHQLNRKITNPREEKRVVIMVLFMVLAFLICWLPYTVFALIVVINPYISPLAATLPTYFAKTSPVYNPIIYIFLNKQFRTYAVQCLTCGHINL-DSLEEDT

>Apis_pteropsin_Q2YD70

QVSPVMYIGAAIALGFIGFFGFTANLLVAIVIVKDAQILWTPVNVILFNLVFGDFLVSIF-GNPVAMVSAATGGWYWGYKMCLWYAWFMSTLGFASIGNLTVMAVERWLLVA--RPMQA-----LSI-RHAVILASFVWIYALSLSLPPL--FG-WGSYGPEAGNVSCSVSWEVHDPVTNSDTYIGFLFVLGLIVPVFTIVSSYAAIVLTLKKVRKRAASGRREAKITKMVALMITAFLLAWSPYAALAIAAQYFN-PSATVAVLPALLAKSSICYNPIIYAGLNNQFSRFLKKIFDARGSRT-AVPDSQH

>Platynereis_neuropsin_AIT11645.1

-------------VTQLGVLSILLNVLVILTVLCRRRSVS-PLEIYVINMAVVDLCPTVL-AYPSTTASAFNHGWILGDT------------------------------------------DMVNR-RFVLLTLIPVYGNALIWCFTPL--VG-WGRYGPESSGISCALEWHH-----LPLSYVIKIFVTGFLMPVAIMIFCYGCIIREVYITQKGTIRKRMDIYMIKMTIMMTLCFLVAWTPYAVVAFLATEPWEISVTLSVASSFLAKSSSFYNPIVYVFTVKRFRREVIEVLRCS-----VTKDTNA

>Xenopus_Mammalian-likeMelanopsin_Q0QFY9

-----VHYVVGAVILAVGITGMLGNFLVIYAFCRSRSLRS-PANMFIINLAITDFLMSVT-QAPVFFATSLHKRWIFGEKGCELYAFCGALFGITSMITLMVIAVDRYFVIT--RPLTSI--GVMSK-KRAVLILSGVWLYSLAWSLPPF--FG-WSAYVPEGLLTSCTWDYMTFTP--SVRAYTMLLFCFVFFIPLFIIIYCYIFIFKAIKNTNRAVQKMKNEWKMAKIALIVILLYVVSWSPYSTVALLAFAGYILTPYMNSVPAVIAKASAIHNPIIYAITHPKYRMAIAKYIPCLGSLL-RVKRRDS

>Xenopus_GPCR_F6T3T2

-----------------AILTVLGNCAVLATAVKCSSHLK-APDLLSINLAVADLGMAIS-MYPLAIASAWNHAWLGGDASCLYYALMGFFFGVSSMMTLTVMAIIRYRVTS--SFKYSG--CTIEK-KAVCILIMCIWLYALLWAVLPL--LG-WGRYGPEPFGTSCTIAWGDFHHSSNGFSFIISMFILCTISPAVTIVVCYSGIAWKLHKAYQEINSTKVEKKLTLLAILVSFGFLISWTPYAAVSFWSLFHSYIPPVVSLLPCLFAKSSTAFNPMIYYAFSKTFRRKVKHLKCCCRVHFLQSENSVE

>Argopecten_Neuropsin-like_A0A1J0CN87

KLTPMEDRLVALYLGVVGIISIFLNLLVLFVCFKKRQTLK-TIDYFIVNLAITDLCLPLF-GFPLVVTSSVKHEWQFGVYGCYVYGFMGFFCGTVSISTLAMMSFVRYMSVC--EMQKS---VHLN--NNTPGLVLLTYLYACVWSLPPF--MG-WGDYGVEPHGTSCTLNWS------GSRSFVTAMLIMCIVLPVVIMIVCYGRVLLFLKKSSHNLSSRKLEGSLIKLTFTMCVAFVLTWTPYAVFSLWTAYGNEIPIRLTLSSILIAKLSTIVNPTVYFVLNRKFRPALKRYLSMPFNGIINLIGDKS

>Crassostrea_Opsin-5_K1QW48

--------------------AFVLNIFVIIVCVRKRSSLV-PADYFIVNLAVSDLILSVV-GLPFGISSSFLHRWTFGSGGCRIYGCLGFFCGVVSISTLALMSFSRYIHVC--KSSKS---PFFS--KHTNFFIIGSYVYACAWASFPM--LG-WGEYGVEAYGTSCTLKWT------ENRGFVTLMLISCIIFPVIIMKFCYGGVYLYLRRHCKAFNVRKREGYLIKMAFMMCCAFMLTWTPYAVVSFWAAYGDSIPVRLTLVSVLIAKTSTIWNPLIYFVLNKKFRPHIRFCLQNLFRNE-TNAQDRR

>Platynereis_Go-coupledOpsin1_AKS48306.1

DIQPRVYMVIGVYLTIAGIISTVGNSVVIGVVVKNKELRKQGHNILLLNLAICDLGFTFV-GYPLTASSAFAQRWLFGHLGCVIYGFCCTVLALTDINILMALSIYRYIVIC--KPHIR---HILHRRTVAAAMVTSCWVYSLLWGVAAL--VG-WNRYTNEAFGTSCSIDWTARGA--SDLSYTILMIFFCYISHIIVMTFCYYKIKQRSSLMLSRLNNIRNEKRLTVMTMVMVGGFILVWSPYAWVAVWKIVVPGVPDWLTTFPTMFAKATPMLNPLIYVSTNRKFRREARGMLRCC-----AKVDDIV

>Platynereis_Go-coupledOpsin2_AKS48307.1

ALGRTGYIVAGTYLCVIATVATLGNSLVVVTFVRNSSTRKKCHNILLLNLAIADLGISFF-GYPLVTVSTLSGRWMFRDYGCKIYAFCTFFFSLVSLNTLVFLSIYRYVIVC--RPSYK---HHLNK-RVTTWSIISSWIYGFFWAILPF--FG-WSHYTYEKFGTSCTIDWVDQSL--SAITYDVTVIVTCFLIHVAIMIFCYQKIIKRARNLIFDHKYMRKQSRISFMCCIMVFSFIICWTPYTVMSCVTIFT-QVPSTLSTIPTFFAKAAPMSNSIIYFFMNKKFREAFFRTFCCCATQV-EGDEDAK

>Mizuhopecten_RhodopsinG0-coupled_O15974

VISPSEFRIIGIFISICCIIGVLGNLLIIIVFAKRRSVRR-PINFFVLNLAVSDLIVALL-GYPMTAASAFSNRWIFDNIGCKIYAFLCFNSGVISIMTHAALSFCRYIIIC--QYGYR---KKITQ-TTVLRTLFSIWSFAMFWTLSPL--FG-WSSYVIEVVPVSCSVNWYGHGL--GDVSYTISVIVAVYVFPLSIIVFSYGMILQEKVCKDSRKFIQDIEQRVTFISFLMMAAFMVAWTPYAIMSALAIGSFNVENSFAALPTLFAKASCAYNPFIYAFTNANFRDTVVEIMA-------TTRRVGV

>Danio_MammalianlikeMelanopsin2_G8Z410

DVPDHAHYIIGSVILIVGITGVIGNALVVYVFCRSRTLRT-AGNMFIVNLAVADFLMSVT-QSPVFFAASLHRRWVFGERPCELYAFCGALFGICSMMTLTAIAADRCLAIT--QPLALV--SRVSR-RKAGAVLVVVWLYSLGWSLPPF--FG-WSAYVPEGLQTSCSWDYMTFTP--SVRAYTILLFVFVFFIPLGIIGSCYFAIFQTIRAAGKEIERMQNEWKMAKVALVVIVLFIISWSPYSVVALTATAGYFLTPYMNSVPAVIAKASAIHNPIIYAITHPKYRVAIARYIPVLRPIL-RVKEKDL

>Dicentrarchus_MLTReceptor_B2Y4M8

NRPPWVTTTLGCFLIFTIVVDILGNLLVIFSVYRNKKLRN-AGNIFVVSLAVADLVVAIY-PYPLVLTSIFHNGWNLGYVHCQISGFLMGVSVIGSIFNITGIAINRYCYIC--HSLKYD--KLYSD-KNSVCYVMLIWALTVV-AIVPN--LF-VGSLQYDPRVYSSTFEQS------ASSAYTIAVVFFHFILPIMIVTYCYLRIWILVIQVRRRVKLTPHDVRNFVTMFVVFVLFAVCWAPLNFIGLAVAIKPLIPEWLFVASYFMAYFNSCLNAIVYGVLNQNFRREYKRIVVSV-----DSSNDAG

>Mus_MLTReceptorO88495

DYPPALIIFMFCAMVITVVVDLIGNSMVILAVTKNKKLRN-SGNIFVASLSVADMLVAIY-PYPLMLYAMSVGGWDLSQLQCQMVGLVTGLSVVGSIFNITAIAINRYCYIC--HSLQYK--RIFSL-RNTCIYLVVTWVMTVL-AVLPN--MY-IGTIEYDPRTYTCIFNYV------NNPAFTVTIVCIHFVLPLIIVGYCYTKIWIKVLAARDPADNQFAEVRNFLTMFVIFLLFAVCWCPVNVLTVLVAVIPKIPNWLYLAAYCIAYFNSCLNAIIYGILNESFRREYWTIFHAMRARD-QVREQER

>Mizuhopecten_MLTReceptor_XP_021348996.1

EHSPVQSILFLLALSISSVTGLIGNILVIGAVIVSRRLRT-TGNMFIVNLAVADIIIVTI-VEPFNILGVIDGPEFFVRNWCHTLSVVCVMSCSSSMMNLAAISVNRYIMIN--KNHYYN--QIFTK-NKTFLLCVLVWFLAFLIELPNL--TG-WGGHTFDLKTLGCSFDRL------ISLSYTIFLSVMALWLPLLVIMFCYLNIYFYVKKSRKQMKRQRDNVNLARTFFIVFLTFLICWTPYDLTLF---FDRRWPSWLYTVFLQIGHFNSSLNSILYGATNRNFRDGYKQFLQCSRVMV-KISGDKK

>Aplysia_D1likedopaReceptor_Q29XY7

------KVLKGMFFSFCILLAISGNLLVCTAVFTERRLKRVKNNYFIVSLAVADLLVACA-VMTFALTNDVFHEWLFGPVFCHTWISFDIMCSTASILNLCVISFDRYIHIH--KALYYD--TWMTT-CKALVLIISVWILSALISILPV-HLG-WYKRTSSP----CIME--------LNFVYALVSSSISFYVPCLVMLVIY--------------------YKAAFTLGVITGVFLICWLPFFIINPIAAYD-LIPHKVFVVVTWLGYANSCCNPIIYSIFNAEYRKAFRRI----------------

>Apis_D1dopaReceptor_O44198

-------LLVGFLFLILIFLSVAGNILVCVAIYTDRGLRR-IGNLFLASLAIADLFVGCL-VMTFAGVNDLLGYWVFGPRFCDTWIAFDVMCSTASILNLCAISLDRYIHIK--DPLRYG--RWVTR-RIAVAGIAVVWLLAGLISFVPI-SLG-LHRAN-------CALD--------LTPTYAVVSSSISFYVPCIVMLGIY--------------------HKAAITVGVIMGVFLICWVPFFCVNIVTSYC-CISGRAFQVLTWLGYSNSAFNPIIYSIFNTEFREAFKRI----------------

>Architeuthis_Xeno1Candidate_VCCN01002970.1

PVTREVGLTVALVLAIFSVAAFLLNGTIISVFIIRKELIT-PTNVFIMTLSVCDFLIAIL-GNPFAIVSSVMRHWYFGRNVCIFYGFIMTFLGLTSITLITAISVDRYVLIV--HTMRT---VTIST-HISVLCVAGCATWGFFWALMPL--LG-WNGYVTEINGLACSVDWQGKTT--AAVSYIIMLLICCLFIPMLMLGFSYIRIFMKV----------------------------------------------------------------------------------------------------

>Architeuthis_Xeno2Candidate_VCCN01002970.1

GVSAEVGILVAVILSFISLVAFIQNGIFLTTFFFCRELLT-PTNMFILALSVCDLLVAGL-GNPFVVAASLYRHWYFGHSACVVYGFIMTFLGLTSITLLTAISLDRYILIV--RTMRS---VTIDC-RVALRAIGGCVLYALVWSGMPL--LG-WNEYVLEASGLACSVNWQSKTP--ASTSYIILLLIGCLFLPLVFIGFSYTRIFVRV----------------------------------------------------------------------------------------------------

>Osinensis_XenoCandidate_VCDQ01010921.1

TVSAEGGVAVATVLILFSLVAVTENGAVLFTFYICRDLIT-PTNLFILSLTVCDFCIALF-GNPFVVVSGLARGWYFGHFGCIFYGFFMTFLGLTSISLLTAISVDRYILIV--HTMRA---VTIDF-RAAIFSVIGCLLYALFWSGVPL--FG-WNLYVQEASGLACSVQWQGSTT--SDIAYIVLMLIFCLAIPLLLIGYSYIQIFIAVSETH------------------------------------------------------------------------------------------------

>Homo_DopaReceptorDRD5_P21918

PPLGPSQVVTACLLTLLIIWTLLGNVLVCAAIVRSRHLRANMTNVFIVSLAVSDLFVALL-VMPWKAVAEVAGYWPFGA-FCDVWVAFDIMCSTASILNLCVISVDRYWAIS--RPFRYK--RKMTQ-RMALVMVGLAWTLSILISFIPV-QLN-WHRDQAASNAENCDSS--------LNRTYAISSSLISFYIPVAIMIVTYTRIYRIAQVQIRRIASIKKETKVLKTLSVIMGVFVCCWLPFFILNCMVPFCSCVSETTFDVFVWFGWANSSLNPVIYAF-NADFQKVFAQLLGCSHFCS-RTPVETV

>HomodopaReceptorDRD1_P21728

ERDFSVRILTACFLSLLILSTLLGNTLVCAAVIRFRHLRSKVTNFFVISLAVSDLLVAVL-VMPWKAVAEIAGFWPFGS-FCNIWVAFDIMCSTASILNLCVISVDRYWAIS--SPFRYE--RKMTP-KAAFILISVAWTLSVLISFIPV-QLS-WHKAKPTSTIDNCDSS--------LSRTYAISSSVISFYIPVAIMIVTYTRIYRIAQKQIRRIMSFKRETKVLKTLSVIMGVFVCCWLPFFILNCILPFCGCIDSNTFDVFVWFGWANSSLNPIIYAF-NADFRKAFSTLLGCYRLCP-ATNNAIE

>Apis_DopaReceptorD1_O44198

TFSLLSVLLVGFLFLILIFLSVAGNILVCVAIYTDRGLRR-IGNLFLASLAIADLFVGCL-VMTFAGVNDLLGYWVFGPRFCDTWIAFDVMCSTASILNLCAISLDRYIHIK--DPLRYG--RWVTR-RIAVAGIAVVWLLAGLISFVPI-SLG-LHRANEPVEHPTCALD--------LTPTYAVVSSSISFYVPCIVMLGIYCRLYCYAQKHVKSIPYHVSDHKAAITVGVIMGVFLICWVPFFCVNIVTSYCKCISGRAFQVLTWLGYSNSAFNPIIYSIFNTEFREAFKRILT-------ARGNQPS

>Aplysia_DopaReceptorD1like_Q29XY7

EDDVGIKVLKGMFFSFCILLAISGNLLVCTAVFTERRLKRVKNNYFIVSLAVADLLVACA-VMTFALTNDVFHEWLFGPVFCHTWISFDIMCSTASILNLCVISFDRYIHIH--KALYYD--TWMTT-CKALVLIISVWILSALISILPV-HLG-WYKRTSSPPKPQCIME--------LNFVYALVSSSISFYVPCLVMLVIYFKLFLFARSHAVSISKRASDYKAAFTLGVITGVFLICWLPFFIINPIAAYDPLIPHKVFVVVTWLGYANSCCNPIIYSIFNAEYRKAFRRILCCT-----GRESDGV

>Strongylocentrotus_GPCR_W4Z400

TIEARW----------------------------TKSLRT-PPNMLIVNLAISDFGMVIT-NFPLMFASTIYNRWLFGDAGCQFYAFCGALFGIMSIANMTAIALDRYYVIC--WSLEAV--RSVTH-RRSMIIIIIVWCYAIFWSIPPF--FG-VGSYVLEGYGLGCTFDFMTKDL--NHYLHVSFLFASSFVVPVTIIIVCFTRIAITVRAHRHELNKAKTEFQIAKVGFQVTIFYVLSWMPYSIVAVIGQYFDLLTPLGTVVPVIFAKCSAIWNPIIYCLSHEKFNAALKEKLMCGHRSM-GSQESSV

>brachionus_rhabdomericopsin_A0A0A7DNA7

PIERKWHFLIAFIYFVIGVVGFFTNFMVFYYLIRIKNVKR-PGTYFLINLAIADIGKILA-CLPMNAVSSFNAKWSFGQIGCDIYGLAGGLFGFVSITTMVFMSLERYLMVK--NPLFA---LKISN-RTVFICILITWIYSSACIFPQL--TL-KHGFVLEGLLTSCTFDYLDRDI--YSRLFMMILFVAGFLVPNEITIMWMCQLMSVKRVQNQEVTFVKRELKVAKTVVVIVAMFCIAWLPYAIITLIAQYISFINPLTTSLPAIFAKTSSIYNPILYTLSNKDCKNYFRKLFVVRILKL-KKNKEVS

1. **Cryptochromes tree**

>Sof_CRY6_MN788454

HWFRHGQRIHDNPALVDAVNDSDEFYPIFIFDGKVAGTELCGYNRWRFLLENLKDLDESFSKFGGKLYCFHGQPVDIFQKLFKEWNVNYITAEEDPEPIWQERDESVKKLCEKSGVTCKFFTSHTLYSPQEIIDKNGSTPPLTLELFQLVITSLGPPLRPLPPTLDFFGIEPEFEEGGETRALNLLKARLAVSLSPYLRFGCVSIRRTYWGICD-MYREVH-NKEPPSEVVCQLYWREYFYVMSVGNLNYDKMEGNPICEHLKKWELGQTGFPWIDAIMNQLRFEGWNHHVGRHSVSCFLTRGDLWISWEDGLKVFLKYQLDADWSVCAGNWMWVSSSALQCPTCYSPVMYGMRMDRNGEFVRMYVPVLKDMPLKYLFCPWKAPLEIQKNANCIIGKDYPEP

>Sof_CRY123_MN788455

HWFRKGLRLHDNPALKEALKGANTIRCIYTLDPWFAGSSQVGINKWRFLLNCLEDLDASLRKLNSRLFVVRGQPADVFPQLFKEWNITTLSFEEDPEPFGKDRDAAICTLVRDAGIEVIIKTCHTLYDSKAILEKNGGRPPLTYNKFQKILAEMDLPPRPLEPTLEELGFDTEGLGGGETEALARLDRHLHTALSPYLRFSCLSPRLFYWKLTE-LYTKIKKKSNPPLSLHGQLLWREFFYTAASNNPRFDQMVGNPVCEALAKWAEGMTGFPWIDAIMKQLRKEGWIHHLARHSVACFLTRGDLWISWEDGMKVFEELLLDADWSVNAGMWMWLSCSSQQFFHCYCPVGFGKRIDPNGDFIRHYLPILKRFPAKYIYEPWNAPESVQKTAKCIIGKDYPVP

>Dorytheutis_putativeCRY123_comp136709_c1_seq2

HWFRKGLRLHDNPALKEALKGANTIRCVYTLDPWFAGSSQVGINKWRFLLNCLEDLDAGLRKLNSRLFVVRGQPADVFPQLFKEWNITTLSFEEDPEPFGKDRDAAICTLVRDAGIEVIIKTCHTLYDSKAILEKNGGRPPLTYNKFQKILADMDLPPRPLEPTLEELGFDTEGLGGGETEALARLDRHLHTALSPYLRFSCLSPRLFYWKLTE-LYTKIKKKSNPPLSLHGQLLWREFFYTAASNNPRFDQMVGNPVCEALAKWAEGMTGFPWIDAIMKQLRKEGWIHHLARHSVACFLTRGDLWISWEDGMRVFEELLLDADWSVNAGMWMWLSCSSQQFFHCYCPVGFGKRIDPNGDFIRHYLPILKRFPAKYIYEPWNAPESVQKTAKCIIGKDYPVP

>Dorytheutis_putativeCRY6_comp135755_c0_seq1

HWFRHGQRIHDNPALVDAVKNSDEFYPIFIFDGKVAGTEICGYNRWRFLLENLKDLDESFSQFGGRLYCFHGQPENVFQNLFKEWNVNYVTAEEDPEPIWKERDDGIKKLCEKSGVTCKFFTSHTLYSPDKIIAKNGGTPPLTLELFQLVITSLGRPLRPMDPNLSYFGIEPEFEEGGEKRALKLLKARLAVSLSPYLRFGCVSIRTTYWGICD-TYMKVH-KKEPPSEVVCQLYWREYFYVMSIGNINFDKIEGNKICEMLKKWEFGQTGYPWIDAIMNQLRFEGWNHHVGRHAVSCFLTRGDLWISWEDGLKVFLKYQLDADWSVCAGNWMWVSSSALQCPTCYSPVMYGMRMDRNGEFVRAYVPVLKDMPLKYLFCPWKAPLEVQKEANCIIGKDYPEP

>Euprymna_Cry1_AGJ94014.1

HWFRRGQRIHDNPALIDALKDCDEFYPIFIFDGKVAGTEICGYNRWRFLLENLKDLDDTFSQFGGRLYCFHGQPVDIFKNMFEEWGVNYITAEEDPEPIWKERDDSARELCEESGITCKFFTSHTLYSPQDIISKNGGTPPLTLELFQLVISSLGDPMRPIPPNLSYFGIEPECEEGGEKRALALLKARLAISLSPYLRFGCVSIRKTYWGICD-TYKQVC-HKETPSEVICQLHWREYFYVMCVGNINFDRIEGNPICELLKKWEFGQTGYPWIDAIMNQLRFEGWNHHVGRHAVSCFLTRGDLWISWEEGLKVFLKYQLDADWSVCAGNWMWVSSSALQCPTCYSPIMYGMRMDRNGEFVRTYLPVLKDMPLKYLFCPWKAPLGVQEKANCIIGKDYPEP

>Ovulgaris_Cry1like_XP_029634730.1

HWFRHGQRLHDNPALVNALKDCDEFYPVFIFDGEVAGTKLCGFNRWRFLLENLKDLDESFAEYGGRLYTFQGDPIDVFKNLQKEWGITHITAEIDPEPIWLERDDAVKKFCQKSGIECDFFTSHTLWDPQLLLKKNGGTPPLTFELFQLVTSSLGPPLRPLDPTLKSLGIHPEFEEGGEKRALVLLKARLAVSLSPYLRFGCVSIRKTYWDICD-TYKKIK-NVEAPNEIVCQLYWREYFYIMSIDNINFDKIENNPYCEFLKKWEMGQTGYPWIDAIMNQLRFEGWNHHVGRHAVSCFLTRGDLWVSWEDGLKTFLKYQLDADWSVCAGNWMWVSSSALQCPTCYSPVMYGMRMDRNGDFVKTYVPVLKDMPLKYLFCPWKAPLEIQEKANCIIGKDYPEP

>Osinensis_putativeCRY6_VCDQ01000004.1

HWFRHGQRLHDNPALVNALKDCDEFYPVFIFDGEVAGTKLCGFNRWRFLLENLKDLDESFAEYGGRLYTFQGDPIDVFKNLQKEWGITHITAEIDPEPIWLERDDAVKKFCQKSGIECDFFTSHTLWDPQLLLKKNGGTPPLTFELFQLVTSSLGPPLRPLDPTLKSLGIHPEFEEGGEKRALVLLKARLAVSLSPYLRFGCVSIRKTYWDICD-TYKKIK-NVEAPNEIVCQLYWREYFYIMSIDNINFDKIENNPYCEFLKKWEMGQTGYPWIDAIMNQLRFEGWNHHVGRHAVSCFLTRGDLWVSWEDGLKTFLKYQLDADWSVCAGNWMWVSSSALQCPTCYSPVMYGMRMDRNGDFVKTYVPVLKDMPLKYLFCPWKAPLEIQEKANCIIGKDYPEP

>Architeuthis_putativeCRY6_VCCN01002226.1

------------------------------------------------------------------------------------------------------------------------------------------------------------------PTLSSLGIEPEFEEGGEKRALKTLEARLNDILSPYLRFGCVSIRTTYWGVCD-MFMKVH-NKEPPNEVICQLYWREYFYVMSVGNINFDRIEDNPICELLKKWELGQTGYPWIDAIMNQLRFEGWNHHVGRHAVSCFLTRGDLWISWEDGLKVFLKYQLDADWSVCAGNWMWVSSSALQCPTCYSPVMYGMRMDRNGEFVRAYVPILKDMPLKYLFCPWKAPLEIQKQANCIIGKDYPEP

>Ovulgaris_Cry1likePREDICTED_XR_003882494.1

HWFRKGLRLHDNPALMEALKGATTLRCIYTLDPWFAGSSQVGINKWGFLLNCLEDLDDSLRKLNSRLFVIRGQPANMFPQLFKEWNINTLSFEDDPEPFGKDRDNAICTLAREAGVEVIIRTCHTLYESQKILDLNGGRPPLTYNKFQKILENMKMPPKPLDPTLEDLGFDTEGLDGGESEALARLDRHLHTALSPYLRFSCLSPRLFYWKLTE-LYRKIKKKNIPPLSLHGQLLWREFFYTVASNNPRFDQMLGNPVCEALAKWAEGMTGFPWIDAIMKQLRKEGWIHHLARHSVACFLTRGDLWLSWEEGMKVFDELLLDADWSVNAGMWMWLSCSSQQFFHCYCPVGFGKRIDPNGDFIRHYLPILKRFPAKYIYEPWNAPESVQKTAKCIVGKDYPIP

>Obimaculoides_Cry1likePREDICTED_XM_014912568.1

HWFRKGLRLHDNPALMEALKGATTLRCIYTLDPWFAGSSQVGINKWGFLLNCLEDLDDSLRKLNSRLFVIRGQPANMFPQLFKEWNINTLSFEDDPEPFGKDRDNAICTLAREAGVEVIIRTCHTLYESQKILDLNGGRPPLTYNKFQKILENMKMPPKPLDPTLEDLGFETEGLDGGESEALARLDRHL--------------------------------------------------------------------------------------------------------------------------------------------------------------------------------------------------------------------

>Obimaculoides_Cry1like_XP_014774572.1

HWFRHGQRLHDNPALVNALKDCDEFYPVFIFDGEVAGTKLCGFNRWRFLLENLKDLDESFAEYGGRLYTFQGKPVEVFKNLQKEWGITHITAEIDPEPIWQERDDAVKEFCQKSGIECDFFTSHTLWDPKLLLKKNGGTPPLTFELFQLVTSSLGPPLRPLDPTLKSLGIDPEFEEGGEKRALVLLKARLAVSLSPYLRFGCVSIRKTYWDICD-TYKRIK-KVEAPNEIVCQLYWREYFYIMSIDNINFDKIENNPYCEFLKKWEMGQTGYPWIDAIMNQLRFEGWNHHVGRHAVSCFLTRGDLWVSWEDGLKLFLKYQLDADWSVCAGNWMWVSSSALQCPTCYSPVVYGMRMDRNGDFVKTYVPVLKDMPLKYLFCPWKAPLEIQEKANCIIGKDYPEP

>Cgigas_Cry1_ANJ02841.1

HWFRHGLRLHDNPSLIDGLSECDRFYPVFIFDGEVAGTKTAGYNRFRFLLECLQDLDKNLKAAGTRLYCFQGQPTDILERLIEEWGVTKVTFEADPEPIWQERDRLVRELLDKKNVQCVEKVSHTLWDPYEIIENNGGSPPLTFSLFNLVTSTIGPPPRPVEPSLEDLNVRPECEEGGESKALELLAIRMPLSLSAHLRFGCLSVRKFYWSIHD-KFEEVKPSMGAPVSLSAQLMWREYFYTMAINNINYDKMETNPICEHEEKWTQGETGYPWIDAIMKQLRYEGWVHHVARHAVSCFLTRGDLWLNWEVGLKVFYKYLLDADWSVCAGNWMWVSSSALQCPNCFCPVRYGKRMDPSGEYVRRYLPVLKDMPLRYLFEPWKAPLPVQQKAKCIVGVDYPKP

>Cvirginica_Cry1like_XP_022291740.1

HWFRHGLRLHDNPALIEGLAECDKFYPVFIFDGEVAGTKTAGYNRYRFLLECLRDLDDNLQAAGTRLYCFQGQPTDIFERLIEEWGVTKVTFEADPEPIWQERDRLVKDLLDKRNVQCVERVSHTLWDPHEIIQNNGGNPPLTFSLFNLVTSTIGVPPRPVEPSLEDLNIRPECEEGGETKALELLEIRMPLSLSAHLRFGCLSVRNFYWRIHD-KFKEVKPSVQALVSLSAQLLWREYFYTMAVNNMNYDKMENNPICEHEEKWTKGETGYPWIDAIMKQLRYEGWIHHVARHAVSCFLTRGDLWLSWEVGLKVFYKYLLDADWSVCAGNWMWVSSSALQCPNCFCPVRYGKRMDPSGEYIKRYLPVLKDMPLRYLFEPWKAPLPVQQKAKCIVGVDYPRP

>Mizuhopecten_Cry1like_XP_021380007.1

HWFRNGLRIHDNPALCDALENCDEFYPIFIFDGEVAGTKFSGFNRMRFLHESLEDLDNTFKKNGGRLYTFQGKAKDVLKGLFQEWNVTRLTYEAEVEPIWEDRDMEVEELCQEKGIELISRISHTLWNPSDIINNNGGTPPVTYNHFELTTSLLGPPDRPVPPSVKELGYEPECKEGGETKALQLLEERLPTSMSPHLRFGCVSVRTFYWGIHD-LYKEIHGDKKVPVSLTGQLVWREYFYTMSVNNLNYDKMKANPICEKLQSWVMGKTGYPWIDAGMNQLRTEGWCHHVVRHAVSCFLTRGDLWISWELGCKVFFKYQLDADWSVCAGNWMWMSSSALQCSTCFCPVGYGRRMDPKGLYVRRYVPVLKDMPLQYLFEPWKAPYHIQEKAKCIIGKDYPRP

>Pomacea_Cry1like_XP_025095473.1

HWFRHGLRLHDNPALLESLKNADEFYAVFIFDGSVAGTATAAYPRMRFLLECLSDLDAGLRKLGTRLYIIHGQPEEVFPRLFEEWGVSRVTFEQDPEPVWQDRDNKVKTLCKQWKVECIEKVSHTLWDPQSIIQANGGSPPLTYAMFCQVTDIVGLPPRPCPPTCEELGVYPESERGGEQRALELLSLRIPLSLSPHLRFGSLSVRRFYWAIHD-AFKEVSQCEEVPTSITGQLIWREYFYCMSVNNPMYNCMEGNPICEQFEKWSKGQTGYPWIDACMRQLKEEGWIHHVCRHAVACFLTRGDLWIDWQMGLKVFDKYLVDADWSVCAGNWMWVSSSALQCPKCICPVRYGRRMDPKGDYVRRYVPELKNMPLLYLFEPWKAPAEIQEKAGCIIGCDYPPP

>Biomphalaria_Cry1like_XP_013075939.1

HWFRHGLRLHDNPALLDGLEDCVEFYPIFIFDGNVAGISTAAFPRMQFLLETLQDLDENLKSHGSRLYSFQGDPVEIFKKLIEEWGVTRITFEQDPEPVWQDRDTRVKNMCLELEIECIERVSHTLWDPQQIIKENGGTPPLTYAMFCQVADIVGPPPKPREPTLAELGVEAECEKGGESKAMKLLSIRLPMSLSPHLRFGSMSIRKFYWLLRE-AYAEVHPNAHIPSSITGQLIWREYFYCMSVNNPNYNRMVGNPICNHLKAWKMGMTGYPWIDACMRQLLQEGWIHQVCRHATACFLTRGDLWIDWVKGLQVFDRYLLDADWSVCAGNWMWVSSSALQCPKCICPVRYGRRMDPKGEYVRRYVPELKDMPLQYLFEPWKAPLKVQEEAKCIIGEDYPLP

>Aplysia_Cry1like_XP_005089742.1

HWFRHGLRLHDNPALLDSLENCREFYPVFILDGNVAGISSAAFPRMQFLFETLQDLDDNLRSHGSRLFVLRGKPVDVFAKMFEEWGVTRLTFEQDPEPVWQERDNSVKALCDKQNVEWIERVSHMLWEPRLILEENGGEPPLTYAMFNQVAQVVGPPPKPVGPSLEEIDIRPESEEGGETKALKLLSSRLPMSLSPHLRFGSLSVRKFYWSLRD-TFSQLFPDRPVPGSITSQLVWREYFYCMSVNNPMYNRMKENPICQKFEKWKKGQTGFPWIDACMRQLLQEGWIHQVCRHAVACFLTRGDLWIDWQKGLKVFDRYLLDADWSVCAGNWMWVSSSALQCPRCICPVRYGKRIDPTGAYVRRYVPELKRMPLQYLFEPWKAPLKLQEEVDCIIGKDYPEP

>Hermissenda_photoreceptiveCry_AWY10935.1

HWFRHGLRLHDNPALLEGLENCQEFYPIFIFDGSVAGTKTAGYCRMQFLLESLKDLDDSLRAKGSRLYVMEGDPLQIFPNLFEEWKVNMLTFEQDPEPIWQERDDNVKDLCDSYNVEWIERVSHTLWDPHSILKANGGSPPLTYAMFCQVAEIVGQPPRPVEPTLEKLEVVADCDAGGEAKALVLLESRLPMSLSPHLRFGSLSIRKFYWALRD-AFAEIHPESEIPLQVTSQLVWREYFYCMSVNNPFYNRMAENPICTSLEKWTEGKTGYPFIDACMRQLKQEGWIHQVCRHAVSCFLTRGDLWIDWQKGLEVFDRYLLDADWSVCAGNWMWVSSSALQCSRCICPVRYGRRMDPNGDYIRRYVPELKNIPLTYLYEPWKAPIAVQEGANCIIGTDYPPP

>Tritonia_photoreceptiveCry_AWY11208.1

HWFRHGLRLHDNPALLEGLENCQEFYPVFIFDGSVAGTKNAGFSRMQFLLETLKDLDNNLKAKGSRLYVFKGDPEQVFKNIFEEWKVTRLTFEQDPEPIWQDRDNNVKDLCESLNVEWIEKVSHMLWDPHLIIQENGDTPPLTYAMFCQVAEIVGLPPRPVEPTMEDLGVQAECAAGGETKALMLLDLRLPMSLSPHLRFGNLSIRKFYWALRD-AYSEVNPSTCIPPEITSQLIWREYFYCMSVNNPYYNRMKENPICQGFEKWKDGKTGYPWIDACMRQLIQEGWIHQVCRHAVSCFLTRGDLWIDWQKGLEVFDRHLLDADWSVCAGNWMWVSSSALQCPRCICPVRYGRRIDPTGDYIRRYVPELKNMPLDYLFEPWKAPKDVQEKAGCVIGTNYPPP

>Melibe_photoreceptiveCry_AWY11207.1

HWFRHGLRLHDNPSLLEGLENCSEFYPVFIFDGSVAGTKTAGYSRMQFLLETLRDLDENLKKKGSRLYVFMGEPVKIFRQLFEDWNATRLSFEQDPEPIWQDRDNNVKDLCDACNVEWIERVSHMLWDPHVILRANGGSPPLTYAMFCQVTEIVGLPPRPVEPSLEKLGIVAECEAGGETKAAALLVSRLPMSLSPHLRFGSLSIRKLYWALRD-TYSELYPGSSVPMSVTSQLIWREYFYCMSVNNPNYNRMFENPICALLRKWKEGKTGYPWIDACMRQLVQEGWIHQVCRHAVACFLTRGYLWIDWQKGLETFDHYLLDADWSVCAGNWMWVSSSALQCPRCICPVRYGKRMDPTGTYVRRYVPELKNMPMNYLFEPWKAPESVQLEADCIIGTDYPSP

>Cgigas_Crylike_ACV53158.1

-----GLRLHDNPSLIDGLSECDRFYPVFIFDGEVAGTKTAGYNRFRFLLECLQDLDKNLKAAGTRLYCFQGQPTDILERLIEEWGVTKVTFEADPEPIWQERDRLVRELLDKKNVQCVEKVSHTLWDPYEIIENNGGSPPLTFSLFNLVTSTIGPPPRPVEPSLEDLNVRPECEEGGESKALELLAIRMPLSLSAHLRFGCLSVRKFYWSIHD-KFEEVKPSMGAPVSLSAQLMWREYFYTMAINNINYDKMETNPICEHEEKWTQGETGYPWIDAIMKQLRYEGWVHHVARHAVSCFLTRGDLWLNWEVGLKVFYKYLLDADWSVCAGNWMWLS------------------------------------------------------------------

>Pomacea_Cry1like_XP_025096987.1

HWFRKGLRLHDNPALLEALKDSSSYRCVYILDPWFAGSSQVGINKWRFLLESLEDLDSSLRKLNSRLFVIRGQPADVFSRIFKEWNITQLTFEEDPEPYGKERDAAICALAAEIGVQVVTKPSHTLYDLRHILEANNNQPPLTYRRFQSILSGLLPPPVPLEPSLEELGFDTDNLGGGESEALARIYRHLQTGLSPYLRFGCLSPRLFYWKLTE-LYKKVKKGQEPPLALHGQLLWREFFYTAATNNPNFDKMSGNSICEALAKWAEGQTGFPWIDAIMVQLKKEGWIHHLARHAVACFLTRGDLWISWEEGMKVFDELLLDADWSVNAGMWMWLSCSAQQFFHCYCPVNFGKRADPTGDFVRHYLPALKGFPTQYIYEPWTAPEAVQKAARCIIGKDYPLP

>Aplysia_Cry1like_XP_012941094.1

FWFRKGLRIHDNPALIAAIEGASTYRCVYILDPWFAGASQVGINKWRFLLESLEDLDSSLRKLNSRLFVVRGQPADVLPRLFQEWAITCLAFEEDPEPYGKERDSAISAMAREFNVQVIAKSSHTLYDPKLVIAANGNSPPLTYKRFQSVLSTLEPPNQPCEPSLDELGFDTDTLDGGESEAVARLHRHLGTGLSPYLRFGCLSARTFYWKLTE-LYKKVKKGAEPPLALHGQLLWREFFYTVATNNPNFDRMVGNSICEALVKWAEGMTGFPWIDAIMVQLKKEGWIHHLARHAVACFLTRGDLWISWEEGMKVFDEMLLDADWSVNAGMWMWLSCSAQQFFHCYCPVGFGKRADPSGDFVRHYLPVLKAMPTKYIYEPWTAPESVQKVAKCIVGKDYPMP

>Cgigas_Cry1like_XP_011455127.1

HWFRKGLRLHDNPSLREALKGSSSYRCVYILDPWFAGSSQVGINKWRFLLQCLEDLDTSLRKLNSRLFVLRGQPTDLFPKIFKEWNITTLSFEEDPEPFGKERDGAIQMLAKEAGVEVIVKTSHTLYDLQKIIAMNGGSPPLTYKRFQSVLAKMEAPSEPEEPTLEELGFDTEGLGGGEAEALTRLERHLQNVLSPYLRFGCLSARLFYWKLRE-LYRKVKKRKDPPLSLHGQLLWREFFYTVATNNPNFDRMKDNPLCEALAKWAEGKTGFPWIDAIMMQLRQTGWIHNLARHSVACFLTRGDLWISWEEGMKVFEELLLDADWSVNAGMWMWLSCSSQQFFHCYCPVGFGKRADPTGDFIRTYLPVLKGYPAKYIYEPWTAPESVQRAAKCIIGKDYPVP

>Onchidium_Cry1like_QDK59985.1

FWFRKGLRLHDNPALIAAIEGASTYRCVYILDPWFAGASQVGINKWRFLLESLEDLDSSLRKLNSRLFVVRGQPADVLPRLFQEWGITTLAFEEDPEPYGKERDAAISAMAREFNIQVIAKSSHTLYDPKIVIAANGNSPPLTYKRFQSILSTLEPPHQPCEPSLDELGFDTDSLGGGESEALARLYRHLGTGLSPYLRFGCLSPRTFYWKLTE-LYKKVKKGADPPLALHGQLLWREFFYTVATNNPNFDRMVGNSICEALAKWAEGMTGYPWIDAIMVQLRKEGWIHHLARHAVACFLTRGDLWISWEEGMKVFDEMLLDADWSVNAGMWMWLSCSAQQFFHCYCPVGFGKRADPSGDFVRHYLPVLKGIPTQYIYEPWTAPESVQKAAKCIIGKDYPLP

>Cgigas_Cry2_AQM57602.1

HWFRKGLRLHDNPSLREALKGSSSYRCVYILDPWFAGSSQVGINKWRFLLQCLEDLDTSLRKLNSRLFVLRGQPTDLFPKIFKEWNITTLSFEEDPEPFGKERDGAIQMLAKEAGVEVIVKTSHTLYDLQKIIAMNGGSPPLTYKRFQSVLAKMEAPSEPEEPTLEELGFDTEGLGGGEAEALTRLERHLQNVLSPYLRFGCLSARLFYWKLRE-LYRKVKKRKDPPLSLHGQLLWREFFYTVATNNPNFDRMKDNPLCEALAKWAEGKTGFPWIDAIMMQLRQTGWIHNLARHSVACFLTRGDLWISWEEGMKVFEELLLDADWSVNAGMWMWLSCSSQQFFHCYCPVGFGKRADPTGDFIRTYLPVLKGYPAKYIYEPWTAPESVQRAAKCIIGEDCPVP

>Melibe_NONphotoreceptiveCry_AWY10933.1

FWFRKGLRLHDNPALYSAIDGAVTYRCVYILDPWFAGASQVGINKWRFLLESLEDLDNSLRKLNSRLFVARGQPADVLPRLFQEWRITTLAFEEDSEPYGKERDSAISAMAREFNIQVISKSSHTLYNPKTVIAANGNSPPLTYKRFQSILSTLDPPEQPCDPTLDELGFDTDSLGGGESEALARLHRHLGTGLSPYLRFGCLSPKTFYWKLTE-LYKKVKKGIDPPLALHGQLLWREFFYTVSTNNPKFDRMVGNSICEALAKWAEGMTGYPWIDAIMIQLRKEGWIHHLARHAVACFLTRGDLWISWEEGMKVFDEMLLDADWSVNAGTWMWLSCSAQQFFHCYCPVGFGKRADPSGDFVRQYLPVLKNMPTRYIYEPWTAPESVQKAAKCIVGKDYPLP

>Tritonia_NONphotoreceptiveCry_AWY10934.1

FWFRKGLRLHDNPALFNAIEGALTYRCVYILDPWFAGASQVGINKWRFLLESLEDLDSSLRKLNSRLFVARGQPADVLPRLFQEWGITTLAFEEDSEPYGKERDAAISTMAREFHIQVISKSSHTLYNPRTVIAANGNNPPLTYKRFQSILSTLDPPDQPYEPTLDELGFDTDSLGGGESEALARLHRHLGTGLSPYLRFGCLSPKTFYWKLTE-LYKKVKKGIDPPLALHGQLLWREFFYTVSTNNPKFDRMVGNTICEALAKWAEGMTGFPWIDAIMVQLRKEGWIHHLARHAVACFLTRGDLWISWEEGMKVFDEMLLDADWSVNAGMWMWLSCSAQQFFHCYCPVGFGKRADPSGDFVRQYLPVLKNMPTQYIYEPWTAPESVQKAAKCIVGKDYPLP

>Euprymna_Cry2_AGJ94015.1

HWFRKGLRLHDNPALKEALEGATTIRCVYTLDPWFAGSSQVGINKWRFLLNCLEDLDASLRKLNSRLFVVRGQPADVFPQLSKEWNITTLSFEEDPEPFGKDRDAAICTLVRDAGIEVIIKTCHTVYDSKAILEKNGGRPPLTYNKFQKILEEMDLPPRPLEPTLEELGFDTEGLGGGETEALARLDRHLHTALSPYLRFSCLSPRLFYWK?NRTI??DQKGRTIPPLSLHGQLLWREFFYTAASNNPRFDQMVGNPVCEALAKWAEGMTGFPWIDAIMKQLRKEGWIHHLARHSVACFLTRGDLWISWEDGMKVFEELLLDADWSVNAGMWMWLSCSSQQFFHCYCPVGFGKRIDPNGDFIRHYLPILKRFPAKYIYEPWNAPESVQKTAKCIIGKDYPVP

>Tritonia_6-4_photolyase_AWY11211.1

HWFRKGLRLHDNPAFIKACEQASEIYPIFILDPWFAKNCRVGINRWRFLLQALRDLDNSLKTLNLRLFVVRGNPNTELENLCQRWKISRLTFEVDTEPYAVKRDEEVERQMGEMGIEVIKSLSHTLFEPQRVIKVNGGSAPLTYQRFQTIASKLGPPPKPLDPTLTELGKDPKSCGGGEKEALERMERQLTTVLSPYLKFGCLSSRLFYHQLAD-MYKGKKH-SQPPVSLHGQLLWREFFYTVASVTPNFDKMEGNPVCDHLEKWANGQTGFPFIDAIMRQLRQEGWIHHLARHAVACFLTRGDLWISWEEGMTVFEELLLDADWSLNAANWMWLSASAHQYFRVYSPIAFGKKTDPNGDYIRKYVPEVKKFPSKYIFEPWTAPLSMQQTAGCVLGKDYPKP

>Melibe_CryDASH_AWY11213.1

VLFRNDLRIHDNEAIFLANKNCSQVLPLYCFDPRHFQFPKTGSHRAKFLLECIADLKANLQRKGSDLVVRLGKPENVIPELMTNLNLGTDTMVVMQKEVTDEELKVEKALKEKSKSKFETVWGHTLHHKED-LPFQIKHLPDVYTQFRKKVEGNTPIRKCFQPTFQDLHLSEPGRNGGESAALERLENYLSTKFSSWLAHGCISPRKIYWEIQK--YEQERTANQSTYWVIFELLWRDYFRLVAMK-------FGNQLFLHFKAWQEGKTGVPYVDANMRELAASGFMSNRGRQNVASFLTK-DLELDWRLGAEWFESMLIDHDVCSNYGNWLYSAGIGPRENRKFNVVKQGLDYDGQGEYIRLWVPELKEVKDGRVHCIWTASKTILDSSGVSLGETYPYP

>Pomacea_CryDash_XP_025105331.1

YLFRNDLRLHDNESLYWACQNADTVLPVYCFDPRHFGFAKTGPHRLKFLVESVVDLRGNLQKHGSDLMVRQGKPEDVIPDIISQLGKDNVEALVYQKEVTQEELDVEEALKLKSGVKVHTVWGHTMVHIDD-LPFKPQTLPNAYTQFRKKVEESNRMRKELPPTFDDFKIPMSVPVGGETSALARLQSYFSTKFSPWLAHGCISPRRIYWEIKR--YEQQRTANESTYWVLFELLWRDYFRFVALK-------YGNKIFKLFEAWREGRTGVPYIDANMRELQATGFMSNRGRQNAASFLIK-DLQIDWRIGAEWFESILIDHDVCSNYGNWLYIAGIGPRENRKFNVIKQGLDYDANGDYVRLWVPELSKVKRGSVHVVWTLSPAALSSAQVSIGESYPAP

>Hermissanda_CryDASH_AWY11212.1

VLYRNDLRIHDNEALFLANKNHSQVLPVYCFDPRHFQFPKTGRHRAKFLLESVADLKSSLQSRGSDLVVRLGKPEDVIPELITNLKLGKDTLVLLQKEVTDEEVQVEKALQSSVGVTVSTVWGHTLHHLDD-LPFQPRNLPDIYTQFRKSVEDNTPVRKCFQPTIQKLGLSEPEPHGGESTALSRLHHYLSTKFSSWLAHGCISPRQIYWDIKK--YESERTANQSTYWVIFELLWRDYFRLVALK-------FGNKIFLQFKAWQEGHTGVPYIDANMRELEATGFMSNRGRQNVASFLTK-DLLLDWRLGAEWFESMLIDHDVCSNYGNWLYSAGIGPRENRKFNVVKQGLDYDADGAYVRTWVPELSKVKSGQVHCVWTISQMGLDAAGVSLGETYPHP

>Tritonia_CryDASH_AWY11214.1

VLFRNDLRLHDNEALFLANKNCSHVLPLYCFDPRHFQLPKTGSHRAKFLLECVTDLKTNLQSRGSDLVVRLGKPENVIPELMANFNLGQDTVVVFQREVTDEEVKVEKGVKENSKLRVETVWGHTLHHVAD-LPFQPKDLPDVYTEFRKRVEGNTAVRQCFNLTLQDLGLSVPETDGGETAALERLNNYLSTKFSSWLAHGCISPRKIYWEIKK--YEQERTANQSTYWVIFELLWRDYFRYVAMK-------FGNQLFQHFKAWQEGKTGVPYVDANMRELAATGFMSNRGRQNVASFLTK-DLQLDWRLGAEWFESMLIDHDVCSNYGNWLYSAGIGPRENRKFNVVKQGLDYDADGDYIRIWVPELRGLKSGKVHCIWTASKMIHDSAGISLGETYPYP

>Aplysia_CryDASHlike_XP_005098341.1

YLFRNDLRVHDNEALLLANQKGTRLLPLYCFDPRHYSFPKTGNHRLSFLLDSVKDLQKNLKSRGSDLVIRKGKPEEVIPDLLRTLNVTRDAAVVFQKEVMEEEVKVEKAIEAHVNVPVNTTWGHTLYHVED-LPFQPIRLPDVYTQFRKKVEDNTTIRKCVHPSYEDLGVSEPEKDGGETTALERLHSYLSTKFSPWLAHGCLSPRKIYWEIKK--YEKERTSNQSTYWVIFELIWRDYFRFVGLK-------YGNKLFEQFKAWQEGRTGVPYVDANMRELMATGFMSNRGRQNVASFLTK-DLHLDWRLGAEWFESMLIDHDVCSNYGNWLYSAGIGPRENRKFNVVKQGLDYDAEGDYVRLWVPELAGIKTGSVHCVWTLSPAVLESGDVSLGQTYPLP

>Melibe_6-4photolyase_AWY11210.1

HWFRKGLRLHDNPALLKAFEHASEVYPIFVLDPWFVSSCKVGINRWRFLLEALKDLDDKLKALNLRLFVVRGSPDKELEKLCKKWNISRLTFEVDTEPYAVKRDEQVEAQMKNLGVEVIKCTSHTLFDTAKVVKTNKGEAPLSYRGFQTILSKLGAPPKPLDPTLNELGKDAESCGGGETEALERMERHLYFS-----------------------------------------------------------------------------------------------------------------------------------------------------------------------------------------------------------------

>Hermissanda_6-4photolyase_AWY11209.1

HWFRKGLRLHDNPGFLKACEQASEIYPVFILDPWFLKHCRVGSNRWRFLFQALEDLDNSLKSLNLRLFVVRGNPCRQLEILCKKWKISRVTFEVDTEPYAVTRDEDIERRLTELGIEVHKCVSHTLFDTAKVVKANGGSPPLTYQRFQTVLSK---------------------------------------------------------------------------------------------------------------------------------------------------------------------------------------------------------------------------------------------------------

>Anopheles_Cry2_ABB29887.1

HWFRKGLRLHDNPALREGLRGARTFRCVFIIDPWFAGSSNVGINKWRFLLQCLDDLDRNLRKLNSRLFVIRGQPADALPKLFKEWGTTCLTFEEDPEPFGRVRDHNISEMCKELGIEVISAASHTLYNLERIIEKNGGRAPLTYHQFQAIIASMDAPPQPEAPTLEELGFETEALRGGETEALARLERHLQTGLSPYLRFGCLSTRLFYYQLTD-LYKKIKK-ACPPLSLHGQLLWREFFYCAATKNPTFDKMAGNPICEALAKWASGQTGFPWIDAIMTQLREEGWIHHLARHAVACFLTRGDLWISWEEGMKVFEELLLDADWSVNAGMWMWLSCSSQQFFHCYCPVKFGRKADPNGDYIRRYLPVLKNFPTRFIHEPWNASESVQRAAKCLIGKDYPLP

>Anopheles_Cry1_ABB29886.1

LWFRHGLRLHDNPSLLEALKEAVKLFPIFIFDGESAGTRIVGYNRMKFLLESLADLDRQFRDLGGQLLVFRGDSVTVLRRLFEELNIKKLCYEQDCEPIWKERDDAVAKLCRTMDVRCVENVSHTLWNPIEVIQTNGDIPPLTYQMFLHTVNIIGDPPRPVGPAPDDFGIHYDGNAGGETRALEALGARLATSMSAALRFGCLSVRMFYWCVHD-LFAKVQSNSQFKHHITGQLIWREYFYTMSVQNPHYGEMERNPICDSLTRWKEGRTGFPMIDAAMRQLLAEGWLHHILRNITATFLTRGGLWLSWEEGLQHFLKYLLDADWSVCAGNWMWVSSSALDSSKCTCPIALARRLDPKGDYVKRYLPELANYPAQFVHEPWKASREQQIEYGCVIGEKYPAP

>Danio_Cry1a_NP_001070765.2

HWFRKGLRLHDNPSLRDSILGAHSVRCVYILDPWFAGSSNVGISRWRFLLQCLEDLDASLRKLNSRLFVIRGQPTDVFPRLFKEWNINRLSYEYDSEPFGKERDAAIKKLANEAGVEVIVRISHTLYDLDKIIELNGGQSPLTYKRFQTLISRMEAVETPAEPSLEELGFDTEGLSGGETEALTRLERHLPTGLSPYLRFGCLSCRLFYFKLTD-LYRKVKKNSSPPLSLYGQLLWREFFYTAATNNPRFDKMEGNPICEALAKWAEGRTGFPWIDAIMTQLRQEGWIHHLARHAVACFLTRGDLWISWEEGMKVFEELLLDADWSVNAGSWMWLSCSSQQFFHCYCPVSFGRRTDPNGDYIRRYLPVLRGFPAKYIYDPWNAPESVQKAAKCIIGVHYPMP

>Danio_Cry2_NP_571861.2

HWFRKGLRLHDNPALQEALNGADTVRCVYILDPWFAGSANVGVNRWRFLLESLEDLDTSLRKLNSRLFVVRGQPTDVFPRLFKEWNVTRLTFEYDSEPYGKERDAAIIKMAQEYGVETVVRNTHTLYNPDRIIEMNNHSPPLTFKRFQAIVNRLELPRKPLPPSLEELGFRTQGDSGGETEALERLNKHLPTGLSPYLRFGCLSCRVFYYNLRD-LFMKLRRRSSPPLSLFGQLLWREFFYTAGTNNPNFDHMEGNPICEALAKWAEGRTGFPWIDAIMTQLRQEGWIHHLARHAVACFLTRGDLWISWESGMKVFEELLLDADWSVNAGSWMWLSCSAQQFFHCYCPVGFGRRTDPSGDYIRRYIPKLKDYPNRYIYEPWNAPESVQKAANCIVGVDYPKP

>Danio_CRY1isoformX1_XP_005166949.1

HWFRKGLRLHDNPALQEAVRGADTVRCVYFLDPWFAGSSNLGVNRWRFLLQCLDDLDSNLRKLNSRLFVVRGQPANVFPRLFKEWKISRLTFEYDSEPFGKERDAAIKKLAMEAGVEVIVKTSHTLYNLDKIIELNGGQPPLTYKRFQTLISRMDPPEMPVEPSLEELGFDIEGLPGGETEALTRIERHLPTGLSPYLRFGCLSCRLFYFKLTD-LYRKVKKTSTPPLSLYGQLLWREFFYTAATTNPRFDKMEGNPICEALAKWAEAKTGFPWIDAIMTQLRQEGWIHHLARHAVACFLTRGDLWISWEEGMKVFEELLLDADWSVNAGSWMWLSCSSQQFFHCYCPVGFGRRTDPNGDFIRRYLPILRGFPAKYIYDPWNAPDSVQAAAKCIIGVHYPKP

>Danio_Cry4_XP_005168334.1

HLFRKGLRLHDNPSLLGALASSSALYPVYVLDRVFLQAMHMGALRWRFLLQSLEDLDTRLQAIGSRLFVLCGSTANILRELVAQWGITQISYDTEVEPYYTRMDKDIQTVAQENGLQTYTCVSHTLYDVKRIVKANGGSPPLTYKKFLHVLSVLGEPEKPARPSLADLGLQVEA--GGESHALQRLEKHFTTGLSPYLSLGCLSVRTFYHRLNS-IYAQSKNHSLPPVSLQGQVLWREFFYTVASATPNFTKMEGNSICERLEKWRTAQTGFPWIDAIMTQLRQEGWIHHLARHAVACFLTRGDLWISWEEGMKVFEEFLLDADYSVNAGNWMWLSASAHKYTRIFCPVRFGRRTDPQGEYLRKYLPVLKNFPSQYIYEPWKAPEDVQLSAGCIIGKDYPRP

>Danio_Cry5_XP_005155462.1

HWFRKGLRLHDNPALIAALKDCRHIYPLFLLDPWFPKNTRIGINRWRFLIEALKDLDSSLKKLNSRLFVVRGSPTEVLPKLFKQWKITRLTFEVDTEPYSQSRDKEVMKLAKEYGVEVTPKISHTLYNIDRIIDENNGKTPMTYIRLQSVVKAMGHPKKPIPPTLEDLGLDTSSLGGGEQEALRRLDEHMTTVLSPYVRFGCLSARTFWWRLAD-VYRGKTH-SDPPVSLHGQLLWREFFYTTAVGIPNFNKMEGNSACEHLAAWREARTGFPFIDAIMTQLRQEGWIHHLARHAVACFLTRGDLWISWEEGQKVFEELLLDSDWSLNAGNWQWLSASTHQYFRVYSPIAFGKKTDKHGDYIKKYLPVLKKFSTEYIYEPWKAPRSVQERAGCIVGKDYPRP

>Capitella_CapteT178510

YWFRRCLRLHDNAALVEVLKEADTFRCIFILDPWFAGASQVGINKWRFLLQSLEDLDSRLRKLNSRLFVIRGQPTDIFPKLFQKWDISALAFEEDPEPFGKERDSAVCTKSQDAGIEVIIKTSHTLFNLQKILDKNSGVPPLTYKRFQRILARMDPPPRPVEPTLEDLGFDTDNLEGGETEALSRLDRHLPTGLSPYLRFGCLSTRLFYWRLTD-LYRKVKKRTDMPLSIHGQLLWREFFYTAATNNPKFDRMVGNPICEALAKWAECKTGFPWIDAIMTQLRQEGWIHHLARHSVACFLTRGDLWISWEEGMKVFEEQLLDADWSINAGMWLWLSCSSQQFFHTYCPASFGRKADPTGDYIRKYLPVLKAFPTKYIYEPWTAPMEIQVAVRCVIGVDYPLP

>Capitella_CapteT226189

HWFRHGLRLHDNPSLMEGLRNCKELYPIFILDGEVAGTGTAGYNRMRFLHQCLEDLDKSFQKFGGRLYIFKGNPVDILAALFDEWQVTKLTFEQDPEPIWEDRDNKVKDLCMKRDVTYVERISHTLFHPDDVIEANGGNPPHTFSLMKQVLNMLGDPERPSSDTLKDFAGEYEMEEGGESRALDLLQGRLPLTLSPHLRFGCLSVRYFYWAIHD-IYSEVR-EELAPQSITSQLIWREYFYIMSVKNRNYAQMENNPICNQLERWEMGKTGYPWIDACMNQLRREGWIHHVGRHAVSCFLTRGDLWIHWEDGLKVFLKYLLDADWSVCAGNWMWVSSSALQCPQCFSPIMYGMRMDPTGEYVRRYVPELRQVPLKYLFHPWKAPKEVQEKAGCIVGTDYPAP

>Daphnia_EFX82092

HWFRKGLRLHDNPSLKDGLKGCSTYRCIFILDPWFAGSSNVDINKWRFLLESLEDLDQNLRKLNSRLFVIRGQPAGVLPKLFKEWETTCLTFEEDPEPFGRVRDQNIITMCKDFNIEVITRASHTLYHPQKIIEKNGGKAPLTYRQFQNIIASVDAPPPPESPTLEELGFDTDGLMGGETEALTRLERHLQTGLSPYLRFGCLSVRLFHQQLTN-LYKKIKK-AQPPLSLHGQVLWREFFYCAATNNPNFDKMIGNPICEALAKWANGQTGFPWIDAIMTQLREEGWIHHLARHAVACFLTRGDLWISWEEGMKVFEELLLDADWSVNAGTWMWLSCSSHQFFHCYCPVRFGRKVDPNGDFIKKYQPVLKNFPLQYIHEPWNAPESVQRAAKCVIGKDYPLP

>Daphnia_EFX85418

HWFRKGLRLHDNPALLNALEKVGELRPVFILDPWFVKNAKVGPNRWRFLVQSLQDLDDNLKKIGSRLFILKGSPTETFKKVFKEWNVKKLTFEVDIEPYAKTRDEEIKKLADHHSVTVVAKVSHTIYDLEKVFKANGNKAPLTYVKFQSVVAKFGTPEKALNPLLEEMQVDLTGLGGGETEALARMEKYMTTVLSPYLKFGCLSPRLMYHRLHE-IIDGRKH-TSPPTSLTGQMLWREFYYTCGAYTPNFNRMVGNPVCEHFVAWKNGRTGYPFIDAIMIQLRTEGWIHHLARHAVACFLTRGDLWVSWELGQQVFEELLLDADWALNAGNWMWLSASAHSYFRVYSPVAFGKKTDKHGDYIKKYLPVLKKFPTEYIYEPWKAPLSVQQTAGCIIGKDYPKR

>Daphnia_EFX77441

LWFRRGLRIHDNPALLSALENSKDFIALFVFDTTFQDPGYKPYHMNGFLLECLHDLNESLESVGTKLHVFQGCPLEVFRHLHNIKPINKLCFIQDCEPIFHERDIAAKNLCSELDIEVYEHVAHTLWDPMDIIASNGGTPPLTYEMFVHVAMSVGDPPKPVAPTLSQIGVPENPVGGGETNALKHFAIRLPLSLSAAISVGAISVRLFYWRIHE-IFDKVN-RGNPPLGITGQIIWRDYFYAMSRMNPKFDKEVDNPICEFFEKWKNGQTGYPFIDAGMRQLNQEGWMHHSVRNAVAMFLTRGDLWLNWDIGAEYMANQLVDSDWSVNSGNWMWVSSSALDCSVCINSVLYGKRLEPSGDYIRRYVPELANFEFEYIHEPWKAPIDIQRTANCIIGQDYPAQ

>Lottia_Hypothetic_XP_009051269.1

LWFRHGLRLKDNPALLAAVKSCNEFYPVFIFDGEVAGTKTAAYPRMKFLVESLNDLDRQFRALGGRLYCFRGDPVKIFTNLFVEWNVSKLTFEVDPEPIWQERDDKVKGLCERHNVDWEEFHSHTLWNPNEIIENNGGHPPLTFDLFKQVSELVGSPARPEPPSCEELGVYCEDEKGGETRGRKLLDSRMPLSLSPHLRFGTVSVREFYWKILD-AYAEVYPNQEAPVSVIAMLIWREYFYTMSVNNLHFNTMKDNPVCEHLDRWTNGKTGFPWVDACMNQLRSEGWLHHVGRHMVSCFITRGDLWLSWEDGLKVFDKYLIDADWSVCAGNWMWVSSSALQCPQCFSPSMYGRRMDPTGEYIRRYVPEIARFPLHFLFEPWKAPLKVQQKAGCIIDKDYPAP

1. **Arrestins tree**

>Argopecten_Arrestin2_ADY16712.1

GVFKKSSPNGKITAYLGKRDFIDHLTHIDPVDGVILVDPEYLKERKVFAHILAAFRYGREDLDVLGLTFKKDLLLASVQVYPPLTRLQERLIKKLGPNAYPFFFELPPNAPASVTLQPAPGDTGKPCGVDYELKTYVAENAEEKPHKRNSVRQAIRKLTYAPVEPAPQPSAEATKEFMMSPGNIKLEASLDKEKYYHGENIAVNVLVDNNTNKTVKKIKISVSQSSE---FSLYEHTCNVLGLEPKEFCKVYHCTPLLTNNRDKRGLALDGKLKHEDTNLASSTIMGDSSQKENLGIVVSYKVKVRLILGDLAVELPFTLDIDDE

>Apis_Phosrestin2_XP_623442.1

RVYKKSSPNNKLTLYLASRDLIVSEAKIDKLQGVLLLDPEFLQGKRVFGQITLTFRYGREDEEVMGLKFCNEAVMCIAQLYPETTPLQEALVKRLGPNAHAFTMEITPLAPPSVQLVPAKEYNGAPIGTSYDVRAYIADRADEKLHRKTTVRMGIRVIQYSEDEENAGPRAAVEKPFLLSDGRVGLEARLDRAIYAHGDPISVHVNVNNSSSKTVRRIKVFIVQHVDVCMFSNGKFKNVVALLSSQEMKKTYTLKPIKGSTKN—WIALEDSYTKAEAMLASTVICPGNEDRNVFAIYVSYYVKVKLLIGDVSLKLPFTLDLIEH

>Apis_BetaArrestin1isoX1_XP_006561894.1

-VFKKSSLNGKITVYLGKRDFVDHITHVDPIDGVVLIDPDYAKDRKVFGHVLAAFKYGREDLDVLGLTFRKDLYLAAEQIYPKLTRLQEKLIKKLGSNAYPFYFELPPHCPASVTLQPAPGDTGKPCGVDYELKAFVGETQDDKPQKRNSVRLAIRKIMYAPSKQGEQPSVEVSKEFVMSPNKLHLEASLDKELYHHGENIAVNVHIANNSNRTVKKIKVSVRQFADICLFSTAQYKCTVAEAESEELSKVFSLKPLLADNKDKWGLALDGQIKDEDTNLASSTLVVDPSQRENLGIIVQYKVKVKLCLGELVAELPFILDIIFE

>Limulus_BetaArr1like_XP_013771701.1

RVFKKSSPNGKITVYLGKRDFVDHMTHVDPIDGVILVDPEYMKERKVFGHVLAAFRYGREDLDLYLASAQIYPLEIATETKQPLTRLQERLIRKLGPNSYPFYFELPPHCPASVTLQPAPGDTGKPCGVDYELKAYVADTEEEKPHKRNSVRLAIRKIMYAPSKQGEQPSVEISKEFMMSPNKLHLEASLDKELYYHGEEIAVNVHVANNSNRTVKKIKVSVRQFADKCSVAEVESEDGCPIGPGFTLSKVFYLKPLLANNKDKRGLALDGQLKHEDTNLASSTIITDPDQKIIVQYKVKDTNLIQLETDQTSGLADQDDDIIFE

>Sof_v-Arr_MN788460

-VFKKTSPNGKLTTYLGKRDYYDHKSWQDHIDGVCFVDPDYIKNRKVFGLIVVAFRYGREDMDVMGVSFRKDFAVKQMQIYPPLTKLQVKLLNKLGEDAIPFHFDLPTNTPDTVCIQPSEYDGGSPCGVDYQITTYVGQNMDDKIHKRNSVSLSIRKLSYFEHTDEPQPREEITKEFKFTSGAMKLECTLDKARYYSGETMNISICVDNPTSKKAKRIKIQVLQLADICLYETLTYKSIVTELETEEFCQVYKLRPFLDMAKRRAGLALDGRVKYEDTMLAATTELSGNVQKENLGVVISYKVRVKMTLGDMVLEVPFKLDIVME

>Idiosepius_v-Arr_A0A0H5ATB5

-VYKKSSPNGKLTTYLAKRDYYDHQKWQDNIDGVCIVDTDYIKNRKVFGIVVVAFRYGREDMDVMGVSFRKDFAVKQIQIYPPLTKLQAKLINKLGEHAVPFHFVLPTTTPDTVCIQPSEFDGGQPCGVDYQITTYVANSMDDKIHKRNSVSLSIRKLSYFEHGQEQQPREEMLKEFKFTSGAMKLECTLDKARYFSGETMNISICVDNPTSKKAKRIKIQIMQFADITLYETVTYKNVVTELETEEFCKVYKLRPVLDAVKRRAGLALNGRVKYEDTTLASSTELTGNVNKENMGVIVSYKVRVKMTLGDMVLEVPFKLDLVME

>Doryteuthis_v-ArrCandidate_comp133665_c0_seq1

-VYKKASPNGKLTTYLAKRDYYDHKEWQDNIDGVCVVDPDYLKNRKVFGLIVVAFRYGREDMDVMGVSFRKDFAVKQMQIYPPLTKLQAKLLNKLGENAVPFHYDLPTNTPDTVCIQPSEYDGGAPCGVDYQVTTYVSQNMDDKIHKRNSVSLSIRKLSYFEFGSDEQPRGEISKEFKFTSGAMKLECTLDKARYYSGESMNISVCVDNPTSKKAKRIKIQIIQLADICLYETVTYKSVVTELETEEFCQVYKLRPVLEVTKRRAGLALNGKVKYEDTMLAASTEDAGNVDKENLGVVVSYKVRIKMTLGDMLLEVPFKLDLIME

>Euprymna_arrestin_ACB05678.1

-VYKKVSPNLKLTTYLGKRDYYDHKSWQDHIDGVCVINTEYLKNRKVFGLIVVAFRYGREDMDVMGVSFRKDFAVKQMQIYPPLTKLQAKLLNKLGEEAIPFHFDLPTNSPDTVCIQPSEYDGGQPCGVDYQITTYVANNMDDKIHKRNSVSLGIRKLTYYEHAEEKQPREEISKEFKFTSGAMKLECTLDKARYYAGETMNISVCVDNTTSKKAKRIKIQVLQMADITLYETVTYKNVVTEIESEEFCQVYKLRPVLDAAKRRAGLALNGRVKYQDTILASSTELTGNVDKENMGVIVNYKVRVKMTLGDMILEIPFKLELVTE

>Obimaculoides_betaArrestin1like_XP_014783971.1

-VYKKSSPNGKLTTYLGRRDYYDHGSWVDNIDGIIVVDPEYLKNRKVFGIIVVAFRYGREDLDVMGMSFRKDFAVKQMQLYPLLTKLQARLLAKIGSNAVPFHFDLPTTTPDTVCIQPCEGDDGAPCGVDYQVTTYVGQSLDEKIHKRNSVSMSIRKLTYLEERKEPQPHGEISKEFKFTSGAMKLECSLDKAKYYSGETININVCVDNPTSKKAKKIRLQVLQYADICLYETVQYKNIVAEVETEEFCKVYKIRPILDAAKRRAGLALNGRVKYEDTALAASTEMAGKVNKENLGIIVNYKIKIKMTLGDMVLEVPFKLDLVME

>Ovulgaris_betaArrestin1like_XP_029637122.1

-VYKKSAPNGKLTTYLGRRDYYDHGTWVDNIDGIIVIDPEYLKNRKVFGIIVVAFRYGREDMDVMGMSFRKDFAVKQMQLYPPLTKLQARLLAKIGSNAVPFHFDLPTTTPDTVCIQPSEGDDGAPCGVDYQVTTYVGQSLDEKIHKRNSVSMSIRKLTYLEDRKEPQPHGEISKEFKFTSGAMKLECSLDKAKYYSGETININVCVDNPTSKKAKKIRLQVLQYADICLYETVQYKNIVAEVETEEFCKVYKIRPILDAAKRRAGLALNGRVKYEDTALAASTEMAGKVNKENLGIIVNYKIKIKMTLGDMVLEVPFKLDLVME

>Nautilus_BetaArrestin_BAR90776.1

-VFKKSSPNGKITAYLGKRDFIDHLTHTDPIDGVILVDPDYLKERKVYAHILAAFRYGREDLDVLGLTFRKDLFIASSQVYPPLTRLQERLIKKLGPNAYPFFFELPKNAPASVTLQPAPGDTGKPCGVDYELKTFVADNVDEKPHKRNSVRLAIRKLTYAPEEPAPQPSAEATKDFMMSPGNIKLEASLDKEKYYHGESLAVNVLVDNNTNKVVKKVKLSVRQFADICLFSTAQYKCTVAELESEEFCKVYHLTPLLANNRDKRGLALDGKLKHEDTNLATSTISPELNAKENLGIVVSYKVKVKLILGDLAVELPFTLDFIFE

>Doryteuthis_BetaArrestinCandidate_comp129748_c0_seq1

-VFKKSSPNGQITAYLGKRDFIDHLSHIDPVDGVILVDPDYLKDRKVYAHILAAFRYGREDLDVLGLTFRKDLFLSSIQVYPPLTRLQGRLIKKLGPNAYPFCFELPHNAPASVTLQPAPGDTGKPCGVDYELKTYVADNVDEKPHKRNSLRLAIRKLTYAPEEPAPQPSAEATKEFMMSPGNMKLEASLDKEKYYHGESLCINVLVDNNTNKSVKKIKILVRQFADICLFSTAQYKCTVAELESEEFCKVYHLTPLLDNNRDKRGLALDGKLKHEDTCLASSTILAESNPKENLGIVVS

YKVKVKLILGDLSVELPFTLDFVFE

>Sof_BetaArrestin_MN788456

-VFKKSSPNGQITAYLGKRDFIDHLTHIDPVDGVILVDPDYLKDRKVYAHILAAFRYGREDLDVLGLTFRKDLFLSSIQVYPPLTRLQGRLIKKLGPNAYPFYFELPHNAPASVTLQPAPGDTGKPCGVDYELKTYVADNVDEKPHKRNSLRLAIRKLTYAPEEPAPQPSAEATKEFMMSPGNMKLEASLDKEKYYHGESLCINVLVDNNTNKSVKKIKILVRQFADICLFSTAQYKCTVAELESEEFCKVYHLTPLLDNNRDKRGLALDGKLKHEDTCLASSTILAESNPKENLGIVVSYKVKVKLILGDLSVELPFTLDFIFE

>Idiosepius_betaArrestin_A0A0H5ANU0

-VFKKSSPNGQITAYLGKRDFIDHLTHIDPVDGVILVDPDYLKDRKVYAHILAAFRYGREDLDVLGLTFRKDLFLSSIQVYPPLTRLQGRLIKKLGPNAYPFYFELPPNAPASVTLQPASGDTGKPCGVDYELKTYVADSVDEKPHKRNSLRLAIRKLTYAPEQPAPQPSAEVTKEFMMSPGNMKLEASLDKEKYYHGESLCINVLVDNNTNKSVKKIKILVRQFADICLFSTAQYKCTVAELESEEFCKVYHLTPLLDNNRDKRGLALDGKLKHEDTCLASSTILAESNPKENLGIVVSYKVKVKLIFGDLSVELPFTLDFIFE

>Aplysia_BetaArrestin1like_XP_012945752.1

-VFKKSSPNGKITTYLGKRDFIDHLSHIDPIDGVILVDPEYLKDRKVFAHILAAFRYGREDLDVLGLTFRKDLYLSSMQVYPPLTRLQERLIKKLGPNAYPFFFELPPNSPASVTLQPAPGDTGKPCGVDYELKTFVADNIDEKPHKRNSVRLAIRKLTYAPEEPAPQPNAEAVKDFIMSPGSIRLEASLDKEKYYHGESIAVNVLVDNNTNKTVKKIKISVRQFADICLFSTAQYKCTVADLESEEFCKVYHLTPLLSNNRDKRGLALDGKLKHEDTNLASSTIMTEKSQKESLGIVVSYRVKVKLIMGELAVELPFTLDFIFE

>Ambigolimax_BetaArrestin_BAX03569.1

-VFKKSCPNGKVTTYLGKRDFIDHLSHIDPIDGVILVDPDYLKDRKVFAHILAAFRYGREDLDVLGLTFRKDLYLSSMQVYPPLTRLQERLIKKLGPNAYPFFFELPPNSPASVTLQPAPGDTGKPCGVDYELKTFVADNIDEKPHKRNSVRLAIRKLTYAPEEPAPQPNAEALKDFIMSPGSMRLEASLDKEKYYHGESIAINVLVDNNTNKTVKKIKISVRQFADICLFSTAQYKCTVADLESEEFCKVFHMTPLLSNNRDKRGLALDGKLKHEDTNLASSTIMTEKSQKESLGIVVSYRVKVKLIMGELAVELPFTLDFIFE

>Pomacea_betaArrestin1like_XP_025080363.1

RVFKKSSPNGKITTYLGKRDFIDHLTHIDPIDGVVLVDPEYLKDRKVYAHILAAFRYGREDLDVLGLTFRKDLYLSSMQVYPPLTRLQERLIKKLGPNAYPFYFELPPNAPASVTLQPAPGDTGKPCGVDYELKTYVADNVDEKPHKRNSVRLAIRKLTYAPEEPAPQPSAEAVKDFMMSPGSIRLEASLDKEKYYHGESIALNVLVDNNTNKTVKKVKLSVRQFADICLFSTAQYKCTVAEMESEEFCKVYHLCPLLTNNRDKRGLALDGKLKHEDTNLASSTIMTEKSQKENLGIVVSYRVKVKLILGDLSVELPFTLDFIFE

>Lottia_HypotheticalProt_XP_009056578.1

RVFKKSSPNGKITTYLGKRDFVDHLTHTDPIDGVVLVDPEYLKDRKVFVHVLAAFRYGREDLDVLGLTFRKDLYLSTMQVFPPLTRLQERLIKKLGPNAYPFYFEMPANAPASVTLQPAPGDTGKPCGVDYELKTFVADNVDEKPHKRNSVRLAIRKLTYAPEEPAPQPSAENVKDFMMSPGNIRLEASLDKEKYYHGESLNVNVLVDNNTNKTVKKIKISVRQFADICLFSTAQYKCTVAELETEEFCKVYHLTPLLANNRDKRGLALDGKLKHEDTNLASSTIMTDSSQKESLGIVVSYRVKIKLILGDLSVELPFTLDFIFE

>Biomphalaria_betaArrestin1like_XP_013067753.1

KCFFVNFVSFQITTYLGKRDFIDHLSHIDPIDGVILVDPEYLKDRKVFAHILAAFRYGREDLDVLGLTFRKDLYLSSMQVYPPLTRLQERLIKKLGPNAYPFFFELPPNSPASVTLQPAPGDTGKPCGVDYELKTFVADNIDEKPHKRNSVRLAIRKLTYAPEEPAPQPNAEAVKDFIMSPGSIRLEASLDKEKYYHGESIAINVLVDNNTNKTVKKIKISVRQFADICLFSTAQYKCTVAELESEEFCKVYHLTPLLLNNRDKRGLALDGKLKHEDTNLASSTIMTEKSQKESLGIVVSYRVKVKLIMGELAVELPFTLDFIFE

>Crassostrea_betaArrestin1like_XP_011433793.1

KVFKKSSPSGKITTYLGKRDFIDHLTHIDPIDGIVLIDKDSLRGRKVFAHVLAAFRYGREDLDVLGLTFRKDLYLANYQVYPPLTNLQERLIRKLGPNAYPFYFELPPNTPASVTLQPAPGDTGKPCGVDYELKTFVAENADEKPHRRNSVRLAIRKLTYAPEAPAPQPSAEAKKDFMMSPGHIKLEASLDKEKYYHGESIAVNVLVDNNTTKSVRKIKISVRQFADICLFSTAQYKCSVAEIDSEEFCKVFYLTPLLSNNRDKRGLALDGKLRHEDTNLASSTIMTESSQKENLGIVVSYRVKVRLILGDLCVELPFTLDFIFE

>Mizuhopecten_betaArrestin1like_XP_021373727.1

RVFKKSSPNGKITAYLGKRDFIDHLTHIDPVDGVILVDPEYLKERKVFAHILAAFRYGREDLDVLGLTFRKDLFLASVQVYPPLTRLQERLIKKLGPNAYPFFFELPPNAPASVTLQPAPGDTGKPCGVDYELKTYVAENAEEKPHKRNSVRLAIRKLTYAPVEPAPQPSAEATKEFMMSPGNIKLEASLDKEKYYHGENIAVNVLVDNNTNKTVKKIKISVRQFADICLFSTAQYKCTVADMEREEFCKVYHCTPLLTNNRDKRGLALDGKLKHEDTNLASSTIMGDSSQKENLGIVVSYKVKVRLILGDLAVELPFTLDAPVD

>Argopecten_Arrestin_ADY16711.1

-VFKKSSPNGKITAYLGKRDFIDHLTHIDPVDGVILVDPECLKERKVFAHILAAFRYGREDLDVLGLTFRKDLFLASVQVYPPLTRLQERLIKKLGPNAYPFFFELPPNAPASVTLQPAPGDTGKPCGVDYELKTYVAENAEKKPHKRNSVRLAIRKLTYAPVEPAPQPSAEATKEFMMSPGNIKLEASLDKEKYYHGENIAVNVLVDNNTNKTVKKIMISVRQFADICLFSTAQYKCTVADMESEEFCKVYHCTPLLTNNRDKRGLALDGKLKHEDTNLASSTIMGDSSQKENLGIVVSYKVKVRLILGDLAVELPFTLDFIFE

>Leptochiton_Arrestin_AMB26727.1

-VFKKSSPNGKITTYLGKRDFIDHLTHTDPIDGVVLVDPEYLKERKVFAHILAAFRYGREDLDVLGLTFRKDLFLASIQVYPPLTRLQERLIKKLGPNAYPFFFELPSNAPASVTLQPAPGDTGKPCGVDYELKTYVADSMDEKPHKRNSVRLAIRKLTYAPEQPAPQPSAESVKEFMMSPGNLKLEASLDKEKYYHGESIAVNVLVDNNT------------------------YSCAVADLESAEFCKVFHMTPLLANNRDKRGLALDGKLKHEDTNLASSTIVGDPSQKENLGIVVSYKVKVRLILGDLAVELPFTLDFIFE

>Capitella_hypotheticProt_ELT93486.1

-VFKKSSPNSKITVYIGKRDFIDHLTHIDPIDGVVLVDPDYLKDRKVFAHVLAAFRYGREDLDVLGLTFRKDLFLASTQVYPTLTRLQERLIQKLGPNAYPFFFELPACSPASVTLQPAPGDTGKPCGVDYELKTYVAENQDDKPHKRNSVSLAIRKLTYAPEEPAPQPSAEATKEFMMSQGNLRLECSLEKEKYYHGESIAVNVLVDNNSNKSVKKVKISVRQYADICLFSTAQYKCVVADLETEEFCKVYYLTPLLKNNRDKRGLALDGKLKHEDTNLASSTIITDQNQKENLGIVVQYKVKVRLILGDLSVELPFTLDFIFE

>Helobdella_hypotheticProt_XP_009023692.1

-VFKKSCPNSKITVYLGKRDFMDYLDHVEPIDGVVLVDPDYLKDRKVFAQILAAFRYGREDLDVLGLTFRKDLLLATKQVYPSLTRLQERLIKKLGPNAFPFCFEIPTSSPASVTLQPAPGDTGKPCGVDYELKAYVASEPDEKPHRRNSVQLAIRKLTFAPEQPAPQPSAEAVKEFLMSSGTLRLEATLDKEKYYHGESITVNVLVDNNAGRSVKKIKISVRQYADICLFSTAQYKCNVAEIDSEEFCKVYQLTPILSNNRDKRGLALDGKLKHEDTNLASSTIITSNSQKENLGIVVQYKVKVRLILGDLSVELPFTLDIIFE

>Xenopus_betaArrestin2_NP_001093721.1

-VFKKSSPNCKLTVYLGKRDFVDHLDRVDPVDGVVLVDTDYLKDRKVYVTLTCAFRYGREDLDVLGLSFRKDLFISKFQAYPPLTRLQERLIKKLGEQAHPFFFTIPQNLPCSVTLQPGPEDTGKACGVDYEIRAFCAKTMEEKMHKRNSVRLVIRKVQFAPEKPGPQPVAETTRHFLMSDRSLHLEASLDKELYYHGEPINVNVHVTNNSSKTVKRVKVSVRQYADICLFSTAQYKCPVAQIEQDDFCKVYTLTPLLSNNREKRGLALDGKLKHEDTNLASSTIVKEGSSKEVLGILVSYRVKVKLVV-DVAVELPFVLDIVFE

>Danio_betaArrestin2_NP_999846.1

-VFKKSSPNCKVTVYLGKRDFVDHLDHVDPVDGVILVDPEYLKDRKVFVTLTCAFRYGREDLDVLGLSFRKDLYIFTFQAYPPHSRLQERLLKKLGQNAYPFHFSIPQNLPCSVTLQPGPEDTGKACGVDFEIRAFCAKSMEEKNHKRNSVRLVIRKAQYAPEKPGPQPMVETTRSFLMSDRSLHLEASLDKELYYHGEPISVNVHVTNNSTKTVKRVKISVRQYADICLFSTAQYKCPVAQIEADDFCKVYTLTPTLNNNREKRGPALDGKLKHEDTNLASSTIVKDVSNKEVLGVLVSYRVKVKLVV-DVSVELPFVLDIVFE

>Homo_betaArrestin2_P32121

-VFKKSSPNCKLTVYLGKRDFVDHLDKVDPVDGVVLVDPDYLKDRKVFVTLTCAFRYGREDLDVLGLSFRKDLFIATYQAFPPVTRLQDRLLRKLGQHAHPFFFTIPQNLPCSVTLQPGPEDTGKACGVDFEIRAFCAKSLEEKSHKRNSVRLVIRKVQFAPEKPGPQPSAETTRHFLMSDRSLHLEASLDKELYYHGEPLNVNVHVTNNSTKTVKKIKVSVRQYADICLFSTAQYKCPVAQLEQDDFCKVYTITPLLSDNREKRGLALDGKLKHEDTNLASSTIVKEGANKEVLGILVSYRVKVKLVV-DVSVELPFVLDIVFE

>Danio_betaArrestin_NP_001153294.1

-VFKKASPNGKLTVYLGKRDFVDHVDLVEPVDGVVLIDPEYLKERKVFVTLTCAFRYGREDLDVLGLTFRKDLFVANIQAFPSLTRLQERLIKKLGEHAYPFTFEIPPNLPCSVTLQPGPEDTGKACGVDFEVKAFCAENVEEKIHKRNSVRLVIRKVQYAPEKPGPQPMAETTRQFLMSDKPLHLEASLDKEIYYHGEPISVNVHVTNNTNKTVKKIKISVRQYADICLFNTAQYKCPVAIEESDDFCKVYTLTPFLANNREKRGLALDGKLKHEDTNLASSTLLREGANKEILGIIVSYKVKVKLVV-DVAVELPFTLDIIFE

>Homo_betaArrestin1isoB_NP_064647.1

RVFKKASPNGKLTVYLGKRDFVDHIDLVDPVDGVVLVDPEYLKERRVYVTLTCAFRYGREDLDVLGLTFRKDLFVANVQSFPPLTRLQERLIKKLGEHAYPFTFEIPPNLPCSVTLQPGPEDTGKACGVDYEVKAFCAENLEEKIHKRNSVRLVIRKVQYAPERPGPQPTAETTRQFLMSDKPLHLEASLDKEIYYHGEPISVNVHVTNNTNKTVKKIKISVRQYADICLFNTAQYKCPVAMEEADDFCKVYTLTPFLANNREKRGLALDGKLKHEDTNLASSTLLREGANREILGIIVSYKVKVKLVVGDVAVELPFTLDIVFE

>Danio_ArrestinC_NP_957086.1

-VYKKTSGNGSLCLYLGRRDFVDHVESVDSVDGVLKIDPSGLNGRKVWVQLACAFRYGREDLDVIGVSFRKDIWIKRIQMYPPNTPMQEALLKKAGDQGHPFTFDIPVHLPCSVSLQPAPEDAGKPCGVDYEVKAYIADNIDEKVEKKDTCRLIIRKIQYAPAELAAGPKADINKQFITADKPIHMEVSMEKELYYHGDPIPIKVKVNNETSKVVKKIKINIFQITDVVIYAADKYHKCVLNEEFGDFEKEYSVTPLLVNNKEKRGLALDGRLKDEDTNLASSTLLIPDMDKQMQGVVVSYKIKVILMMGDVTAELPLVLDINLE

>Homo_ArrestinC_P36575

-VFKKTSSNGKLSIYLGKRDFVDHVDTVEPIDGVVLVDPEYLKCRKLFVMLTCAFRYGRDDLEVIGLTFRKDLYVQTLQVVPAETVLQERLLHKLGDNAYPFTLQMVTNLPCSVTLQPGPEDAGKPCGIDFEVKSFCAENPEETVSKRDYVRLVVRKVQFAPPEAGPGPSAQTIRRFLLSAQPLQLQAWMDREVHYHGEPISVNVSINNCTNKVIKKIKISVDQITDVVLYSLDKYTKTVFIQEFTEFSQSFAVTPILAASCQKRGLALDGKLKHEDTNLASSTIIRPGMDKELLGILVSYKVRVNLMV-DVGVELPLVLDIVIE

>Danio_arrestinS_NP_956853.1

-VFKKISKDKSVGVYMGKRDFVDRVDSVDPVDGVILIDPEQLRGKKAYVTLSCVFRYGRDDAEVLGISFRKEIYISTRQVYPILTKVQEKLLRKLGDNAYPFFFEFPDNLPCSVGLQPAPKDVGKHCAVEFEVKAFCAESQDAKVRKRSSVGLMIRKVQYAPEKLGPAPSVETTRDFLMSDKPLHLEASLEKQTYYHGEPINVRVKINNQSNKNVRNIILSVEQNANVVLYCNDNYMKVVATEDSGHLEKVYTLLPLLANNRERRGIALDGKLKHEDTNLASSSIIKEGVQKEVLGIMVSYRVVVKLIVGEVGVELPFQLEMVFE

>Homo_ArrestinS_P10523

-IFKKISRDKSVTIYLGNRDYIDHVSQVQPVDGVVLVDPDLVKGKKVYVTLTCAFRYGQEDIDVIGLTFRRDLYFSRVQVYPPVTKLQESLLKKLGSNTYPFLLTFPDYLPCSVMLQPAPQDSGKSCGVDFEVKAFATDSTDDKIPKKSSVRLLIRKVQHAPLEMGPQPRAEAAWQFFMSDKPLHLAVSLNKEIYFHGEPIPVTVTVTNNTEKTVKKIKAFVEQVANVVLYSSDYYVKPVAMEEAQELTKTLTLLPLLANNRERRGIALDGKIKHEDTNLASSTIIKEGIDRTVLGILVSYQIKVKLTVSEVATEVPFRLNLVFE

>Ovulgaris_betaArrestin1like_XP_029636254.1

-VFKKSSPNGQITAYLGKRDFIDHLSHIDPVDGVILVEPDYLKDRKVYAHILAAFRYGREDLDVLGLTFRKDLFLSSVQVYPPLTRLQGRLIKKLGPNAYPFYFELPPNAPASVTLQPAPGDTGKPCGVDYELKTYVADHVDEKPHKRNSLRLAIRKLTYAPEEPAPQPSAENTKEFMMSPGNMKLEASLDKEKYYHGESLCINVLVDNNTNKSVKKIKISVRQVADICLFSTAQYKCTVAELESEEFCKVYHLTPLLDNNRDKRGLALDGKLKHEDTCLASSTILVESNPKENLGIVVSYKVKVKLILGDLSVELPFTLDFIFE

>Obimaculoides_betaArrestin1like_XP_014770289.1

-VFKKSSPNGQITAYLGKRDFIDHLSHIDPVDGVILVEPDYLKDRKVYAHILAAFRYGREDLDVLGLTFRKDLFLSSVQVYPPLTRLQGRLIKKLGPNAYPFYFELPPNAPASVTLQPAPGDTGKPCGVDYELKTYVADHVDEKPHKRNSLRLAIRKLTYAPEEPAPQPSAENTKEFMMSPGNMKLEASLDKEKYYHGESLCINVLVDNNTNKSVKKIKISVRQVADICLFSTAQYKCTVAELESEEFCKVYHLTPLLDNNRDKRGLALDGKLKHEDTCLASSTILVESNPKENLGIVVSYKVKVKLILGDLSVELPFTLDFIFE

>Limulus_Arrestin_P51484

KVFKKTAPNGKITVYLGKRDFGDHGSYCEPVEGVLLVDNEYLKGRKVFGQVTTTFRYGREEDEVMGLHFSRQLYLALEQVLPAPSDFQNRLVRKLGTLAHPFTFALPENAPPSVTLQPGSEDQGRPLGVEYELKLFIAETEDEKPHKRNSVSMAIRKLQYAKPSPLAQPSALVSKGFMMSSGKLQLEVTLDKELYFHGDKVSANVTISNYSKKTVKNIKVAVVQNTEVTMV-NGHFHKTISSIESKELSKVYTLLPLASQNKDKRGIALDGMLKEGDTNLASSTL---NSTGDAIGIVISYVIRVRLYMGELVADVSFKLDLIVE

>Drosophila_ArrB_P19107

KVFKKATPNGKVTFYLGRRDFIDHIDYCDPVDGVIVVEPDYLKNRKVFGQLATTYRYGREEDEVMGVKFSKELILCREQIVPEMTPMQEKLVRKLGSNAYPFTFHFPPNSPSSVTLQQEGDDNGKPLGVEYTIRAFVGDSEDDRQHKRSMVSLVIKKLQYAPLNRGQLPSSLVSKGFTFSNGKISLEVTLDREIYYHGEKTAATVQVSNNSKKSVKSIKCFIVQHTEITMV-NAQFSKHVAQLETKELTKTFYLIPLAANNKDRHGIALDGHLKDEDVNLASSTMVQEKSTGDACGIVISYSVRIKLNCGEMQTDVPFKLNIVFE

>Drosophila_ArrA_P15372

KVFKKCSPNNMITLYMNRRDFVDSVTQVEPIDGIIVLDDEYVRNRKIFVQLVCNFRYGREDDEMIGLRFQKELTLVSQQVCPQLTKMQERLLKKLGSNAYPFVMQMPPSSPASVVLQQKASDESQPCGVQYFVKIFTGDSDCDRSHRRSTINLGIRKVQYAPTKQGIQPCTVVRKDFLLSPGELELEVTLDKQLYHHGEKISVNICVRNNSNKVVKKIKAMVQQGVDVVLFQNGQFRNTIAFMETSELQKVMYLVPTLVANCDRAGIAVEGDIKRKDTALASTTLIASQDARDAFGIIVSYAVKVKLFLGELCAELPFILQLEAE

>Drosophila_KurtzArrestin_Q9V393

RVFKKSSSNGKITVYLGKRDFVDHVTHVDPIDGVVFIDPEYVKDRKVFGQVLAAFRYGREDLDVLGLTFRKDLYLAHEQIYPPMTRLQERLIKKLGPNAHPFYFEVPPYCPASVSLQPAPGDVGKSCGVDYELKAFVGENVEDKPHKRNSVRLTIRKVMYAPSKVGEQPSIEVSKEFMMKPNKIHLEATLDKELYHHGEKISVNVHVANNSNRTVKKIKVCVRQFADICLFSTAQYKSVVAEIESEDLSKVFELCPLLANNKDKWGLALDGQLKHEDTNLASSTLITNPAQRESLGIMVHYKVKVKLLIGDLVAELPFTLDIIFE

**S3: Prediction of transmembrane helices in opsin proteins (TMHMM output)**

1. **Sof_ropsin1**

# WEBSEQUENCE Length: 464

# WEBSEQUENCE Number of predicted TMHs: 6

Idem pour la sequence publiée de *Sepia officinalis*

# WEBSEQUENCE Exp number of AAs in TMHs: 137.41589

# WEBSEQUENCE Exp number, first 60 AAs: 23.15328

# WEBSEQUENCE Total prob of N-in: 0.00346

# WEBSEQUENCE POSSIBLE N-term signal sequence

WEBSEQUENCE TMHMM2.0 outside 1 36

WEBSEQUENCE TMHMM2.0 TMhelix 37 59

WEBSEQUENCE TMHMM2.0 inside 60 71

WEBSEQUENCE TMHMM2.0 TMhelix 72 94

WEBSEQUENCE TMHMM2.0 outside 95 108

WEBSEQUENCE TMHMM2.0 TMhelix 109 131

WEBSEQUENCE TMHMM2.0 inside 132 151

WEBSEQUENCE TMHMM2.0 TMhelix 152 174

WEBSEQUENCE TMHMM2.0 outside 175 201

WEBSEQUENCE TMHMM2.0 TMhelix 202 224

WEBSEQUENCE TMHMM2.0 inside 225 261

WEBSEQUENCE TMHMM2.0 TMhelix 262 284

WEBSEQUENCE TMHMM2.0 outside 285 464


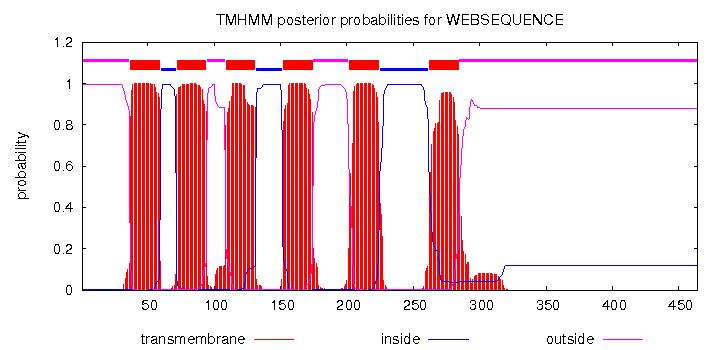


# [plot](http://www.cbs.dtu.dk/services/TMHMM-2.0/tmp/TMHMM_2208/WEBSEQUENCE.eps) in postscript, [script](http://www.cbs.dtu.dk/services/TMHMM-2.0/tmp/TMHMM_2208/WEBSEQUENCE.gnuplot) for making the plot in gnuplot, [data](http://www.cbs.dtu.dk/services/TMHMM-2.0/tmp/TMHMM_2208/WEBSEQUENCE.plp) for plot

1. **Sof_ropsin2**

# WEBSEQUENCE Length: 409

# WEBSEQUENCE Number of predicted TMHs: 7

# WEBSEQUENCE Exp number of AAs in TMHs: 156.30732

# WEBSEQUENCE Exp number, first 60 AAs: 14.30764

# WEBSEQUENCE Total prob of N-in: 0.00024

# WEBSEQUENCE POSSIBLE N-term signal sequence

WEBSEQUENCE TMHMM2.0 outside 1 46

WEBSEQUENCE TMHMM2.0 TMhelix 47 69

WEBSEQUENCE TMHMM2.0 inside 70 80

WEBSEQUENCE TMHMM2.0 TMhelix 81 103

WEBSEQUENCE TMHMM2.0 outside 104 117

WEBSEQUENCE TMHMM2.0 TMhelix 118 140

WEBSEQUENCE TMHMM2.0 inside 141 160

WEBSEQUENCE TMHMM2.0 TMhelix 161 183

WEBSEQUENCE TMHMM2.0 outside 184 210

WEBSEQUENCE TMHMM2.0 TMhelix 211 233

WEBSEQUENCE TMHMM2.0 inside 234 271

WEBSEQUENCE TMHMM2.0 TMhelix 272 294

WEBSEQUENCE TMHMM2.0 outside 295 303

WEBSEQUENCE TMHMM2.0 TMhelix 304 326

WEBSEQUENCE TMHMM2.0 inside 327 409


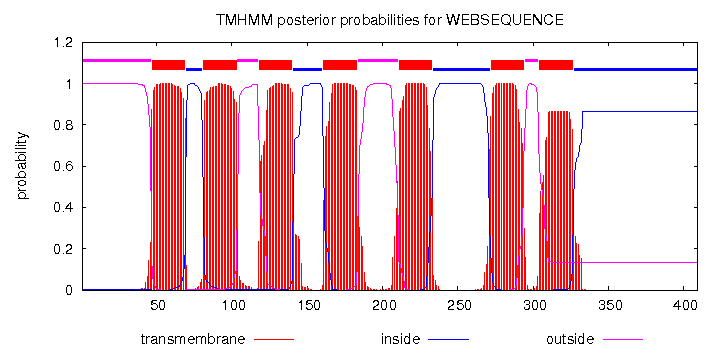


1. **Sof_reti1**

# WEBSEQUENCE Length: 301

# WEBSEQUENCE Number of predicted TMHs: 7

# WEBSEQUENCE Exp number of AAs in TMHs: 154.74104

# WEBSEQUENCE Exp number, first 60 AAs: 28.95995

# WEBSEQUENCE Total prob of N-in: 0.00002

# WEBSEQUENCE POSSIBLE N-term signal sequence

WEBSEQUENCE TMHMM2.0 outside 1 19

WEBSEQUENCE TMHMM2.0 TMhelix 20 42

WEBSEQUENCE TMHMM2.0 inside 43 54

WEBSEQUENCE TMHMM2.0 TMhelix 55 77

WEBSEQUENCE TMHMM2.0 outside 78 91

WEBSEQUENCE TMHMM2.0 TMhelix 92 111

WEBSEQUENCE TMHMM2.0 inside 112 130

WEBSEQUENCE TMHMM2.0 TMhelix 131 153

WEBSEQUENCE TMHMM2.0 outside 154 179

WEBSEQUENCE TMHMM2.0 TMhelix 180 202

WEBSEQUENCE TMHMM2.0 inside 203 229

WEBSEQUENCE TMHMM2.0 TMhelix 230 252

WEBSEQUENCE TMHMM2.0 outside 253 266

WEBSEQUENCE TMHMM2.0 TMhelix 267 289

WEBSEQUENCE TMHMM2.0 inside 290 301


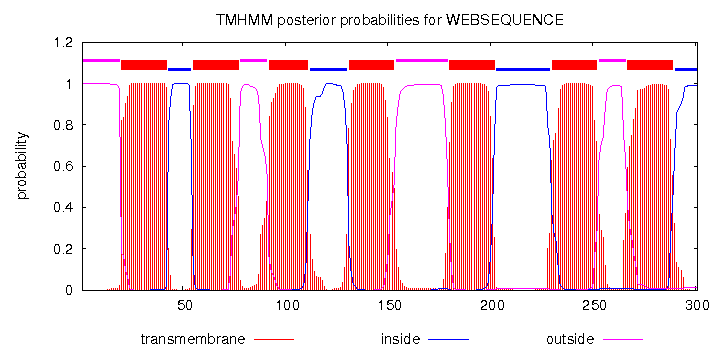


1. **Sof_reti2**

# WEBSEQUENCE Length: 304

# WEBSEQUENCE Number of predicted TMHs: 7

# WEBSEQUENCE Exp number of AAs in TMHs: 156.48275

# WEBSEQUENCE Exp number, first 60 AAs: 24.65047

# WEBSEQUENCE Total prob of N-in: 0.00021

# WEBSEQUENCE POSSIBLE N-term signal sequence

WEBSEQUENCE TMHMM2.0 outside 1 24

WEBSEQUENCE TMHMM2.0 TMhelix 25 47

WEBSEQUENCE TMHMM2.0 inside 48 59

WEBSEQUENCE TMHMM2.0 TMhelix 60 79

WEBSEQUENCE TMHMM2.0 outside 80 93

WEBSEQUENCE TMHMM2.0 TMhelix 94 116

WEBSEQUENCE TMHMM2.0 inside 117 135

WEBSEQUENCE TMHMM2.0 TMhelix 136 158

WEBSEQUENCE TMHMM2.0 outside 159 181

WEBSEQUENCE TMHMM2.0 TMhelix 182 204

WEBSEQUENCE TMHMM2.0 inside 205 230

WEBSEQUENCE TMHMM2.0 TMhelix 231 253

WEBSEQUENCE TMHMM2.0 outside 254 267

WEBSEQUENCE TMHMM2.0 TMhelix 268 290

WEBSEQUENCE TMHMM2.0 inside 291 304


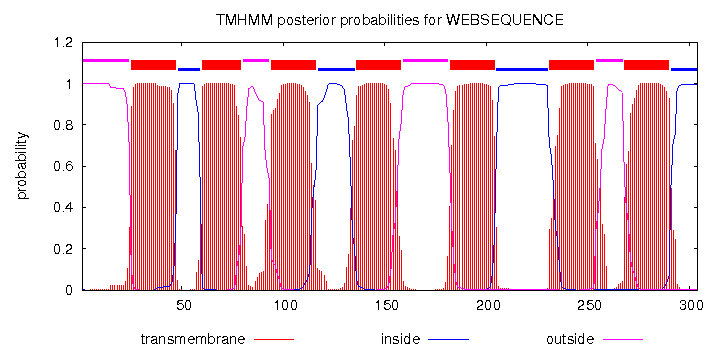


1. **Sof_xeno1**

# WEBSEQUENCE Length: 430

# WEBSEQUENCE Number of predicted TMHs: 7

# WEBSEQUENCE Exp number of AAs in TMHs: 154.93182

# WEBSEQUENCE Exp number, first 60 AAs: 10.88017

# WEBSEQUENCE Total prob of N-in: 0.00704

# WEBSEQUENCE POSSIBLE N-term signal sequence

WEBSEQUENCE TMHMM2.0 outside 1 49

WEBSEQUENCE TMHMM2.0 TMhelix 50 72

WEBSEQUENCE TMHMM2.0 inside 73 83

WEBSEQUENCE TMHMM2.0 TMhelix 84 106

WEBSEQUENCE TMHMM2.0 outside 107 120

WEBSEQUENCE TMHMM2.0 TMhelix 121 143

WEBSEQUENCE TMHMM2.0 inside 144 162

WEBSEQUENCE TMHMM2.0 TMhelix 163 185

WEBSEQUENCE TMHMM2.0 outside 186 209

WEBSEQUENCE TMHMM2.0 TMhelix 210 232

WEBSEQUENCE TMHMM2.0 inside 233 262

WEBSEQUENCE TMHMM2.0 TMhelix 263 285

WEBSEQUENCE TMHMM2.0 outside 286 294

WEBSEQUENCE TMHMM2.0 TMhelix 295 317

WEBSEQUENCE TMHMM2.0 inside 318 430


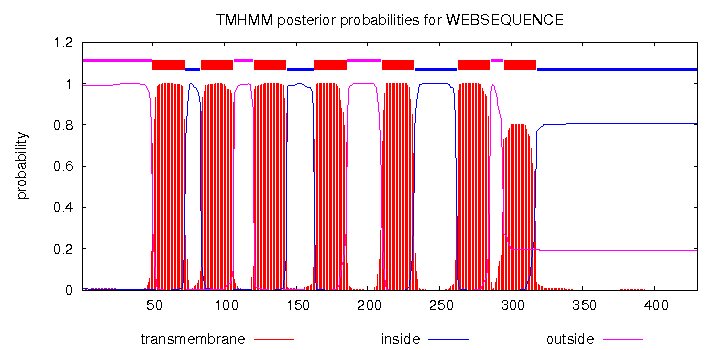


1. **Sof_xeno2**

# WEBSEQUENCE Length: 432

# WEBSEQUENCE Number of predicted TMHs: 7

# WEBSEQUENCE Exp number of AAs in TMHs: 154.00914

# WEBSEQUENCE Exp number, first 60 AAs: 15.80551

# WEBSEQUENCE Total prob of N-in: 0.00022

# WEBSEQUENCE POSSIBLE N-term signal sequence

WEBSEQUENCE TMHMM2.0 outside 1 42

WEBSEQUENCE TMHMM2.0 TMhelix 43 65

WEBSEQUENCE TMHMM2.0 inside 66 85

WEBSEQUENCE TMHMM2.0 TMhelix 86 108

WEBSEQUENCE TMHMM2.0 outside 109 122

WEBSEQUENCE TMHMM2.0 TMhelix 123 145

WEBSEQUENCE TMHMM2.0 inside 146 164

WEBSEQUENCE TMHMM2.0 TMhelix 165 187

WEBSEQUENCE TMHMM2.0 outside 188 210

WEBSEQUENCE TMHMM2.0 TMhelix 211 233

WEBSEQUENCE TMHMM2.0 inside 234 264

WEBSEQUENCE TMHMM2.0 TMhelix 265 287

WEBSEQUENCE TMHMM2.0 outside 288 296

WEBSEQUENCE TMHMM2.0 TMhelix 297 319

WEBSEQUENCE TMHMM2.0 inside 320 432


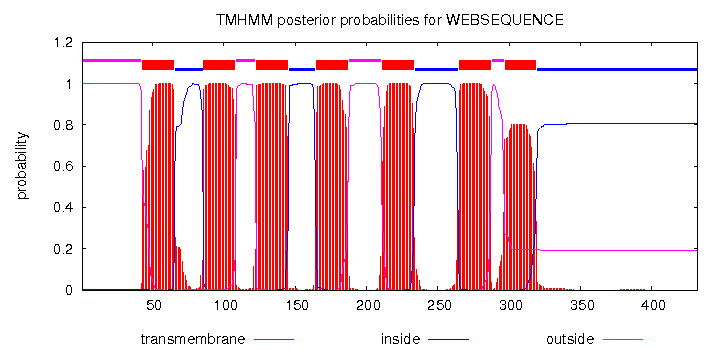


**S4: Alignment of photosensitive molecules with emphasis on important features**

1. **Opsins**


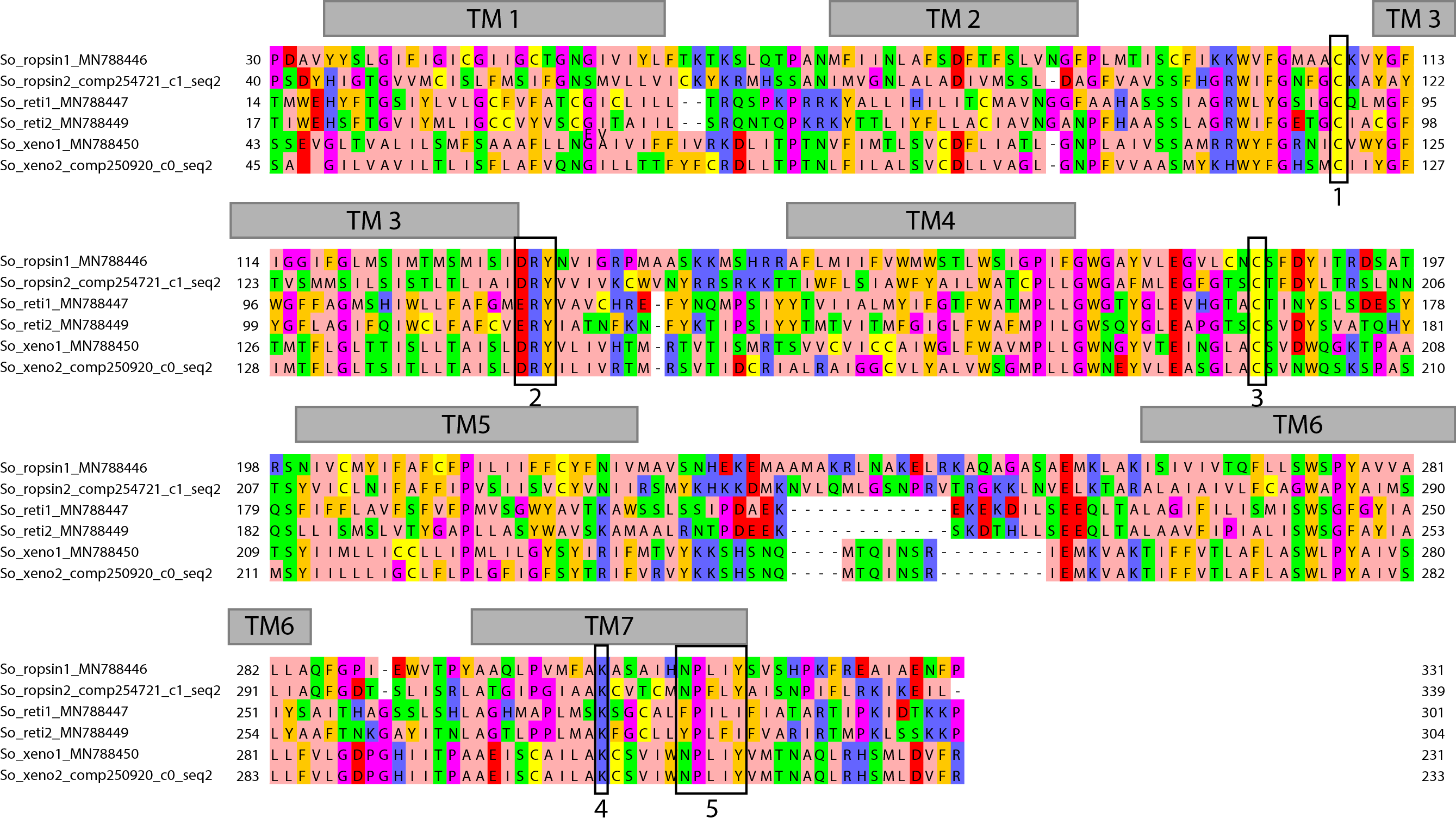


1. **Cryptochromes**

**
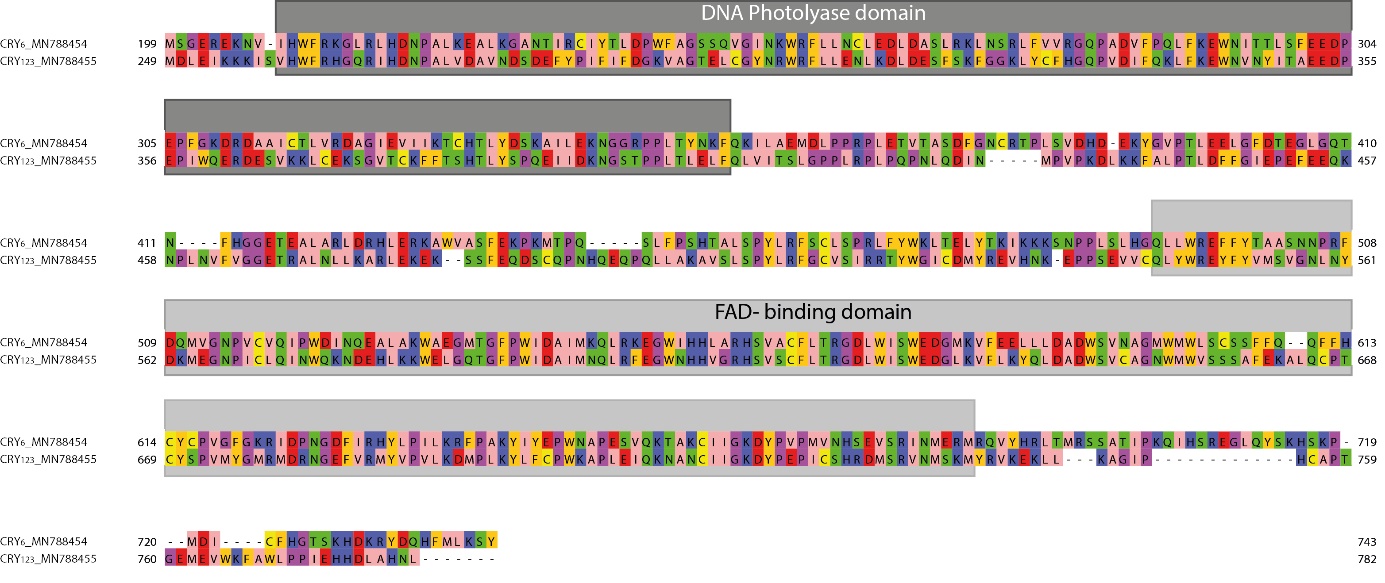
**
